# Testing the Luedemann hypothesis: the discovery of novel antimicrobials from slow-growing microbes from nutrient-limited environments

**DOI:** 10.1128/msphere.00367-25

**Published:** 2025-09-23

**Authors:** Brendan Lin, Sunmin Woo, Alesa Philbrick, John Bacsa, Emily Laskey, Nancy Mehra, Vijay S. Gondil, Jia A. Mei, George Jones, Martin S. Pavelka, Michelle Dziejman, Daniel A. Shutter, Christian Melander, Alexander M. Perritt, Rick Jakober, Yaoyao Shen, Wei-Chen Chang, Cassandra L. Quave, Paul M. Dunman, George Luedemann

**Affiliations:** 1 Department of Microbiology and Immunology, University of Rochester Medical Center 6923, Rochester, New York, USA; 2 Center for the Study of Human Health, Emory University College of Arts and Sciences 169280, Atlanta, Georgia, USA; 3 Department of Chemistry, Emory University College of Arts and Sciences 169280, Atlanta, Georgia, USA; 4 Department of Chemistry and Biochemistry, Notre Dame University 6111, South Bend, Indiana, USA; 5 Perritt Laboratories 12299, Hightstown, New Jersey, USA; 6 Department of Chemistry, North Carolina State University 6798 , Raleigh, North Carolina, USA; 7 Department of Dermatology, Emory University School of Medicine 12239, Atlanta, Georgia, USA; 8 Independent Researcher, Amado, Arizona, USA; University of Nebraska Medical Center College of Medicine, Omaha, Nebraska, USA

**Keywords:** natural product, antimicrobial, *Acinetobacter baumannii*

## Abstract

George Luedemann is known throughout the antimicrobial community as one of the discoverers of the natural product antibiotic gentamicin. He subsequently hypothesized that slow-growing organisms inhabiting inhospitable, nutrient-limited environments may represent an enriched source of previously undescribed microbes that produce novel antimicrobials to create a competitive advantage over faster-growing rival organisms. Accordingly, 750 slow-growing microorganisms were isolated from desert rock surfaces and archived prior to Dr. Luedemann’s passing in 2000. Here, we describe the characterization and antimicrobial screening of the first 147 members of the Luedemann collection. 16S rRNA and whole-genome sequencing revealed that the pilot isolate set is highly diverse and includes novel microbial species belonging to genera commonly associated with soil samples, including *Geodermatophilus*, *Streptomyces*, and *Micromonospora*. Antimicrobial screening and comparative genomics indicate that at least six members are likely to produce novel antimicrobials with activity toward the ESKAPE pathogens, *Vibrio cholerae* and/or *Mycobacterium smegmatis*. Indeed, we show that the library member “9005BA” produces a newly identified phenazine, pyocyanin A, which displays potent (0.625 µg/mL), selective bactericidal activity toward *Acinetobacter baumannii* and efficacy in animals. Genetic and biochemical assays revealed that the antimicrobial activity of pyocyanin A is likely to be mediated by oxidative stress and can be overcome by altering bacterial respiration and/or efflux. Taken together, the data suggest that slow-growing organisms inhabiting nutrient-limited environments represent a previously overlooked rich source of microbial and antimicrobial agent diversity.

**IMPORTANCE:**

The discovery and study of novel bacterial species offer an opportunity to identify new microbial biological processes, molecular mechanisms, and secondary metabolites, such as new antibiotics. Our work indicates that slow-growing organisms inhabiting nutrient-limited environments may represent an enriched source of novel microbial species. Furthermore, we find that a subset of these organisms is likely to produce corresponding novel antimicrobials, presumably as a means to outcompete faster-growing rival organisms. Indeed, we show that a putative new *Streptomyces* species is capable of producing a previously undescribed antimicrobial, pyocyanin A, with potent, selective antibacterial toward *Acinetobacter baumannii*, a prominent cause of antibiotic-resistant infections.

## INTRODUCTION

Antimicrobial drug resistance (AMR) is a global healthcare crisis. In 2019 alone, 1.24 million deaths were attributed to AMR infections worldwide, and such infections are projected to eclipse cancer as an annual cause of death globally by the year 2050 (1, 2). Six bacterial species, termed the ESKAPE (*Enterococcus faecalis*, *Staphylococcus aureus*, *Klebsiella pneumoniae*, *Acinetobacter baumannii*, *Pseudomonas aeruginosa*, and *Enterobacter* spp.) pathogens, are particularly problematic as they are the predominant causes of U.S. nosocomial infections and “escape” the antimicrobial effects of front-line antibiotics due to AMR (3). While AMR has been a long-standing problem for the ESKAPEs and other pathogens, such as *Mycobacterium tuberculosis*, multidrug antibiotic resistance is also rapidly emerging in non-tubercular mycobacteria, *Vibrio cholerae*, and other organisms, accentuating the need for new antibiotics for the therapeutic intervention of bacterial infection(s) (4, 5).

Most antibiotics are products or semi-synthetic derivatives of chemicals that are naturally produced by environmental microbes, presumably to create a competitive advantage over rival microorganisms (6, 7). In that regard, the golden age of antibiotic drug discovery began with the isolation of the natural product antibiotics actinomycin, streptothricin, and streptomycin from soil-dwelling *Streptomyces* by Waksman and colleagues in the 1940s (8–10). The resulting Waksman platform of propagation and isolation of novel antimicrobials produced by relatively easily culturable soil microorganisms led to intense natural product exploration of the *Streptomyces* and led to 80% of today’s antibiotics (11). Following this approach, Luedemann and colleagues expanded the Waksman platform to include soil *Micromonospora* and developed one of the most successfully used antibiotics in modern medicine, gentamicin (12). While highly successful, such soil-derived natural product antimicrobial exploration efforts eventually began to lead to the rediscovery of already identified antibiotics (reviewed in reference 13). This, combined with Food and Drug Administration regulatory variability and ineffective antibiotic research advocacy campaigns, subsequently led to the downsizing or outright elimination of most pharmaceutical antimicrobial discovery teams.

Upon retiring to the U.S. Southwest, Dr. Luedemann developed the hypothesis that slow-growing microbes inhabiting harsh, nutrient-limited niches may produce antimicrobial agents to gain a competitive advantage over faster-growing organisms. Moreover, he predicted that because most previous natural product discovery campaigns focused on fast-growing, easily culturable environmental organisms, slow-growing organisms may represent a previously unappreciated and untapped source of novel microbial and corresponding natural product antimicrobial diversity. To test this hypothesis, Dr. Luedemann collected and archived approximately 750 slow-growing microbials; however, he was unable to test his underlying hypothesis that these microbes produce new antimicrobial agents before passing away in 2000. We obtained this library of bacterial strains, known as the “Luedemann collection.” Here, we describe the early characterization of the first 147 members of the Luedemann collection and the discovery of a new phenazine antimicrobial compound with selective, potent antibacterial activity against the ESKAPE pathogen, *A. baumannii*, providing preliminary validation of the “Luedemann hypothesis.”

## RESULTS AND DISCUSSION

### Luedemann collection

**Fig 1:**
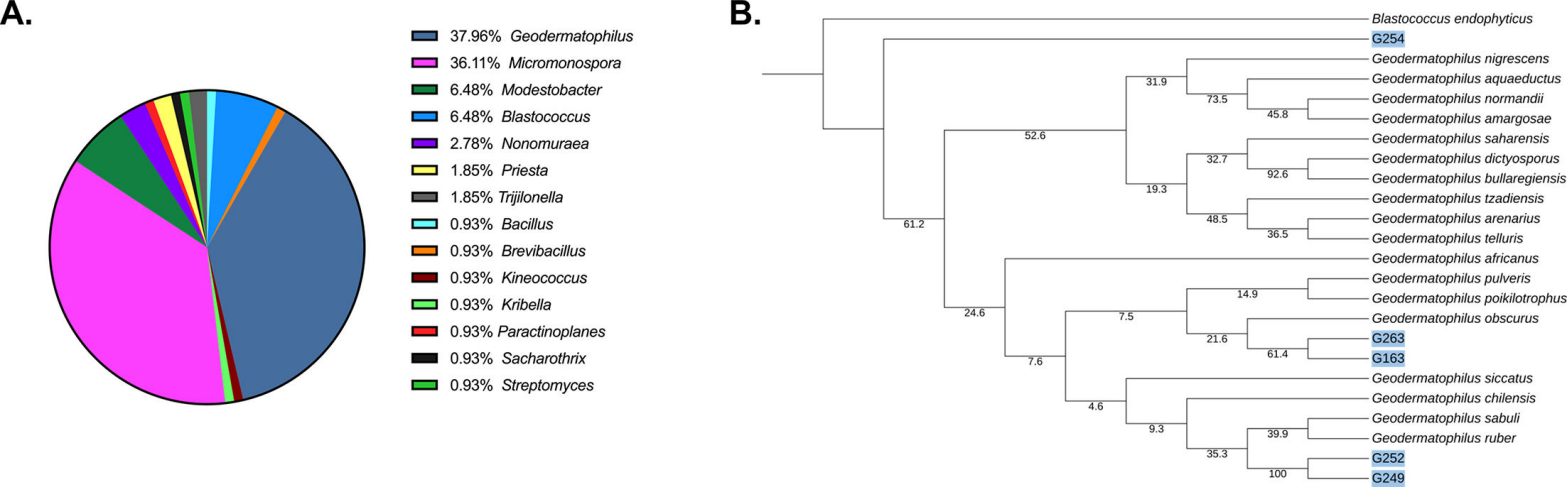
Luedemann collection. 16S rRNA predicted microbial composition of the Luedemann collection (**A**). Phylogenetic tree containing known and predicted novel (highlighted in blue) *Geodermatophilus* spp. within the Luedemann collection (**B**).

To assess the microbial composition of the collection, each member’s 16S rRNA sequence was determined and compared to National Center for Biotechnology Information (NCBI) databases using BLAST (14). While sequencing results were not achievable for all members of the collection, 125 isolates were found to share 94.8%–100% 16S rRNA sequence identity with bacterial NCBI entries (Data File S1). As shown in Fig. 1A, a survey of similarity searches revealed that the majority (81.6%) of the collection is composed of five genera belonging to the *Actinomycetes* class of bacteria that are commonly associated with soil or rock surfaces. These include 6 *Blastococcus* spp. isolates, 57 *Geodermatophilus* spp. isolates, 35 *Micromonospora* spp. isolates, 3 *Modestobacter* spp. isolates, and 5 *Streptomyces* spp. isolates. The collection also includes one actinomycete genus that is less commonly isolated from environmental samples (*Nonomuraea* spp.; four isolates) as well as between one and five isolates from a variety of additional bacterial genera, including *Actinomadura*, *Bacillus*, *Brevibacillus*, *Kineococcus*, *Kibbella*, *Metabacillus*, *Methylorubrum*, *Microlunatus*, *Nonomuraea*, *Paenibacillus*, *Paractinoplanes*, *Priestia*, *Saccharothrix*, and *Shigella*.

While 16S rRNA comparisons readily predicted the bacterial genus of each isolate, the percent identity with NCBI entries varied at the species level. Thus, we further evaluated the potential for the collection to include novel bacterial species using a 16S rRNA percent identity cut-off value ≤98.65% as a predictor of a new species (15).

Recognizing that low-resolution 16S rRNA sequencing data could artificially predict low sequence identity, the study was restricted to only those isolates with the greatest 16S rRNA sequencing quality to increase the integrity of our analyses, which was defined as ≥90% high quality (Phred score of 40) for 16S rRNA reads lengths greater than 1,300 base pair (16). A total of 91 isolates met these sequence quality requisites. Of these, 80 (87.9%) exhibited ≥98.65% 16S rRNA sequence identity with a known bacterial species (Data File S1). Conversely, 11 (12.1%) Luedemann collection members exhibited less than 98.65% 16S rRNA sequence identity with any known bacterial species within NCBI, suggesting they are newly identified bacterial species. These new putative species include five *Geodermatophilus* isolates (G163, G249, G252, G254, and G263), one *Nonomuraea* isolate (9132A), one *Modestobacter* isolate (G182), one *Streptomyces* isolate (9184C), one *Blastococcus* isolate (G155), and two *Micromonospora* isolates (9165J and 9196D). Indeed, phylogenetic trees comparing the diversity of each of these isolates to reference NCBI 16S rRNA species sequences for each genus indicated appreciable genetic distance, indicative of new species (representative data for the *Geodermatophilus* are provided in Fig. 1B). To verify (or not) that these 11 organisms are novel species, whole-genome sequencing (WGS) was performed and compared to NCBI bacterial genome entries, using an average nucleotide identity (ANI) cut-off value of ≤95% as consensus threshold for a new species designation (17). Quality sequencing data could not be achieved for one isolate, whereas quality genomic sequence was acquired for 10 organisms, 8 of which share less than 95% identity to any publicly available genome, indicating that they are new bacterial species (Data File S1). More directly, G155, G163, and G263 displayed ANI of 90.9%, 92.2%, and 94.2% to *Geodermatophilus siccatus*, respectively, whereas G249 and G252 exhibited 89.7% and 89.7% ANI to *Geodermatophilus bullaregiensis*, and G254 shared 86.1% ANI with *Geodermatophilus daqingensis*, indicating they are novel *Geodermatophilus* species. 9165J is likely to be a novel *Micromonospora* species with an ANI of 89.9% to *Micromonospora costi*, and G182 is a likely new *Modestobacter* species with a closest ANI of only 85.9%.

Because the goal of our study was to evaluate the collection for their ability to produce novel antimicrobial agents, we sought to learn whether members belonging to a known bacterial species exhibit intraspecies diversity as opposed to represent the same strain that was collected multiple times. Accordingly, the 16S rRNA and colony morphologies of the 80 Luedemann collection members belonging to known bacterial species were compared to one another (Data File S2). Comparisons revealed considerable intraspecies variability. More directly, the 16S rRNA of 50 (62.5%) members differed from one another, including all isolates belonging to *Micromonospora cremea* (two isolates), *Geodermatophilus nigrescens* (two isolates), *Geodermatiophilus tziadensis* (two isolates), and *Trujillonella endophytica* (three isolates), suggesting they are not clonal. Likewise, there was also considerable intraspecies 16S rRNA diversity among most isolates belonging to *Geodermatophilus normandii* (12 isolates), *Geodermatophilus obscurus* (six isolates), and *Modestobacter altitudinis* (four isolates). Conversely, a total of 30 (37.5%) collection members appeared to harbor identical 16S rRNA sequences with another entry of the same species, including all members of *Micromonospora eschinospora* (four isolates), *Micromonospora fluostatini* (three isolates), *Micromonospora saelicesensis* (two isolates), *Micromonospora soli* (seven isolates), and *Priesta flexa* (two isolates), as well as the majority of the isolates belonging to *Micromonospora chaiyaphumensis* (two isolates), *Micromonospora soli* (seven isolates), *G. siccatus* (four isolates), and *Blastococcus* (four isolates). However, the colony morphologies of strains sharing identical 16S rRNA sequences were, in most cases, vastly different in color, shape, and size, suggesting that although 16S rRNA sequencing indicates clonality, there are distinct strain-to-strain differences in their genetic composition and/or the expression properties that cannot be measured by 16S rRNA analyses alone (data not shown).

Taken together, comparative genomics of the 16S rRNA sequences of the pilot Luedemann collection subset indicated that it is likely to include at least eight novel bacterial species and appreciable genetic and/or morphological diversity among isolates belonging to previously documented bacterial species. Thus, we set out to determine whether members of the entire collection could produce natural product antimicrobial agents. Of note, as described below, whole-genome sequencing of members of interest that exhibited low 16S rRNA sequence quality and, hence, were not included in the above analysis indicates that the collection includes at least an additional eight novel species.

### Antimicrobial screen

Recognizing that antimicrobial natural products may be temporally expressed in response to growth phase or endogenous and/or exogenous cues, each Luedemann isolate was cultured in nutrient-limited media at four different temperatures and/or growth conditions: (i) 24°C, (ii) 30°C, (iii) 30°C in the presence of heat-killed *A. baumannii*, and (iv) 37°C for a total of 7 days. Microbial supernatants were collected at days 1, 3, and 7 of incubation from each member and individual culture condition and spot plated onto lawns of *A. baumannii*, *S. aureus*, *P. aeruginosa*, *Escherichia coli*, *Mycobacterium abscessus*, *Mycobacterium smegmatis*, and *V. cholerae* to determine if any of the samples displayed antimicrobial properties toward any of those organisms.

**Fig 2:**
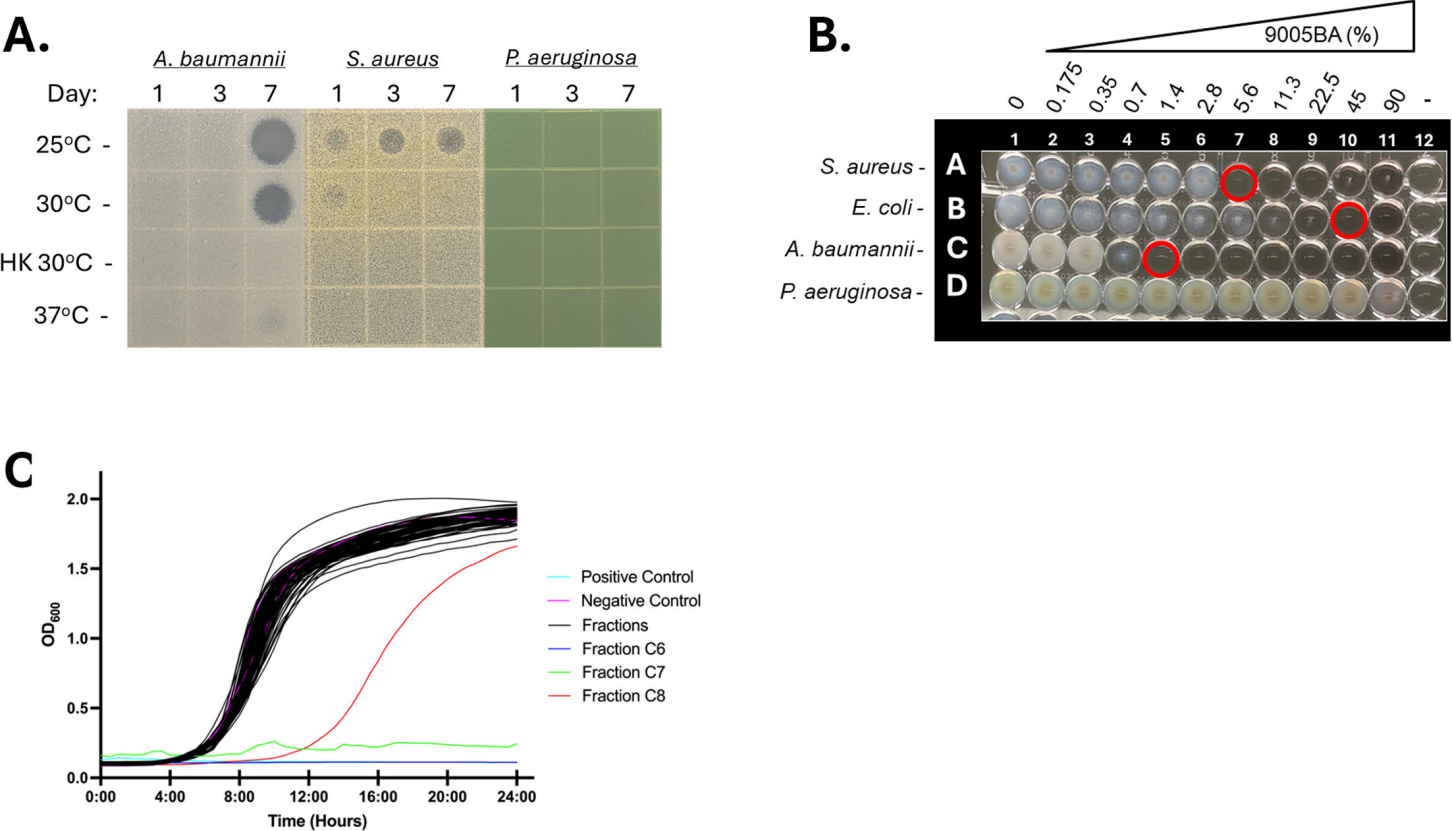
Antimicrobial performance of 9005BA and antibacterial isolation approach. Shown are the antimicrobial properties of “9005BA” supernatants collected after 1, 3, or 7 days growth at 25°C, 30°C, 30°C in the presence of heat-killed (HK) *A. baumannii* strain 98-37-09, or 37°C when applied to agar plates preinoculated with *A. baumannii* (98-37-09), *S. aureus* (UAMS-1), or *P. aeruginosa* (PA01; **A**). Microtiter plate assays to measure the antimicrobial properties of dilutions of 9005BA supernatant toward *S. aureus* (UAMS-1), *E. coli* (TOP10), *A. baumannii* (98-37-09), or *P. aeruginosa* (PA01) cells (**B**). Growth inhibition studies of each fraction (**C**) using *A. baumannii* strain 98-37-09; starting 9005BA supernatant and mock-treated cells shown as positive and negative controls, respectively. See Supplementary Fig. S2A for the microfractionation scheme to generate test fractions.

Representative screening results for Luedemann collection member 9005BA, which features prominently in this work, are provided in Fig. 2A. For 9005BA, only supernatants collected on day 7 of growth at 25°C or 30°C and to a lesser extent 37°C inhibited growth of *A. baumannii*, whereas supernatants collected at any 25°C timepoint or following 1 day growth at 30°C displayed antimicrobial properties toward *S. aureus*. 9005BA supernatants that were collected at other sampling condition(s) failed to affect *A. baumannii* or *S. aureus* growth, and none of the supernatants collected impacted *P. aeruginosa* growth (or other organisms tested; not shown). Interestingly, replicate studies in which 9005BA was grown in nutrient-rich culture conditions failed to detect any antimicrobial activity toward any of the test bacterial species (not shown), suggesting that 9005BA is capable of temporally producing at least one antimicrobial agent during growth in nutrient-limited conditions.

Screening results for the entire collection of 1,764 Luedemann supernatants (147 isolates × four growth conditions × three time points) toward each test organism are provided in Data File S3 and are summarized in Table 1. In addition to 9005BA, supernatant(s) from 12 (8.2%) other Luedemann isolates produced a robust zone of growth inhibition toward at least one test bacterial species. Four isolates produced a zone of inhibition toward a single organism, five produced zones of inhibition toward two organisms, and four produced zones of inhibition toward three bacterial species. Eleven supernatants were active toward *S. aureus* and/or *V. cholerae*, five were active toward *A. baumannii* and/or *E. coli*, and supernatants from three Luedemann isolates were active toward *M. smegmatis*. None of the isolate supernatants displayed antimicrobial activity toward *M. abscessus* or *P. aeruginosa* during these assay conditions.

**TABLE 1:** Luedemann collection screening results

Luedemann isolate	Test organism^*a*^						
	*A. bau*	*E. coli*	*M. abs*	*M. smeg*	*P. aer*	*S. aur*	*V. chol*
9005BA 1-8-94	+	+	−	−	−	+	−
9202F OW 1-21-92	+	+	−	+	−	−	−
9202F W 1-21-92	+	−	−	+	−	−	−
9176P 1-21-92	−	−	−	−	−	+	+
G192 6-19-94	−	−	−	+	−	+	+
9132I 11-23-91	+	−	−	−	−	+	+
9141I 11-23-91	−	−	−	−	−	+	−
M179 6-19-94	−	−	−	−	−	−	+
PAIL-27	±	−	−	−	−	−	+
PAIL-28	−	−	−	−	−	−	+
9027 11-12-91	−	−	−	−	−	+	+
9074 11-6-91	−	−	−	−	−	+	+
GdB 12-21-91	−	−	−	−	−	+	−

^*a*^ *A*. *baumannii* (*A. bau*); *M*. *abscessus* (*M. abs*); *M. smegmatis* (*M. smeg*); *P. aeruginosa* (*P. aer*); *S*. *aureus* (*S. aur*); *V*. *cholerae* (*V. chol*); + indicates antimicrobial; ± indicates inconsistent antimicrobial; − indicates not antimicrobial. The gray shading indicates antibacterial activity of supernatants against each test organism.

### Enriching for organisms that produce novel antimicrobial agents

A caveat of natural product antimicrobial screening programs is the rediscovery of previously identified antibiotics (13). Thus, as an initial step toward enriching for Luedemann isolates that may produce novel antimicrobials, each of the 13 antimicrobial supernatants was re-tested for antimicrobial activity toward antibiotic-resistant strains within our repository. We predicted that if a supernatant displays a loss of antimicrobial performance toward a given antibiotic-resistant strain, then the supernatant’s antimicrobial properties are likely to be (i) modulated by that antibiotic or (ii) inactivated by common antimicrobial resistance determinants and, hence, not therapeutically promising.

For testing, each of the nine supernatants that displayed antimicrobial activity toward *S. aureus* was retested for activity against *S. aureus* strains resistant to streptomycin, erythromycin, rifampicin, tetracycline, fosfomycin, albocycline, or oxacillin. The five supernatants with activity toward *A. baumannii* were retested for growth inhibition toward strains with tetracycline or rifampicin resistance. Representative screening results are shown in Fig. S1A, whereas compiled data for each of the supernatants are shown in Table 2. Screening revealed that supernatants from 9202F OW 1-21-92 lost activity toward tetracycline-resistant *S. aureus*, 9132I 11-23-91 lost activity toward bacterial strains that were resistant to erythromycin, rifampicin, or tetracycline, and 9074 11-6-91 lost activity toward vancomycin-resistant *S. aureus* (VRSA). Thus, we deprioritized these Luedemann collection isolates. Conversely, six Luedemann collection isolates, 9005BA 1-8-04, 9202FW 1-21-92, 9176P 1-21-92, G192 6-19-94, 9027 11-6-91, and GdB 12-21-91, retained antimicrobial activity toward all antibiotic-resistant strains and were prioritized for further testing. Of note, M179 6-19-94, PAIL-27, and PAIL-28 were not evaluated for activity toward antibiotic-resistant *V. cholerae* strains and were also carried forward for further characterization.

**TABLE 2:** Luedemann collection activity toward antibiotic-resistant bacteria

Luedemann isolate	Antibiotic^*a*^							
	ALBO^R^	ERM^R^	FOS^R^	OXA^R^	RIF^R^	STR^R^	VAN^R^	TET^R^
9005BA 1-8-94	+	+	+	+	+	+	+	+
9202F OW 1-21-92	ND	+	+	ND	+	+	ND	−
9202F W 1-21-92	ND	+	+	ND	+	+	ND	+
9176P 1-21-92	+	+	+	+	+	+	+	+
G192 6-19-94	+	+	+	+	+	+	+	+
9132I 11-23-91	+	−	+	+	−	+	+	−
9141I 11-23-91	+	+	+	+	+	+	−	+
M179 6-19-94	ND	ND	ND	ND	ND	ND	ND	ND
PAIL-27	ND	ND	ND	ND	ND	ND	ND	ND
PAIL-28	ND	ND	ND	ND	ND	ND	ND	ND
9027 11-6-91	+	+	+	+	+	+	+	+
9074 11-6-91	+	+	+	+	+	+	−	+
GdB 12-21-91	+	+	+	+	+	+	+	+

^*a*^ Albocycline (ALBO); erythromycin (ERM); fosfomycin (FOS); oxacillin (OXA); rifampicin (RIF); streptomycin (STR); vancomycin (VAN); tetracycline (TET); + indicates antimicrobial; − indicates not antimicrobial; ND indicates not determined. The gray shading indicates antibacterial activity of supernatants against each test organism.

### Whole-genome sequencing

WGS was used as a more definitive means to determine whether the antimicrobial performance of the nine highest priority Luedemann isolates is likely to be mediated by a novel antimicrobial agent and, simultaneously, better define the phylogeny of high-priority isolates. To do so, each organism’s genome was analyzed by antiSMASH to determine whether they carry known natural product antimicrobial biosynthetic gene clusters (BGCs) and by GTDB-Tk to better establish their relatedness to known bacterial species. Data File S4 provides antiSMASH search results for each priority Luedemann isolate.

The genomes of isolates G192 and PAIL-28 do not exhibit similarity to any known natural product antimicrobial BGCs within the antiSMASH database, suggesting the antimicrobial activity detected in the supernatants of these organisms may be due to a previously unappreciated natural product antimicrobial(s). Likewise, while three isolates, PAIL-27, 9027, and 9202FW, were found to harbor genes with similarity to known antimicrobial BGCs, the similarity with these known antibiotic biosynthetic gene clusters is relatively low. Moreover, the antimicrobial properties of PAIL-27, 9027, and 9202FW supernatants do not match the activity of the antibiotics produced by those known biosynthetic gene clusters, suggesting they produce novel antimicrobial agents. More directly, PAIL-27 harbors a gene cluster that shares 80% similarity to the tirandamycin (RNA polymerase inhibitor) biosynthetic cluster, indicating that the organism may be capable of producing a tirandamycin-like molecule (18). However, tirandamycin is active toward gram-positive species (19), whereas PAIL-27 supernatant was only active toward *V. cholerae* and *A. baumannii* (latter inconsistently), both of which are gram-negative organisms (Table 1), suggesting that the antimicrobial within PAIL-27 supernatant is unlikely to be mediated by a tirandamycin-like scaffold. Similarly, 9027 harbors a gene cluster with 84% similarity to the machinery that synthesizes filipin, an antifungal polyene macrolide (20). Yet, the 9027 supernatant(s) collected in these studies do not exhibit antifungal activity (data not shown). Likewise, the genome of 9202FW contains gene clusters with 91% and 81% composition similarity to the gramicidin and tyrocidine biosynthetic gene clusters, respectively. Both are antimicrobial peptides that are known to affect both gram-positive and -negative bacterial species but do not exhibit appreciable activity toward mycobacteria, whereas 9202FW supernatant displays robust anti-mycobacterial activity (Table 1).

Three prioritized Luedemann isolates, 9005BA, 9176P, and M179, harbor gene clusters that show 100% similarity to known antimicrobial BGCs. More specifically, antiSMASH revealed that the 9005BA genome harbors genes with 100% similarity to the Ɛ-poly-L-lysine machinery, an antimicrobial that exhibits antimicrobial activity toward *P. aeruginosa* (21). However, we reasoned that the antimicrobial present in 9005BA supernatants is unlikely to be attributed to Ɛ-poly-L-lysine because our screen did not indicate that 9005BA supernatant exhibits antimicrobial activity toward *P. aeruginosa* (Fig. 2A and Table 1). Indeed, MIC testing using increasing concentrations of 9005BA supernatant confirmed that while it contains agents with antimicrobial efficacy toward *A. baumannii*, *S. aureus*, and *E. coli*, the supernatant does not confer observable activity toward *P. aeruginosa* (Fig. 2B). 9176P harbors genes that are 100% similar to the naringenin and Ɛ-poly-L-lysine biosynthetic gene clusters, respectively. We reasoned that the antimicrobial activity in 9176P supernatants, which is active toward both gram-positive (*S. aureus*) and -negative (*V. cholerae*) bacteria, is unlikely to be attributable to the presence of Ɛ-poly-L-lysine because the agent is highly active toward *P. aeruginosa*, but the supernatant is not. Yet, the organism’s antimicrobial agent(s) could be due to production of naringenin, a trihydroxyflavanone that is known to display antimicrobial activity toward both gram-positive and -negative species. Likewise, M179, which only displayed antimicrobial activity toward *V. cholerae*, contained genes with 100% similarity to the BGC of bicornutin, which displays potent activity toward another gram-negative organism, *Erwinia amylovora* (22). Thus, it is conceivable that the antimicrobial effect of M179 supernatant could be attributable to bicornutin.

Because the 16S rRNA sequence of each of the nine high-priority isolates was below the sequence quality threshold (90% high quality) needed to accurately assess their relatedness to other bacterial sequences, they were not included in the above 16S rRNA analyses. Thus, we also used their whole-genome sequencing results to better assess the phylogeny of each isolate. To do so, the ANI of each isolate was compared to Genome Taxonomy Database entries (596,859 genomes at the time of writing), with an ANI <95% cut-off indicative of a new species (17). This analysis revealed that PAIL27 is likely to be a strain of *Streptomyces radiopugnans* with an average nucleotide identity of 95.0%, whereas the remaining eight high-priority isolates are likely to represent new bacterial species (Data File S1). More specifically, PAIL-28, 9005BA, 9027, 9132I, and 9176P are likely to constitute novel *Streptomyces* species, showing closest genome identities with *Streptomyces taklimakanensis* (90.7% ANI), *Streptomyces mobaraensis* (91.5% ANI), *Streptomyces nondiastaticus* (92.6% ANI), *Streptomyces africanus* (94.5% ANI), and *Streptomyces specialis* (89.3% ANI), respectively. Isolates G192, 9177I, and M179 were found to exhibit the closest genome identity with *Micromonospora purpureochromogenes* (92.8% ANI), *Micromonospora citrea* (92.6% ANI), and *Micromonospora peucetia* (92.6% ANI), suggesting they are likely to be novel *Micromonospora species*.

Taken together, our results suggest that PAIL-28, 9005BA, 9027, 9132I, 9176P, G192, M179, and 9177I are novel bacterial species. Of these, we concluded that the antimicrobial activities of 9176P and M179 supernatants may be attributable to the known natural product antimicrobials naringenin and bicornutin, respectively, whereas the activity of the other isolates is likely to be due to a novel antimicrobial agent. To that end, we observed that 9005BA’s antimicrobial performance correlated directly with the production of a dark purple pigment and considered that this phenotype may expedite defining the organism’s antimicrobial agent.

### Isolation and identification of the antimicrobial agent, compound 1

To chemically define the antimicrobial agent(s) produced by *Streptomyces* sp. strain 9005BA, an overproducer strain, 9005BA+, was selected by individually screening 250 individual colonies for those that produced supernatants with increased antimicrobial activity toward *A. baumannii* following 5 days of growth at 30°C (not shown). 9005BA+ was grown in small scale to generate enough material to allow high pressure liquid chromatography (HPLC) fractionation of 9005BA+ supernatants following the scheme shown in Fig. S2A, with HPLC-DAD (diode array detection) data shown in Fig. S3A. All fractions were evaluated for antimicrobial activity toward *A. baumannii* and, independently, *S. aureus*. While no fractions displayed appreciable activity toward *S. aureus*, two fractions, C6 and C7, eliminated *A. baumannii* growth (Fig. 2C). Mass spectrometry determined that both fractions contained a single *m/z* 227.08 [M + H]^+^ peak that corresponded with potent anti-Acinetobacter activity. To facilitate recovery of a larger quantity of the active agent for further antimicrobial testing and structure elucidation, a scheme for scaled-up antimicrobial isolation was developed (Fig. S2B). The isolated active agent compound 1 represents a 0.05% yield (12.3 mg) of dried 9005BA+ supernatants (24.0 g; Fig. S3B).

**Fig 3:**
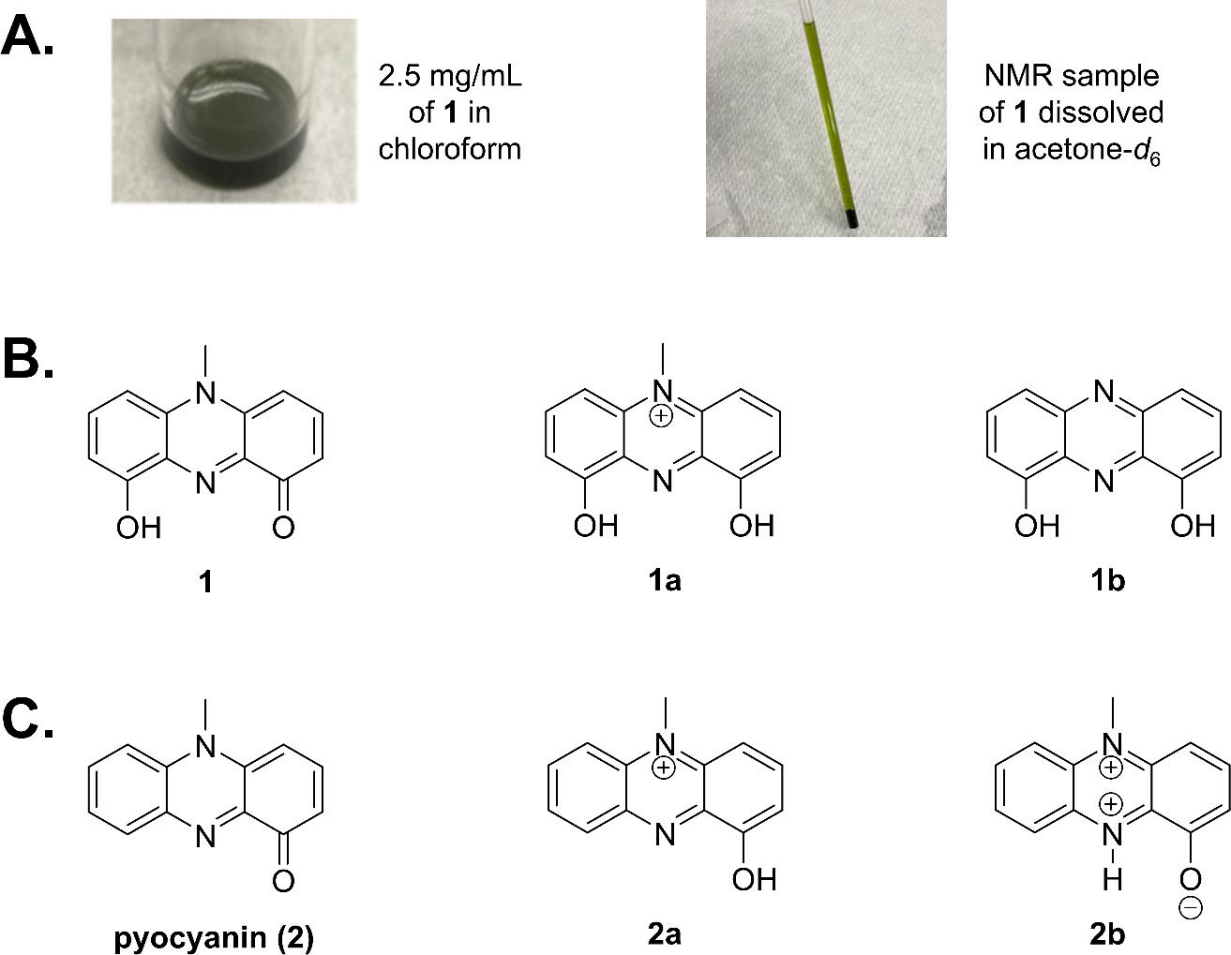
Dark blue-green color of compound 1 (**A**). Chemical structure of compound 1 and its convertible format (1a and 1b; **B**). Chemical structure of pyocyanin (2) and its convertible format (2a and 2b; **C**) (23, 24).

**Fig 4:**
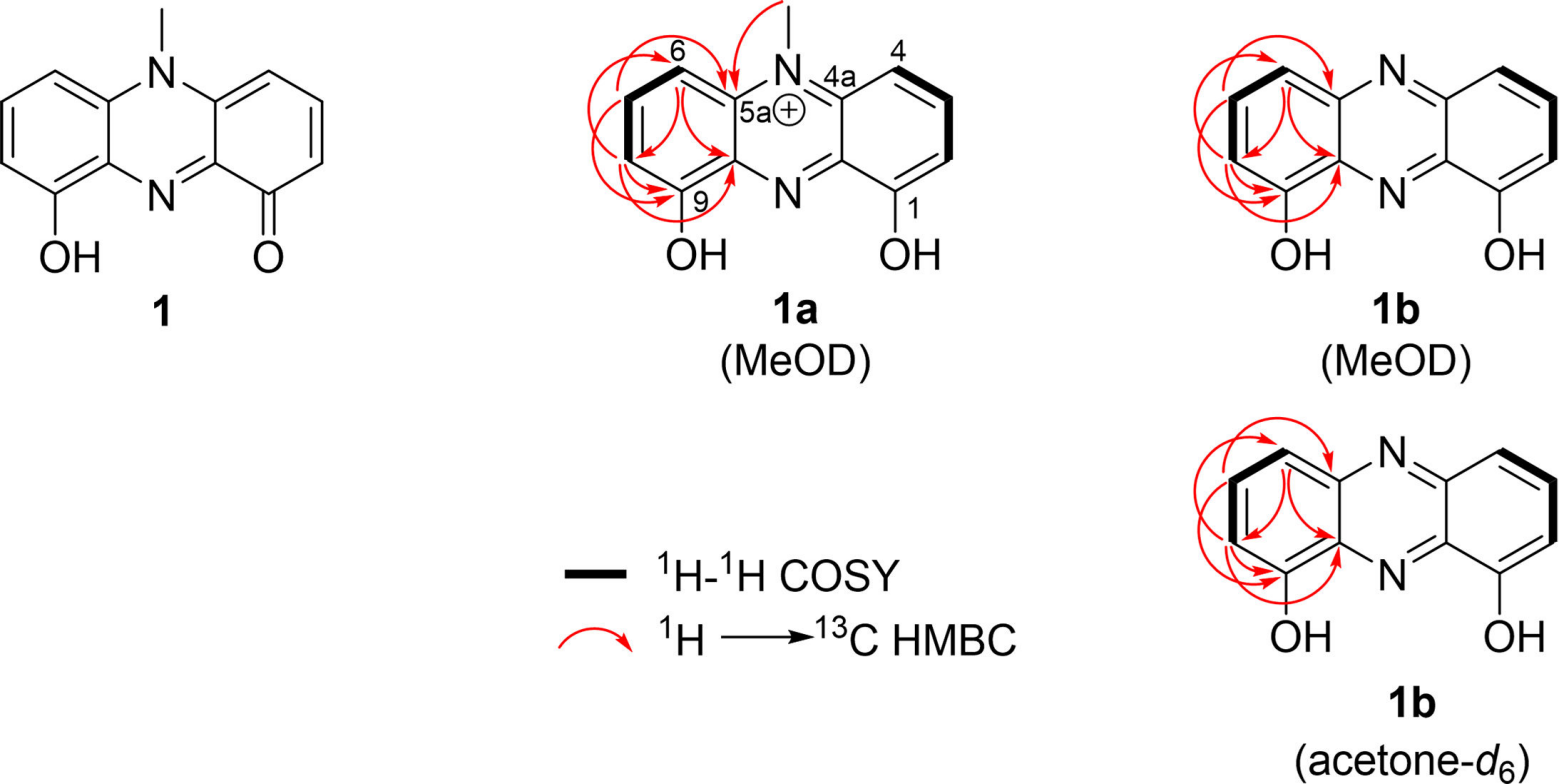
Key ^1^H−^1^H COSY and key HMBC correlations of 1a and 1b.

Compound 1 was isolated as a dark blue pigment (Fig. 3A) and confirmed to exhibit potent activity toward *A. baumannii*. For the structure elucidation of 1, UV-visible spectroscopy, high-resolution electrospray ionization mass spectrometry (HRESIMS), 1D and 2D nuclear magnetic resonance (NMR) spectroscopy, and single X-ray crystallography experiments were performed. The results collectively revealed the molecular formula of 1 is C_13_H_10_N_2_O_2_ by HRESIMS (*m/z* 227.0826 [M + H]^+^, calculated for C_13_H_11_N_2_O_2_, 227.0810), with 10 indices of hydrogen deficiency (Fig. S4A). Its DAD spectrum showed maximum absorption at 271, 318, 371, 458, and 785 nm, suggesting the existence of a long-conjugated system (Fig. S4B). The ^1^H 1D, ^13^C 1D, ^1^H-^1^H correlation spectroscopy (COSY), edited-heteronuclear single quantum coherence (HSQC), and heteronuclear multiple bond correlation (HMBC) spectra revealed that 1 exists as two forms, 1a (positively charged form) and 1b (*N*-demethylated form), in the methanol-*d*_4_ solvent system (Fig. 4 and Fig. S6-10).

More directly, ^1^H NMR (Table 3) indicated that 1a contains two symmetrical 1,2,3-trisubstituted aromatic rings (*δ*_H_ 7.87 [*t*, *J* = 8.4 Hz], 6.89 [*d*, *J* = 8.0 Hz], and 6.75 [*d*, *J* = 8.4 Hz]) and a *N*-methyl proton [*δ*_H_ 4.14, s], and 1b is composed of two symmetrical 1,2,3-trisubstituted aromatic rings (*δ*_H_ 7.78 [*t*, *J* = 8.1 Hz], 7.61 [*d*, *J* = 8.7 Hz], and 7.13 [*d*, *J* = 7.5 Hz]). The ^1^H–^1^H 2D NMR homonuclear COSY spectrum supported these cross-peak correlations in the individual rings of structures 1a and 1b (Fig. 4). Comparing the integral value of protons in the aromatic ring system in 1a and 1b, the ratio of existence was calculated as 73.24% for 1a and 26.76% for 1b.

**TABLE 3:** ^1^H, ^13^C, and HMBC NMR spectroscopic data (800/200 MHz, in methanol-d_4_ and acetone-d_6_)

Position	1a (methanol-d_4_)		1b (methanol-d_4_)		1b (acetone-d_6_)	
	δ_H_	δ_C_	δ_H_	δ_C_	δ_H_	δ_C_
1/9	–^*a*^	170.3	–	156.22	–	154.18
2/8	6.75, d (8.4, 2H)	113	7.13, d (7.5, 2H)	110.63	7.24, dd (8.8, 1.0, 2H)	110.2
3/7	7.87, t (8.4, 2H)	143.52	7.78, t (8.1, 2H)	133.99	7.86, dd (8.8, 7.4, 2H)	133.12
4/6	6.89, d (8.0, 2H)	99.15	7.61, d (8.7, 2H)	118.57	7.74, dd (8.8, 1.0, 2H)	120.35
4a/10a	–	136.27	–	145.15	–	145.39
5-CH_3_	4.14, s (3H)	36.28	–	–	–	–
5a/9a	–	136.95	–	135.41	–	133.85

^*a*^ – indicates no signal detected.

The structures of 1a and 1b were established based on the HMBC experiment. More directly, the observed ^1^H-^1^H COSY and HMBC correlations are marked by bold lines and arrows, respectively, in Fig. 4 for structures 1a and 1b. In 1a, a strong HMBC correlation between H-7 (*δ*_H_ 7.87) and C-9 (*δ*_c_ 170.30) together with a weak correlation between H-8 (*δ*_H_ 6.75) and C-9 (*δ*_c_ 170.30) suggests the location of the carbon at C-9. HMBC correlations from H-8 (*δ*_H_ 6.75) and H-6 (*δ*_H_ 6.89) to C-9a (*δ*_c_ 136.95) revealed that the carbon at C-9a is positioned next to carbon C-9 (*δ*_c_ 170.30). HMBC correlations from H-7 (*δ*_H_ 7.86) and methyl proton at 5-CH_3_ (*δ*_H_ 4.14) to C-5a (*δ*_c_ 136.27) revealed the location of the carbon at C-5a. In 1b, a strong HMBC correlation between H-7 (*δ*_H_ 7.78) and C-9 (*δ*_c_ 156.22) together with a weak correlation between H-8 (*δ*_H_ 7.13) and C-9 (*δ*_c_ 156.22) suggested the location of C-9 bearing a hydroxy group. HMBC correlations from H-8 (*δ*_H_ 7.13) and H-6 (*δ*_H_ 7.61) to C-9a (*δ*_c_ 135.41) revealed that the carbon at C-9a was positioned next to carbon C-9 (*δ*_c_ 156.22). HMBC correlations from H-7 (*δ*_H_ 7.78) to C-5a (*δ*_c_ 145.15) revealed the location of the carbon at C-5a.

The chemical shift of 1a showed high accordance with those for pyocyanin, a blue-pigment *P. aeruginosa* antimicrobial secondary metabolite. To establish that the structures of 1a and 1b are distinct from pyocyanin, the NMR data of pyocyanin were also acquired using methanol-*d*_4_ solvent, as previously described (23). In comparison to pyocyanin, which contains an oxygen on one phenyl ring, 1a is symmetrical, having a hydroxyl group at both phenyl groups (position C-1 and C-9), whereas 1b is the *N*-demethylated form of 1a (see Fig. 3B and C). Parallel NMR studies were performed in acetone solvent. Interestingly, while the 9005BA purified compound did not dissolve well in acetone solvent (Fig. 3A), NMR data collected in acetone-*d*_6_ detected 1b but not the methylated form, 1a (Fig. 4; Fig. S11–S15; Table 3), suggesting that compound demethylation is likely to be a byproduct of the solvent system used.

Based on the above-described NMR data, a neutralized form of 1a was suggested as compound 1, 9-hydroxy-5-methylphenazin-1(*5H*)-one (Fig. 4), with its convertible forms 1a and 1b hypothesized to depend on the pH of the solvent system. To assess whether pH does indeed affect the methylation state of 1, the compound’s mass was assessed by direct injection mass spectrometry under different pH conditions. In a low pH solvent composed of MeOH supplemented with 10% formic acid, 1 (presented by *m/z* 227.0822 [M + H]^+^, 249.0632 [M + Na]^+^, and 453.1553 [2M + H]^+^) was detected, but 1b was not detected (Fig. S5A). Conversely, in a high pH solvent composed of 25% ammonia base solution, two products *m/z* 227.0826 [M + H]^+^ (calcd for C_13_H_11_N_2_O_2_, 227.0810) and *m/z* 213.0654 [M + H]^+^ (calcd for C_12_H_8_N_2_O_2_, 213.0659) were detected, representing 1 and 1b, respectively, confirming that pH influences the methylation state of 1 (Fig. S5B). To evaluate whether 1b can be reversibly changed to the *N*-methylated form, another experiment was performed in which the ammonia base solution containing 1 and 1b was dried and redissolved in MeOH and analyzed by HRMS. Results revealed that 1 (*m/z* 227) was detected, whereas 1b (*m/z* 213) was not, further establishing that the compound’s methylation state is modulated by pH and can reversibly change form from 1b to 1. These structural assignments were also confirmed by single-crystal X-ray diffraction, as described below.

Interestingly, the observed changes in one methylation in the above NMR studies also directly correlated with colorimetric changes of the compound’s suspension, as has been observed for other phenazines. Indeed, the closely related pyocyanin changes from red to blue depending on the solvent environment (23, 24). As noted above, purified 9005BA antimicrobial appears as a green pigment at neutral pH, purple in the ammonia base suspension (1 and 1b), and dark blue in low pH (no detectable 1b; Fig. S5C and D). Taken together and based on its structural relatedness to pyocyanin (2), we have named one as pyocyanin A. Pyocyanin A (1): blue pigment; UV (MeOH) *λ*_max_ (log *ε*) 271 (4.27), 318 (3.54), 371 (3.15), 458 (2.99), and 785 (2.95) nm; ^1^H and ^13^C NMR, see Table 3; HRESIMS *m/z* 227.0826 [M + H]^+^ (calcd for C_13_H_11_N_2_O_2_, 227.0810).

### Single X-ray crystallographic analysis of compound 1

**Fig 5:**
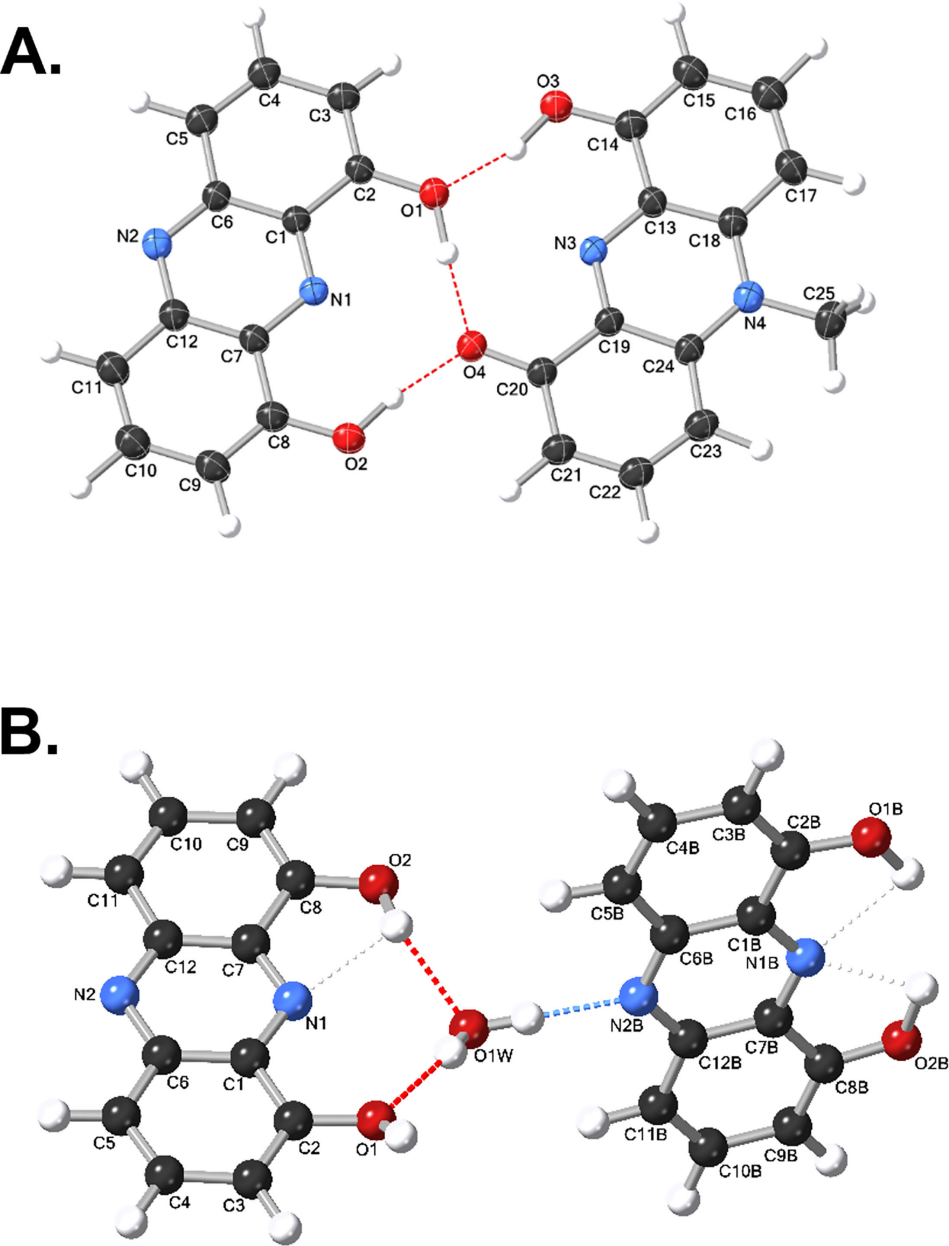
(**A**) X-ray structure of 1 + 1b heterodimer. The formula unit contains a 9-hydroxy-5-methylphenazin-1(*5H*)-one molecule hydrogen bonded to phenazine-1,9-diol molecule (the diol hydrogen atoms donate a pair of hydrogen bonds to the ketone O atom [O4]) and a chloroform molecule of solvation. The chemical formula unit is (C_12_H_8_N_2_O_2_), (C_13_H_10_N_2_O_2_), and (CHCl_3_; the chloroform molecule is not shown here). (**B**) X-ray structure of 1b dimer. The formula unit contains two sets of phenazine-1,9-diol molecules.

Our NMR structural data suggest that pyocyanin A (1) is composed of a reversible mixture of the methylated and the *N*-demethylated form of a novel phenazine compound, whose equilibrium is dependent upon pH, solvent, and concentration. As an additional means to verify this structural assignment, we determined the X-ray crystal structure (Fig. S16–S28 and Tables S1–S14) of the chloroform solvate of pyocyanin A. Analysis of the structure established the existence of two dimers: 1 **+** 1b heterodimers and 1b homodimers, verifying our NMR determinations (Fig. 5). The technical outcomes of crystallographic data of 1 **+** 1b heterodimer include (C_12_H_8_N_2_O_2_), (C_13_H_10_N_2_O_2_), (CHCl_3_), C_26_H_19_Cl_3_N_4_O_4_, dark green needle, 0.281 × 0.042 × 0.02 mm^3^, *M*_*r*_ = 557.823, monoclinic, *P*2_1_/*n* (No. 14), *a* = 7.6650(8) Å, *b* = 10.2857(7) Å, *c* = 30.161(2) Å, *β* = 93.814°(8), α = γ = 90°, *V* = 2372.6(4) Å^3^, *T* = 173.04(16) K, *Z* = 4, *Z*' = 1, μ(Cu K_a_) = 3.874 mm^−1^, 43,737 reflections measured, and 4,307 unique (*R*_int_ = 0.1089), which were used in all calculations. The final *wR*_2_ was 0.1735 (all data), and *R*_1_ was 0.0605 (*I* ≥ 2 σ[*I*]). The technical measures of crystallographic data of the 1b dimer include C_24_H_18_N_4_O_5_, *M_r_* = 442.434, monoclinic, *P*2_1_/*n* (No. 14), *a* = 8.499(6) Å, *b* = 16.173(12) Å, *c* = 14.619(5) Å, *b* = 92.97°(4), *a* = *g* = 90°, *V* = 2,007(2) Å^3^, *T* = 173.01(11) K, *Z* = 4, *Z'* = 1, *m*(Cu K*_a_*) = 0.875, 15,970 reflections measured, 2,893 unique (*R*_int_ = 0.2790), which were used in all calculations. The final *wR*_2_ was 0.3139 (all data), and *R*_1_ was 0.1101 (*I* ≥ 2 *s*[*I*]).

The crystallographic data for compound 1 **+** 1b and 1b dimer have been deposited with the Cambridge Crystallographic Data Center, 12 Union Road, CB2 1EZ, UK (fax: +44-1223-336033; e-mail: deposit@ccdc.cam.ac.uk) and are available on request quoting the deposition number CCDC: 2431860 (compound 1 **+** 1b) and CCDC: 2445872 (1b dimer).

### Pyocyanin A spectrum of activity

**Fig 6:**
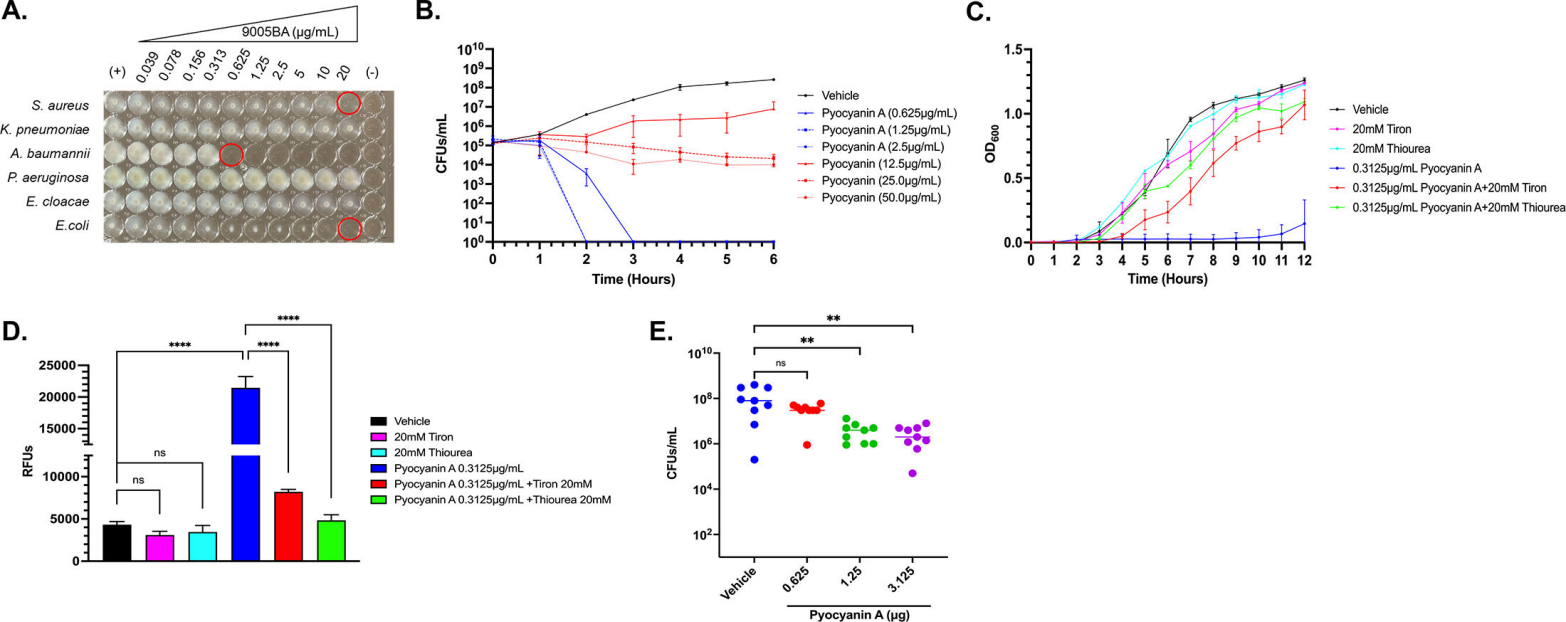
Pyocyanin A (1) antimicrobial characterization. Antimicrobial properties of 0, 0.039, 0.076, 0.156, 0.313, 0.625, 1.25, 2.5, 5, 10, and 20 µg/mL pyocyanin A toward the indicated bacterial species. MIC highlighted in red (**A**). Viable *A. baumannii* following incubation for 0, 1, 2, 3, 4, 5, or 6 hours in the presence of 1×, 2×, and 4× MIC of pyocyanin A or pyocyanin (**B**). Culture density of *A. baumannii* following treatment ±0.5× MIC of pyocyanin A ± 20 mM tiron or thiourea (**C**). Fluorescent measurements of 2',7'-dichlorodihydrofluorescein diacetate-treated cells to measure reactive oxygen species within cultures grown in the absence or presence of 20 mM tiron or thiourea and absence or presence of 0.5× MIC of 1 (**D**). Murine wound *A. baumannii* burden following treatment with vehicle or 1×, 2×, or 5× pyocyanin A; horizontal line indicates average bacterial counts per wound; significant reductions determined by one-way analysis of variance (ANOVA) as ** (*P* ≤ 0.01) or not significant (n.s.) (**E**).

With isolated purified pyocyanin A (1) in hand, we re-evaluated the antimicrobial performance of pyocyanin A in standard MIC testing. As shown in Fig. 6A, purified pyocyanin A exhibited potent antimicrobial activity (0.625 µg/mL; 2.75 µM) toward *A. baumannii* and relatively mild activity toward *S. aureus* or other pathogens evaluated, mimicking the initial spot plating results for 9005BA supernatant (Fig. 2A). The observed selective antimicrobial activity toward *A. baumannii* is in stark contrast to the well-characterized antimicrobial phenazine, pyocyanin (2) (Fig. 3C), which, as noted above, is structurally related, but chemically distinct from pyocyanin A, and displays modest activity toward *S. aureus* and *A. baumannii* (MIC = 6.25 µg/mL; not shown).

To further understand the antimicrobial effects of pyocyanin A, kill curves were performed to assess whether its antibacterial activity was likely to be bactericidal or bacteriostatic. Treatment of *A. baumannii* with 1×, 2×, and 4× MIC of pyocyanin A resulted in a rapid 5-log decrease of bacterial viability within 2–3 hours treatment, establishing that it acts as a bactericidal agent (Fig. 6B). Interestingly, treatment of *A. baumannii* with similar levels (1×, 2×, and 4× MIC) of pyocyanin generated a bacteriostatic effect. Expanded testing of pyocyanin A toward a panel of ten contemporary *A. baumannii* clinical isolates within our repository revealed that it is active toward all strains evaluated (MIC_90_ = 0.4 µg/mL; 1.76 µM) and displays only modest human cell cytotoxicity toward human HEPG2A cells (LD_50_ = 22 µM), resulting in an appreciable therapeutic index ~8 (Fig. S1B).

### Pyocyanin A mechanism of antimicrobial activity

As noted above, pyocyanin A is structurally similar but distinct from pyocyanin, which is a well-studied antimicrobial phenazine that inhibits *S. aureus* cellular respiration and causes oxidative damage by formation of reactive oxygen species (ROS). Given the compound’s structural similarity to pyocyanin, we considered that pyocyanin A may confer antimicrobial activity toward *A. baumannii* in a similar manner by interfering with cellular respiration and/or eliciting oxidative stress.

As a first test of that hypothesis, growth-based rescue experiments were performed in which *A. baumannii* was treated with a sub-inhibitory concentration of pyocyanin A (0.5× MIC; 0.3125 µg/mL), and growth was measured in media alone or media supplemented with 20 mM of the hydroxyl radical scavenger thiourea or the superoxide anion scavenger tiron. While cells treated with 0.5× MIC pyocyanin A showed a severe growth defect, both thiourea and tiron rescued *A. baumannii* growth in the presence of pyocyanin A, suggesting that its antimicrobial effects are mediated, at least in part, by oxidative damage (Fig. 6C). Interestingly, while high concentrations of pyocyanin elicited a bacteriostatic effect on *A. baumannii*, neither thiourea nor tiron significantly rescued *A. baumannii* growth in the presence of 0.5× MIC (3.0 µg/mL) pyocyanin (not shown). As a secondary approach to further probe whether the antimicrobial effects of pyocyanin A correlate with oxidative damage, cellular reactive oxygen species were measured using 2',7'-dichlorodihydrofluorescein diacetate (H_2_DCFDA), a non-fluorescent probe that converts to highly fluorescent 2',7'-dichlorofluorescein (DCF) upon oxidation. These studies revealed that *A. baumannii* showed a fivefold increase in DCF fluorescence following treatment with pyocyanin A, which was either partially or fully reduced to that of untreated cells in the presence of tiron or thiourea, respectively (Fig. 6D). Taken together, these results indicate that the anti-*acinetobacter* effects of pyocyanin A are, at least in part, mediated by the compound’s ability to generate reactive oxygen species within *A. baumannii* cells.

### Pyocyanin A-resistant mutants

To understand *A. baumannii*’s propensity to develop resistance to pyocyanin A and/or corresponding mechanisms of adapting to the compound, cells were stepwise exposed to increasing concentrations of the agent (0.5×, 1×, 2×, and 4× MIC) in liquid culture, and whole-genome sequencing was performed on corresponding pyocyanin A resistant mutants. In duplicate studies, cells were recovered at 2× MIC, but no viable cells were recovered following incubation at 4× MIC, suggesting that high-level *A. baumannii* resistance to the agent is slow to develop.

Comparative genomics revealed that the first mutant identified, 9005BAR.1, harbors three point mutations, corresponding to an Arg275His substitution within the phosphenolpyruvate synthase regulatory protein, PpsR (EAV54_06840), as well as substitutions within a putative TetR/AcR family transcription factor (Leu103Met; EAV54_06565) and a HxlR family helix-turn-helix transcription factor (Cys14Trp; EAV54_09705). The second pyocyanin A-resistant strain, 9005BAR.2, also contained a Leu103Met substitution within EAV54_06565 (TetR/AcrR family transcription factor), as well as amino acid substitutions within the putative multidrug efflux pump repressor AdeL (Pro131Leu; EAV54_06110) and phosphogluconate dehydratase (Ser144Pro; EAVE54_16210). 9005BAR.2 also harbored a frameshift mutation at amino acid position 64 of a putative amidohydrolase (EAV54_04680). Thus, both pyocyanin A-resistant mutants appeared to share the same amino acid substitution within a putative TetR/AcR transcriptional regulator (EAV54_06565) and mutations within gene products involved in cellular respiration. Accordingly, because the TetR family of transcriptional regulators is notorious for affecting antibiotic efflux pumps, we explored whether pyocyanin A resistance correlated with increased efflux capacity and whether the mutants displayed altered cellular respiration by measuring their intracellular ATP levels.

**Fig 7:**
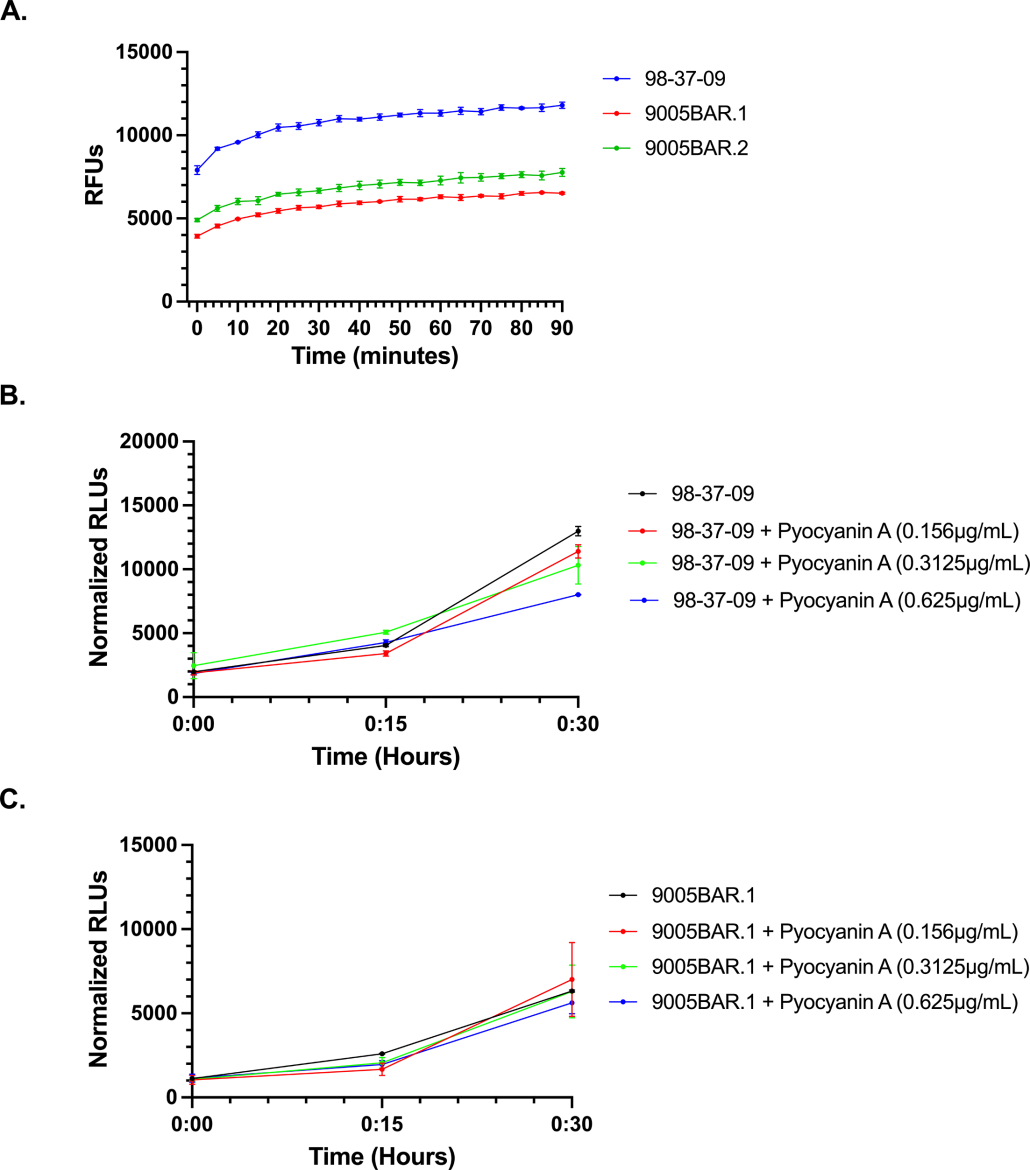
Resistant *A. baumannii* mutants. Plotted are EtBr intracellular accumulation/fluorescence measures of wild-type *A. baumannii* 98-37-09 cells and isogenic resistant mutants, 9005BAR.1 and 9005BAR.2 (**A**). Graphed are intracellular accumulation within wild-type *A. baumannii* (**B**) or 9005BAR.1 (**C**) cells following treatment with 0.156, 0.3125, or 0.625 µg/mL of 1.

Standard ethidium bromide (EtBr) efflux assays, in which the agent is added to exponential phase cells and cellular EtBr accumulation is measured by fluorescence, were performed using wild-type and both pyocyanin A resistant strains (Fig. 7A). Both 9005BAR.1 and 9005BAR.2 exhibited significantly decreased ethidium bromide accumulation in comparison to wild-type 98-37-09 cells, suggesting that both resistant strains exhibit increased efflux activity. Consequently, pyocyanin A resistance could be modulated, in part, by *A. baumannii* drug efflux pumps.

Given that both resistant strains also harbored amino acid substitutions within gene products expected to impact cellular respiration (phosphoenolpyruvate synthase and phosphogluconate), we also compared the ability of wild-type and pyocyanin A-resistant mutants to generate ATP in the absence and presence of 0.25×, 0.5×, and 1× MIC pyocyanin A (wild-type cells). Importantly, it was recognized that because pyocyanin A is a rapidly bactericidal agent, care was taken to establish sublethal treatment conditions (10^6^ cells; 30 min treatment) for these studies. As shown in Fig. 7B, untreated wild-type cells accumulate appreciable levels of ATP over time, whereas pyocyanin A treatment causes a dose-dependent decrease in cellular ATP levels. Studies of 9005BAR.1 (Fig. 7C) revealed a lower basal level of ATP within untreated cells, in comparison to wild-type cells, suggesting that the resistant strain exhibits reduced cellular respiration. Furthermore, pyocyanin A treatment had no significant impact on ATP generation/accumulation. Nearly identical results were observed with 9005BAR.2 (not shown). Taken together, these results indicate that *A. baumannii* may develop resistance to pyocyanin A by two non-mutually exclusive mechanisms: the efflux of the agent and/or reducing cellular respiration.

### Pyocyanin A antimicrobial efficacy in a murine model of *A. baumannii* wound infection

*A. baumannii* is a significant cause of septicemia and difficult-to-treat respiratory and wound infections. As a first test of whether the agent’s antimicrobial effects translate to the infection setting, we evaluated whether pyocyanin A reduces bacterial burden in a murine model of *A. baumannii* wound infection. As shown in Fig. 6E, *A*. *baumannii* strain 98-37-09 readily colonizes mouse wounds. Furthermore, while topical treatment with 1× MIC twice a day for 3 days with pyocyanin A did not significantly impact *A. baumannii* colonization, treatment with 2× (0.625 µg) or 5× (3.1 µg) MIC significantly reduced (p ≤ 0.01) bacterial burden, suggesting that pyocyanin A is effective in reducing wound infections even when dosed infrequently and at low concentrations.

### Historical to modern approach

Building from the Waksman platform of natural product antibiotic drug discovery, in the 1950s, Americo Woyciesjes targeted the collection of *Micromonospora* from soil samples obtained in the Syracuse, NY area (25). Once collected, organisms were grown and isolated in Mr. Woyciesjes’ basement laboratory before being transferred to Dr. George Luedemann at Schering Corporation for sample preparation and screening for natural products that may inhibit cholesterol biosynthesis and, independently, the growth of bacterial pathogens (25). From this rudimentary collection process, *Micromonospora purpurea* was isolated, an organism that produced and allowed the development of gentamicin, one of the predominant antibiotics used today. Following retirement, Dr. Luedemann hypothesized that slow-growing organisms collected from harsh, nutrient-limited environments are likely to produce antimicrobial agents to provide a competitive advantage over faster-growing microbes and, hence, enhance their opportunity for nutrient acquisition and survival. Accordingly, Dr. Luedemann collected and, working in his garage laboratory, subsequently isolated approximately 750 slow-growing microbes from the rock surfaces of desert environments. This collection of organisms, termed the Luedemann collection, has been archived since his passing in 2000. We successfully resuscitated 147 of the first 150 members of the collection, and our work with this pilot isolate set suggests that the Luedemann collection is a rich source of previously undescribed bacterial species and novel natural product antimicrobials.

16S rRNA sequence analysis is recognized as a standard approach to evaluate the relatedness of bacterial isolates. Accordingly, to define the microbial composition of the Luedemann collection, each of the pilot set isolate’s chromosomal DNA was purified, and 16S rRNA was PCR amplified using universal ribosomal RNA primers, sequenced, and BLAST against NCBI bacterial databases. While PCR products were obtained for all members of the collection, reliable sequencing data were not generated for 22 isolates, possibly indicating that these isolates contain mixtures of organisms. Indeed, while every attempt was made to begin working with a single plate-grown colony for our work, many members of the collection appeared to form colonies that seemed to integrate into the calcium carbonate-supplemented agar plate surface, making them difficult to collect and requiring the collection of multiple colonies to subsequently culture in liquid media. We also noted that several isolates appeared homogeneous in morphology during short-term plate incubation but heterogeneous following prolonged (i.e., 14 days) incubation on agar plates, suggesting that they may be mixtures of organisms or undergo some type of phase variation. We did not attempt to distinguish between these two possibilities in this work, although it is notable that isolate 9005BA’s production of pyocyanin A directly corresponded to the organism’s change in color from white to deep purple. We considered that this distinct change in phenotype could be easily followed and could provide an expedited path toward isolating the antimicrobial agent, which is one of the reasons it was selected for further study.

16S rRNA sequencing results revealed that the Luedemann collection is largely composed of bacterial genera typical of environmental samples, with most of the isolates belonging either to the genus *Geodermatophilus* or *Micromonospora*. This was not surprising, given Dr. Luedemann’s experience with both genera. As noted, he discovered the antibiotic gentamicin by screening *Micromonospora* species. Thus, it is presumed that the intent of the collection was to expand isolating and testing the genera for novel antimicrobials. Dr. Luedemann was also the first to discover the Geodermatoderms (26), and we suspect that he viewed the *Geodermatophilus* as an equally valuable source of novel antimicrobials. Our study did not indicate that any of the collection *Geodermatophilus* reproducibly produced significant amounts of an antimicrobial agent within their supernatant; we did observe that *Geodermatophilus sccatus* strain G270 6-23-94 did inconsistently produce antimicrobial activity toward *S. aureus*. Obviously, a caveat to our work is that we have only evaluated the supernatants of collection members that were taken during four growth conditions and at a maximum of 7 days of growth. Other growth conditions or longer incubation times may find that members of the Geodermatoderms produce antimicrobials. Additionally, in initial studies of the first 38 pilot set members that were resuscitated, we found that 12 (32%) displayed contact and/or non-contact inhibition toward members of the ESKAPE pathogens, whereas the supernatants of four (11%) isolates showed activity. Recognizing that the combination of such a high contact/non-contact assay “hit rate” together with the complexity of working with agents that are produced in response to direct contact and/or in close proximity to another bacterium (on agar plates), we refined our study to only isolate supernatants, as described here. It is possible that the Geodermatoderms produce antimicrobials in similar contact or non-contact dependent conditions, and such studies are currently underway in our laboratories.

Despite its power, there are well-known caveats to using 16S rRNA to define the phylogeny of unknown bacteria. Chiefly among these is that the quality of similarity search outcomes is dependent on the integrity of data within search databases and the query sequence(s). Thus, to improve the integrity of our analyses, similarity searches were restricted to 91 isolates with the highest quality sequencing data. A second caveat is the recognition that results can be easily over-interpreted based on defining bacterial similarity based on a single locus and should be considered an approximation. For instance, Rossi-Tamisier and colleagues showed that organisms with identical 16S rRNA sequences can ultimately result in differing species, and the converse has also been shown; organisms with low sequence similarity can ultimately prove to be different strains of the same species (27). The 16S rRNA data obtained in this pilot study and follow-on whole-genome sequencing analyses indicate at least 17 members (12.6%) of the collection are previously undescribed bacterial species. By extension, it is reasonable to predict that the larger 750-member collection includes approximately 95 novel bacterial species.

**Fig 8:**
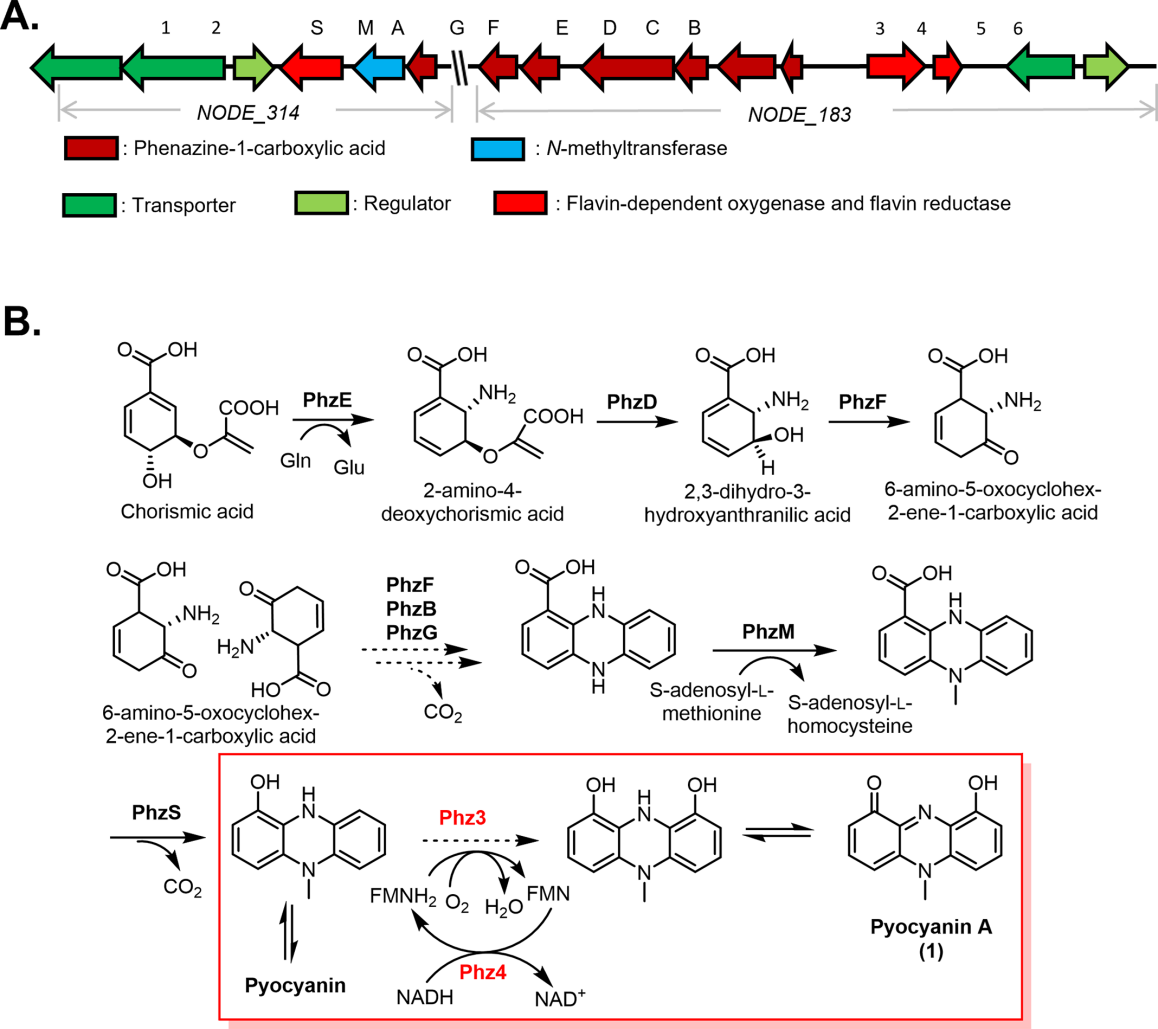
Putative BGC. Proposed BGC (**A**) and biosynthetic pathway (**B**) of pyocyanin A in *Streptomyces* sp. strain 9005BA.

Supernatants of 9 collection members (6.2%) were found to produce an antimicrobial agent(s) and retained activity toward antibiotic-resistant organisms within our laboratory collection. Whole-genome sequencing and antiSMASH indicated that seven of these (77.8%) do not harbor biosynthetic gene clusters required to produce a known natural product antimicrobial that matches the antimicrobial profile of the member’s supernatant. Given the multitude of known antimicrobials within the database, we anticipated that more members of the collection would have been identified to contain known antimicrobial biosynthetic gene clusters. It is worthwhile noting that our antiSMASH analyses are limited to natural product BGCs within the system’s database. To that end, some phenazines are known to exhibit antibacterial activity, but antiSMASH did not predict the phenazine biosynthetic gene cluster within the *Streptomyces* sp. strain 9005BA genome, accentuating the need for continued evolution of the antiSMASH software. To that end, our initial antiSMASH analyses were performed with an earlier version of the software (version 4), whereas the data provided here was generated with the latest iteration (version 7), which has been expanded to identify 81 biosynthetic gene cluster classes. In our experience, version 7 is much improved and allows identification of antimicrobial clusters, such as Ɛ-poly-L-lysine, which were initially missed. We have subsequently used the pyocyanin (phenazine) biosynthetic genes to identify the presumed novel phenazine biosynthetic cluster in 9005BA via BLAST. The putative pyocyanin A BGC (Fig. 8A) consists of two parts located in two nodes of the genome sequence, and its biosynthesis (Fig. 8B) is proposed to follow the pathway similar to pyocyanin (28) based on the presence of serial phenazine biosynthetic gene elements, including *phz*A to *phz*G and *phz*S/M, as determined through bioinformatic analysis. Two additional genes, *phz* 3 and 4, located near phenazine biosynthetic genes, are annotated as flavin-dependent monooxygenase and flavin reductase, respectively, and likely to contribute to pyocyanin A synthesis. Phz3 is proposed to function as a hydroxylase, while Phz4 acts as a coenzyme by providing reduced flavin (29, 30). Our longer-term goal is to test the contributions of these gene products to pyocyanin A production.

The novel phenazine discovered here, 9-hydroxy-5-methylphenazin-1(*5H*)-one, (pyocyanin A), has not been previously described in the literature to our knowledge and is not available within Reaxys or PubChem, supporting Dr. Luedemann’s hypothesis that the collection may be a rich source of novel antimicrobial agents. Pyocyanin A appears to be a mixture composed of the reversibly methylated and *N-demethylated* forms of the compound, the balance of which can be influenced by pH, solvent, and concentration and is differentially distinguished based on color in solvent. For our studies, the antimicrobial properties of pyocyanin A were assessed in media at neutral pH in which the compound appeared deep purple, indicative of the presence of both the methylated (1) and non-methylated (1b) forms, as measured by NMR and crystallography studies. Currently, we are unable to distinguish the individual effects of the two forms on antimicrobial performance, an area of interest for our future work but admittedly complicated by the reversible nature of the methylation properties of the scaffold. Nonetheless, the antimicrobial profile pyocyanin A is distinct from that of pyocyanin (2) and exhibits potent, selective bactericidal activity toward *A. baumannii*. Our results suggest that, like other phenazines, the agent’s mechanism of action is mediated by oxidative damage, as supported by growth rescue studies with ROS scavengers and direct ROS measures. In fitting with that prediction, two pyocyanin A-resistant mutants were isolated, and both were found to harbor single amino acid substitutions within gene products expected to impact aerobic respiration. Consistent with that prediction, both mutants displayed reduced ATP generation and/or accumulation in comparison to wild-type cells, which is expected to limit the generation of endogenous ROS. Both pyocyanin A-resistant mutants also shared a leucine to methionine substitution at amino acid residue 103 within a putative TetR-family transcriptional regulator (TFTR), EAV54_06565. TFTRs are a large family of DNA-binding one-component signal transduction proteins that are commonly associated with antibiotic efflux pump regulation, raising the possibility that pyocyanin A resistance could be attributed to efflux (31). Additionally, each resistant mutant harbored an amino acid substitution in an additional predicted transcriptional regulator; in the case of 9005BAR.2, this included the multidrug efflux regulator AdeL. Corresponding EtBr assays suggested that both pyocyanin A-resistant mutants exhibit increased efflux capacity, in comparison to wild-type cells. Thus, resistance to pyocyanin A could be mediated by increased efflux of the agent from bacterial cells coupled to reduced basal ROS generation.

Collectively, this pilot assessment of the Luedemann collection indicates that it is composed of genetically/morphologically diverse bacteria and includes previously undescribed organisms. Furthermore, focused studies of one of these novel organisms, *Streptomyces* sp. strain 9005BA, have revealed that it produces a novel antimicrobial phenazine. This, combined with the identification of other members that display robust antimicrobial activity yet lack the presence of common antibacterial biosynthetic gene clusters, indicates that the collection is a rich source of novel natural product antimicrobials and supports the Luedemann hypothesis.

## MATERIALS AND METHODS

### Luedemann collection

The Luedemann collection constitutes 750 microbial isolates that were collected from arid, desert rock surfaces from the Southwestern U.S., approximately between the years 1990 and 2000. While records pertaining to the exact location for each sample have not been preserved, the original identifier assigned to each sample has been maintained. All isolates were collected by swabbing rock surfaces directly onto nutrient-limited G-agar (George’s agar; 1% dextrose [D-glucose], 0.5% yeast extract, 0.5% N-Z amine A, 0.1% calcium carbonate, and 2% agar). Plates were incubated at approximately 30°C for 30 days, during which rapidly appearing colonies were killed with a heated inoculation loop to enrich for outgrowth of slow-growing organisms that were archived (−80°C) in 40% glycerol. Collection members were resuscitated by directly streaking onto G-agar and incubating for 30 days at 30°C. For culturing in liquid media, a single colony was used to inoculate 5 mL of G-broth (1% dextrose [D-glucose], 0.5% N-Z amine A, and 0.1% calcium carbonate) in a 16 × 150 mm borosilicate culture tube. The culture was incubated in a rotating drum at 30°C for the indicated duration and processed, as described below.

### 16S rRNA sequencing

16S rRNA sequencing was performed on each Luedemann collection member used in these studies. Each organism was propagated for 30 days in G-broth, and cells were pelleted by centrifugation at 3,000 × *g* for 10 min at room temperature and resuspended in 1 mL of phosphate-buffered saline. Suspensions were transferred to MP Biomedical Blue Capped tubes and mechanically lysed using a FastPrep-24 Bead Beater (MP Biomedicals, Solon, OH). Cell debris was collected by centrifugation, whereas the cell lysate was transferred to Qiagen DNeasy columns for DNA purification following the manufacturer’s recommendations for bacterial DNA isolation (Qiagen, Germantown, MD). A total of 100–200 ng of DNA was PCR amplified using the universal 16S rRNA forward 5’ AGAGTTTGATCCTGGCTCAG and reverse 5’ CGGTTACCTTGTTACGACTT primers, respectively (32). PCR products were purified using QIAquick PCR purification kits (Qiagen) and sequenced by the University of Rochester Genomics Center. Forward and reverse reads were trimmed and assembled using Geneious Prime (version 2024.0.7) and compared to NCBI databases using Basic Local Alignment Search Tool default settings (14). Alignments were carried out in Geneious Prime using MUSCLE 5.1 with high-quality consensus read cut-off values of ≥90%, and phylogenetic placements were performed using Randomized Axelerated Maximum Likelihood version 8.2.11 using GTR GAMMA nucleotide model and bootstrapping set to 1,000 replicates (33–35). Phylogenetic trees were visualized using Tree of Life (iTOL) software with bootstrap values displayed (36).

### Bacterial strains and culture conditions

Laboratory strains used in this study are listed in Table 4. *S. aureus*, *V. cholerae*, and *P. aeruginosa* were propagated in Mueller-Hinton broth (MHB). Albocycline-, fosfomycin-, and rifampicin-resistant derivatives of *S. aureus* strain UAMS-1 were created either by directly plating on MH agar plates supplemented with 8 µg mL^−1^ rifampicin to obtain the rifampicin-resistant strain UAMS-1 R (rifampicin MIC of ≥32 µg/mL) or stepwise cultured with MHB supplemented in 0.5×, 1×, 2×, and 4× MIC of albocycline or fosfomycin to yield strains UAMS-1 AB (albocycline MIC of 16 µg/mL) and UAMS-1 F (fosfomycin MIC of 32 µg/mL), respectively. USA300 is a well-characterized methicillin-resistant *S. aureus* lineage, whereas VRSA1 is a VRSA clinical isolate (37). *E. coli* and *A. baumannii* were grown in Luria-Bertani broth. *A. baumannii* strains 9005BAR.1 and 9005BAR.2 were independently selected by serial passaging strain 98-37-09 in 5 mL Mueller-Hinton media supplemented with increasing concentrations of pyocyanin A containing 9005BA supernatant (0.5×, 1×, 2×, 4×, and 8× MIC) at 37°C for 16 hours. *M. abscessus* and *M. smegmatis* were grown at 37°C in Middlebrook 7H9 broth supplemented with 0.2% glycerol, 1× albumin dextrose and salt solution (ADS: 0.5% bovine serum albumin fraction V, 0.2% dextrose, and 0.85% NaCl), and 0.05% tyloxapol or on Middlebrook 7H10 agar supplemented with the same but omitting tyloxapol.

**TABLE 4:** Bacterial strains used in these studies

Bacteria	Relevant phenotype	Reference/source^*a*^
*S. aureus*		
UAMS-1	Antibiotic-susceptible osteomyelitis isolate	(38)
UAMS-1 AB	Albocycline^R^	This work
UAMS-1 F	Fosfomycin^R^	This work
UAMS-1 R	Rifampicin^R^	This work
VRSA1	Vancomycin^R^	(39)
USA300	Methicillin^R^	(37)
Newman *sbnI*::Tet	Tetracycline^R^	(40)
ISP-479	Erythromycin^R^	A. Bayer
*A. baumannii*		
98-37-09	Antibiotic-susceptible cerebrospinal isolate	(38)
9005BAR.1	Pyocyanin A-resistant 98-37-09 derivative	This work
9005BAR.2	Pyocyanin A-resistant 98-37-09 derivative	This work
98-37-01	Endotracheal tube isolate	(38)
98-37-02	Sputum isolate	(38)
98-37-05	Tracheal aspirate isolate	(38)
98-37-11	Bronchial wash isolate	(38)
ATCC 17904	Urine isolate	ATCC
ATCC 17961	Blood isolate	ATCC
ATCC 17976	Postoperative meningitis isolate	ATCC
ATCC 17978	Fetal meningitis isolate	ATCC
UNMC 4893	Wound isolate	(38)
*E. coli*		
TOP10	Antibiotic-susceptible laboratory strain	Thermo Fisher
*P. aeruginosa*		
PA01	Antibiotic-susceptible laboratory strain	
*V. cholerae*		
AM-19226	TCP/CT-negative, T3SS-positive, O39 serogroup, and streptomycin^R^	(41)
*M. abscessus*		
PM3044	Smooth morphotype clone of ATCC19977 type strain	(42)
*M. smegmatis*		
mc^2^1255	rpsL4 streptomycin^R^ blaS^+^ blaE^+^ β-lactam^R^	(43)

^*a*^ American Type Culture Collection (ATCC).

### Antimicrobial activity assays

In duplicate, each member of the Luedemann collection was incubated in G-broth for 7 days at 30°C and used to inoculate (1:100 dilution) four fresh G-broth tubes that were incubated at either (A) 25°C, (B) 30°C, (C) 30°C in the presence of 10^5^ heat-killed *A. baumannii* strain 98-37-09, or (D) 37°C. Aliquots (0.5 mL) were removed on days 1, 3, and 7 from each incubation condition (A–D), cells were pelleted by centrifugation for 10 min at 3,000 × *g*, and resulting supernatants were sterilized by passage through 0.22 µm filters (Pall Corporation Life Sciences, Port Washington, NY). Each supernatant was spot-plated (20 µL) onto Mueller-Hinton plates pre-seeded with 10^4^–10^5^ of *P. aeruginosa* PA01, *S. aureus* UAMS-1, *E. coli* Top 10, *A. baumannii* 98-37-09, or *V. cholerae* AM-19226, whereas for *M. abscessus* PM3044 and *M. smegmatis* mc^2^1255 supernatants were deposited onto mycobacteria-saturated top agar on 7H10 agar plates. Plates were incubated for 16 hours or 48 hours (mycobacteria) at 37°C, and zones of bacterial growth inhibition were determined by the unaided eye. Where indicated, the antimicrobial activity of supernatants was also measured in triplicate in liquid format, in which individual wells of a 96-well microtiter plate containing various concentrations (0%, 0.35%, 0.70%, 1.41%, 2.81%, 5.63%, 11.25%, 22.5%, 45%, or 90%) of filter-sterilized supernatant was inoculated with 10^4^ CFUs of the indicated bacterial species in MHB. Plates were incubated for 16 hours at 37°C; the lowest concentration of supernatant that inhibited bacterial growth was considered the relative minimal inhibitory concentration. For bacterial kill curve studies, *A. baumannii* 98-37-09 was grown to mid-exponential phase (10^8^ CFUs/mL) and challenged in triplicate with 1×, 2×, or 4× MIC of test agent. Cultures were incubated at 37°C with aeration, and aliquots were taken every hour for 6 hours. CFUs were enumerated, averaged, and compared to mock-treated cells.

### Whole-genome sequencing

Genomic DNA from *A. baumannii* strains 98-37-09, 9005BAR.1, 9005BAR.2, and Luedemann collection members (9005BA 1-8-94, 9202F W 1-21-92, 9176P 1-21-92, G192 6-19-94, PAIL-27, PAIL-28, 9027 11-6-91, G263, G252, G249, G254, G163, 9132A, and 9196D) was sequenced and assembled using Oxford Nanopore Technology long reads at 100× coverage. For *A. baumannii* strains, sequences were uploaded to the Galaxy Web Platform, assembled using Unicycler Version 0.5.1, and were annotated and compared using prokka Version 1.14.6 and Snippy Version 4.6.0 (44, 45). For Luedemann collection members, contigs were queried for relatedness to known microbial species using GTDB-Tk Version 2.3.2 and for known antibiotic-producing loci/gene clusters using antiSMASH Version 7.0.1 (46–48). Loci found to exhibit 100% similarity to known natural product antimicrobial biosynthetic clusters were analyzed for amino acid sequence identity using BLASTP (14).

### Antimicrobial overexpressor strain

An 9005BA hyper antimicrobial producer strain, 9005BA+, was generated by growing 40 individual 9005BA cultures at 30°C for 5 days and testing each for antimicrobial performance against *A. baumannii* in microtiter format, as described above. Cultures that produced the greatest activity following 5 days of growth were plated, and testing was repeated three additional times, at which a stable mutant that consistently generated high antimicrobial activity within 5 days of growth was identified.

### Microfractionation and isolation of an antimicrobial compound from 9005BA supernatant

Culture supernatant from strain 9005BA+ was concentrated *in vacuo* and then lyophilized. Following solvent evaporation, the residual (977.4 mg) was dissolved in MeOH–H_2_O (3:7, [vol/vol]) and loaded onto four Oasis HLB solid-phase extraction (SPE) cartridges (500 mg, 60 µm; Waters, Milford, MA), eluted with 5 mL of MeOH–H_2_O (3:7, [vol/vol]) for each cartridge, to yield fraction 9005BA_30M. Subsequently, fraction 9005BA_100M was washed with 5 mL of 100% MeOH for each cartridge. 9005BA_100M was further micro-fractionated using semi-preparative high pressure liquid chromatography (YMC Hydrosphere C_18_ column, 10 mm × 250 mm, 5 µm; YMC America, Devens, MA) with a compatible guard column at a column, MeCN-H_2_O containing 0.1% formic acid, 5:95→100:0 (vol/vol), 4 mL/min, monitored at 203 nm, 254 nm, 320 nm, and 365 nm) to collect fractions into a 96-well microtiter plate (Fig. S2A).

To facilitate large-scale separation (Fig. S2B), the residual material (24.0 g) was dissolved in MeOH–H_2_O (3:7, [vol/vol]) and loaded onto 4 Oasis HLB SPE cartridges (6 g, 60 µm), eluted with 70 mL of MeOH–H_2_O (3:7, [vol/vol]) for each cartridge, to yield fraction 9005BA_F1. Subsequently, fraction 9005BA_F2 was acquired by eluting MeOH–H_2_O (7:3, [vol/vol]) into the cartridge before a dark blue band washed out. Lastly, fraction 9005BA_F3 was washed out with 100 mL of 100% MeOH for each cartridge. 9005BA_F3 was further separated using semi-preparative HPLC (YMC Hydrosphere C_18_ column, 10 mm × 250 mm, 5 µm, with a compatible guard column at a column; MeCN-H_2_O containing 0.1% formic acid, 5:95→100:0 [vol/vol], 4 mL/min, monitored at 203 nm, 254 nm, 320 nm, and 365 nm) to afford compound 1 (12.1 mg, *t*_*R*_ 18.7 min; Fig. S3A and B).

### General experimental procedures for chemical analysis of compound 1

UV spectrum was recorded on a Chirascan CD spectrometer (Applied Photophysics Ltd., Surrey, UK). 1D and 2D NMR spectra were recorded on a Bruker Avance III HD 800 MHz equipped with a 3 mm TCI CryoProbe (Bruker, Billerica, MA, USA). HRESIMS was performed with an Agilent 6545XT AdvanceBio Q-TOF MS (Agilent Technologies, Inc., Santa Clara, CA, USA), which was equipped with an electrospray ionization (ESI) interface. SPE was performed with Oasis HLB 6 cc Vac Cartridge (500 mg, 60 µm) and Oasis HLB 35 cc Vac Cartridge (6 g, 60 µm; Waters Corporation, Milford, MA, USA). Semi-preparative HPLC separations were performed using an Agilent 1260 system equipped with a photodiode array detector (Agilent Technologies, Santa Clara, CA, USA) by a YMC Hydrosphere C_18_ column (10 mm × 250 mm, 5 µm, YMC CO., Ltd., Kyoto, Japan) with a compatible guard column at a column temperature of 30°C. HPLC data were evaluated by Agilent OpenLab CDS Chemstation edition software. Solvents for SPE fractionation and HPLC were purchased from Sigma-Aldrich (St. Louis, MO, USA). Deuterated solvents for NMR analysis were purchased from Cambridge Isotope Laboratories (Cambridge, MA, USA).

### Liquid chromatography-mass spectrometry analysis

Compound 1 was dissolved in MeOH for liquid chromatography-mass spectrometry analysis on an Agilent 1290 Infinity II UHPLC system coupled to an Agilent 6545XT QTOFMS (Agilent Technologies, Santa Clara, CA, USA), which was equipped with a Dual AJS ESI Ion Source (Agilent Technologies). Chromatographic separations were performed on a Zorbax Eclipse XDB-C18 (100 × 2.1 mm, 1.8 µm; Agilent Technologies) column coupled with Zorbax Eclipse XDB-C18 (5 × 2.1 mm, 1.8 µm) guard column. The mobile phase was composed of H_2_O (A) and acetonitrile (B), both of which were acidified with 0.1% formic acid. The column temperature and sample organizer were maintained at 40°C and 15°C, respectively. A stepwise gradient method at a constant flow rate of 0.4 mL/min was used to elute the column with the following conditions: 5%–5% B (0.0–0.5 min); 5%–25% B (0.5–6.0 min); 25%–100% B (6.0–9.0 min); and 100%–100% B (9.0–10.5 min), followed by a return to the starting conditions at 10.6 min and 1.4 min of reconditioning the column (total runtime of 12.0 min). Analyses of the samples (2.0 µL, injection volume) were performed in the positive ion mode in both profile and centroid mode. The ESI conditions were set as follows: the capillary voltage was 3.5 kV, the nozzle voltage was 200 V for negative mode, the fragmentor was 125 V, the drying gas temperature and flow were set to 150°C and 10 L/min, respectively, and sheath gas temperature and flow were 375°C and 11 L/min, respectively, and the nebulizer was operating at 35 psi. Nitrogen served both as the nebulizer gas and the dry gas. The auto-MS/MS mode was used with an MS range of *m/z* 100–1,700 and an MS2 range of *m/z* 50–1,700, at 7 spectra/s and 5 spectra/s, respectively. The narrow isolation (~1.3 *m/z*) width was used. The collision energy was set by the formula based on the *m/z* and charge of the precursor (condition 1: slope of 4.0 and an offset of 20, condition 2: slope of 4.5 and an offset of 30, condition 3: slope of 5.0 and an offset of 40). The maximum precursors per cycle were set to 5, with the absolute precursor threshold set to 500 (relative threshold 0.015%), and active exclusion was applied after three scans and released after 0.1 min. Direct injection mass spectrometry was also conducted under the same qudrupole time-of-flight (QTOF) conditions with 20 µL/min injection speed. MassHunter Workstation Acquisition B.10.00 software and MassHunter Qualitative Analysis 10.0 software (Agilent Technologies) were used for acquiring and processing MS data.

### Single crystal X-ray diffraction

Single very dark green needle-shaped crystals of compound 1 were recrystallized from chloroform by slow evaporation. A suitable crystal with dimensions 0.28 × 0.04 × 0.02 mm^3^ was selected and mounted on a loop with paratone on an XtaLAB AFC11 (RCD3) quarter-chi single diffractometer (Rigaku, Tokyo). The crystal was kept at a steady *T* = 173(1) K during data collection. The structure was solved with the ShelXT (49) solution program using iterative methods and by using Olex2 1.5-alpha (50) as the graphical interface. The model was refined with olex2.refine 1.5-alpha (51) using full matrix least squares minimization on *F*^2^. There is a single formula unit in the asymmetric unit, which consists of a phenazine-1,9-diol (C_12_H_8_N_2_O_2_) molecule, a 9-hydroxy-5-methylphenazin-1(*5H*)-one molecule (C_13_H_10_N_2_O_2_) and a chloroform molecule. *Z* is 4, and *Z*' is 1. All atoms, even hydrogens, were refined anisotropically. Due to the role of the hydrogens in the intermolecular bonding, these hydrogen atoms were located from the electron density and freely refined using Hirshfeld scattering factors (instead of placing them in geometrically optimal positions).

### ROS studies

In duplicate, 10^5^ mid-exponential phase, *A. baumannii* 98-37-09 cells were transferred to fresh MHB supplemented with 0.5× MIC pyocyanin A (0.3125 µg/mL) or 0.5× pyocyanin (6.25 µg/mL) in the absence or presence of 20 mM tiron (Thermo Fisher Scientific, Waltham, MA) or thiourea (Thermo Fisher) and incubated at 37°C with aeration. The OD_600_ of each sample was measured in triplicate hourly to monitor bacterial growth for a total of 24 hours in a Biotek Synergy H1 Multimode Reader (Biotek, Winooski, VT). To measure intracellular ROS accumulation, growth conditions were repeated (as above) in duplicate for a total of 7 hours, cells were pelleted by centrifugation and resuspended in phosphate buffer solution and split into two aliquots. One aliquot was plated to enumerate CFU per milliliter. The second aliquot was divided into three samples, and a total of 2 µL of 10 mM H_2_DCFDA (Thermo Fisher Scientific) was added to 98 µL of each of the three suspensions and incubated for 1 hour at 37°C. Fluorescence was measured on a Biotek Synergy H1 Multi-mode Reader at excitation/emission wavelengths of 492/527 nm and normalized to 1 × 10^7^ CFUs and averaged.

### Mammalian cell cytotoxicity

Standard 3-(4,5-dimethylthiazol-2-yl)−2,5-diphenyltetrazolium bromide (MTT) colorimetric assays were performed using human liver epithelial cells (HEPG2) challenged with the indicated agent(s) and 12 mM MTT reagent (Cyquant Cell Viability kit; Invitrogen, Carlsbad, CA, USA). Cell viability was measured using a SPECTRAmax5 microplate reader (Molecular Devices, Sunnyvale, CA, USA) as absorbance at OD_540 nm_. All compounds were tested in triplicate, and cell viability was expressed as a percent viability of treated cells in comparison to mock-treated cells; mitomycin C (125 µg/mL) was used as a positive control.

### Ethidium bromide efflux assays

EtBr efflux assays were performed to assess the efflux capacity of *A. baumannii* 98-37-09, 9005BAR.1, and 9005BAR.2 cells, as previously described (52). Briefly, strains were grown to mid-exponential phase at 37°C with aeration, collected by centrifugation at 900 × *g* for 20 min at 4°C, washed three times with sodium phosphate buffer, and resuspended to a final OD_600 nm_ of 0.2 in fresh buffer. A total of 1 × 10^6^ CFUs were transferred to individual wells of a 96-well white plate (Falcon, Corning Life Sciences) treated with 10 µg/mL EtBr, and fluorescence was monitored (excitation, 530_nm_; emission, 600_nm_) every 5 min for a total of 90 min on a Biotek Synergy H1 Multi-mode Reader. All experiments were performed at least three times, and results were averaged and presented with ±SD.

### ATP respiration

In triplicate, *A. baumannii* 98-37-09, 9005BAR.1, and 9005BAR.2 were grown to mid-exponential phase and diluted to a final concentration of 10^6^ CFUs/mL in fresh MHB. Cells were treated with vehicle, and 0.5× (0.3125 µg/mL) or 1× (0.625 µg/mL) MIC of pyocyanin A, and aliquots (60 µL) were collected at 0, 15, 30, and 45 min. For each aliquot, 10 µL was plated for CFU enumeration, and the remaining volume was lysed with 2 mg/mL lysozyme at 37°C for 30 min, and ATP concentrations were determined by adding 50 µL of Promega (Madison, WI) CellTiter-Glo 2.0 reagent and measuring luminescence in a Biotek Synergy H1 Multi-mode Reader. Relative fluorescent units were normalized to an equivalent number of cells.

### *A. baumannii* murine wound infection model

Four- to six-week-old female BALB/c mice (Charles River Laboratories; Washington, MA) were processed following the University of Rochester Medical Center Institutional Animal Care and Use Committee (University Committee on Animal Resources; UCAR) protocol UCAR 2017-022. Mice were anesthetized by intraperitoneal injection with a mixture of 100 mg/kg ketamine (Hikma Berkley Heights, NJ) and 20 mg/mL xylazine (Akorn Inc, IL) followed by subcutaneous administration of 2.5 mg/mL bupivacaine (Hospira Inc, IL). Wounds were created on the dorsal midsection with a 6 mm biopsy punch (Acuderm Inc, FL) and inoculated with 1 × 10^7^ *A. baumannii* 98-37-09. Groups of mice (*n* = 9) were topically treated at the wound site with 10 µL vehicle (sterile water), 1× MIC, 2× MIC, or 5× MIC of pyocyanin A at 45 min post-infection and 12-hour intervals for 3 days. Animals were euthanized, and wound tissues were homogenized, serially diluted, and plated to enumerate bacterial load.

## Data Availability

The genomic sequences for the following Luedemann collection members have been deposited to NCBI under the accession numbers provided in parentheses: isolate 9005BA (JBPQTJ000000000.1), isolate 9027 (JBPQTW000000000.1), isolate 9074 (JBPQTV000000000.1), isolate 9125C (JBPQTU000000000.1), isolate 9132I (JBPQTT000000000.1), isolate 9141I (JBPQTS000000000.1), isolate 9165J (JBPQTR000000000.1), isolate 9176P (JBPQTQ000000000.1), isolate 9177I (JBPQTP000000000.1), isolate 9196D (JBPQTO000000000.1), isolate 9202FOW (JBPQTN000000000.1), isolate 9202FW (JBPQTM000000000.1), isolate G192 (JBPQTL000000000.1), isolate M179 (JBPQTK000000000.1), isolate PAIL27 (JBPQTJ000000000.1), and isolate PAIL28 (JBPQTI000000000.1).

## SUPPLEMENTAL MATERIAL

Data File S1 msphere.00367-25-s0001.xlsx Composition of the Luedemann collection.

Data File S2 msphere.00367-25-s0002.xlsx Intraspecies diversity.

Data File S3 msphere.00367-25-s0003.xlsx Antimicrobial screening results.

Data File S4 msphere.00367-25-s0004.xlsx antiSMASH predictions.

Supplemental Tables and Figures msphere.00367-25-s0005.docx Tables S1 to S14 and Figures S1 to S28.

ASM does not own the copyrights to Supplemental Material that may be linked to, or accessed through, an article. The authors have granted ASM a non-exclusive, world-wide license to publish the Supplemental Material files. Please contact the corresponding author directly for reuse.
