# Supplementary material for: Testing the Luedemann hypothesis: the discovery of novel antimicrobials from slow-growing microbes from nutrient-limited environments
Source: mSphere. 2025 Sep 23;10(10):e00367-25. doi: 10.1128/msphere.00367-25 (PMC12570475; doi:10.1128/msphere.00367-25)
Supplement: Supplemental Tables and Figures — Tables S1 to S14 and Figures S1 to S28. [file msphere.00367-25-s0005.docx]

**SUPPLEMENTAL DATA SECTION**

**SUPPLEMENTAL DATA FILES**

**Supplemental Table SD1.** Microbial Composition of the Luedemann Collection. (Excel attachment)

**Supplemental Table SD2.** Intraspecies 16S rRNA Pairwise Analyses. (Excel attachment)

**Supplemental Table SD3.** Supernatant screening results. (Excel attachment)

**Supplemental Table SD4.** AntiSMASH search results for each of the priority Luedemann isolates. (Excel attachment)

**SUPPLEMENTAL TABLES**

**Supplemental Table ST1**: Fractional Atomic Coordinates (×10^4^) and Equivalent Isotropic Displacement Parameters (Å^2^×10^3^) for **1+1b** heterodimer. *U_eq_* is defined as 1/3 of the trace of the orthogonalised *U_ij_*..

| **Atom** | **x** | **y** | **z** | ***U_eq_*** |
| --- | --- | --- | --- | --- |
| Cl1 | -856(2) | -976.0(12) | 2647.7(5) | 101.3(5) |
| Cl2 | -2127.0(17) | 1235.9(12) | 3111.1(4) | 79.0(4) |
| Cl3 | -1665.0(17) | 1381.5(15) | 2175.7(4) | 91.2(5) |
| C1s | -887(7) | 724(4) | 2678.2(14) | 61.5(12) |
| O1 | 6357(3) | 2307(2) | 4427.6(8) | 50.0(7) |
| O2 | 4219(3) | 6184(2) | 3810.2(8) | 53.7(7) |
| N1 | 4416(4) | 3555(2) | 3774.4(9) | 41.7(7) |
| N2 | 2475(4) | 2157(3) | 3114.4(10) | 48.3(8) |
| C1 | 4427(5) | 2261(3) | 3772.1(11) | 40.7(8) |
| C2 | 5431(5) | 1603(3) | 4118.7(12) | 44.8(9) |
| C3 | 5420(6) | 280(3) | 4131.8(14) | 53.5(10) |
| C4 | 4424(6) | -444(4) | 3801.3(13) | 57.6(11) |
| C5 | 3455(5) | 154(4) | 3461.9(13) | 54.6(10) |
| C6 | 3425(5) | 1540(3) | 3439.4(12) | 45.0(9) |
| C7 | 3423(5) | 4161(3) | 3460.0(11) | 42.4(8) |
| C8 | 3324(5) | 5543(3) | 3476.6(12) | 47.5(9) |
| C9 | 2328(5) | 6183(4) | 3157.0(13) | 54.4(10) |
| C10 | 1403(5) | 5487(4) | 2806.9(13) | 55.1(10) |
| C11 | 1439(5) | 4160(4) | 2783.3(13) | 52.0(9) |
| C12 | 2457(5) | 3456(3) | 3116.6(12) | 45.3(8) |
| O3 | 8001(4) | 1122(2) | 5139.6(9) | 60.5(8) |
| O4 | 6004(3) | 4962(2) | 4506.6(7) | 46.6(6) |
| N3 | 7945(4) | 3750(2) | 5163.8(9) | 39.7(7) |
| N4 | 9860(4) | 5128(3) | 5821.1(9) | 44.5(7) |
| C22 | 7600(5) | 7713(4) | 5152.2(13) | 51.6(9) |
| C13 | 8930(5) | 3133(3) | 5478.8(11) | 42.8(8) |
| C14 | 8943(5) | 1738(3) | 5458.6(12) | 50.3(9) |
| C15 | 9963(6) | 1055(4) | 5775.0(13) | 60.5(11) |
| C16 | 10966(6) | 1733(4) | 6104.5(14) | 59.2(10) |
| C17 | 10985(5) | 3078(4) | 6130.4(13) | 55.1(10) |
| C18 | 9948(5) | 3795(3) | 5819.5(11) | 43.7(8) |
| C19 | 7877(5) | 5028(3) | 5168.3(11) | 40.0(8) |
| C20 | 6781(5) | 5651(3) | 4812.2(11) | 42.5(8) |
| C21 | 6661(5) | 7019(3) | 4824.6(13) | 50.0(9) |
| C23 | 8695(5) | 7144(3) | 5489.1(13) | 50.5(9) |
| C24 | 8838(5) | 5800(3) | 5504.6(11) | 42.5(8) |
| C25 | 10882(5) | 5840(4) | 6166.1(12) | 58.1(11) |

**Supplemental Table ST2**: Anisotropic Displacement Parameters (×10^4^) for **1+1b** heterodimer. The anisotropic displacement factor exponent takes the form: *-2p^2^[h^2^a*^2^ × U_11_+ ... +2hka* × b* × U_12_].*

| **Atom** | ***U_11_*** | ***U_22_*** | ***U_33_*** | ***U_23_*** | ***U_13_*** | ***U_12_*** |
| --- | --- | --- | --- | --- | --- | --- |
| Cl1 | 117.3(12) | 61.3(7) | 126.0(12) | -8.6(7) | 14.5(9) | -21.1(7) |
| Cl2 | 88.9(9) | 81.0(8) | 67.6(7) | 1.9(7) | 9.1(6) | -8.0(6) |
| Cl3 | 75.7(9) | 134.2(12) | 61.3(8) | 18.9(8) | -13.4(6) | 2.5(7) |
| C1s | 57(3) | 64(3) | 61(3) | -3(2) | -9(2) | -2(2) |
| H1s | 56(5) | 40(15) | 68(17) | 4(3) | -10(4) | -2(8) |
| O1 | 61.5(17) | 36.7(12) | 49.2(13) | 0.0(9) | -15.9(8) | 0.0(7) |
| H1 | 60(20) | 37(3) | 20(17) | -2(2) | 1(10) | 4(2) |
| O2 | 67.4(18) | 33.1(12) | 58.0(14) | 2.5(9) | -15.8(9) | 3.2(7) |
| N1 | 47.6(16) | 30.9(8) | 45.0(13) | 0.3(6) | -9.1(8) | -0.1(5) |
| N2 | 51.6(17) | 41.6(9) | 49.7(14) | 0.1(6) | -11.6(8) | -1.1(5) |
| C1 | 45.9(18) | 30.8(8) | 43.8(13) | 0.3(6) | -9.0(8) | -0.1(5) |
| C2 | 52.6(19) | 33.7(10) | 46.1(14) | 3.0(7) | -11.9(8) | 0.1(5) |
| C3 | 65(2) | 33.5(10) | 59.7(19) | 2.7(7) | -15.4(12) | 0.5(6) |
| H3a | 120(30) | 40(9) | 82(12) | 0(5) | -48(9) | 6(3) |
| C4 | 68(2) | 38.7(12) | 63.1(18) | 0.9(8) | -16.7(12) | -2.5(6) |
| H4 | 240(50) | 40(3) | 150(30) | -13(3) | -124(18) | 8(2) |
| C5 | 64(2) | 36.4(9) | 61.4(19) | -0.9(6) | -14.6(12) | -2.9(5) |
| H5 | 140(30) | 46(9) | 100(16) | -3(5) | -67(11) | -8(4) |
| C6 | 49.2(19) | 36.4(9) | 47.6(14) | -1.0(6) | -10.7(8) | -3.6(5) |
| C7 | 45.5(17) | 35.5(9) | 44.8(13) | 1.3(6) | -6.8(9) | 2.4(5) |
| C8 | 53(2) | 35.5(9) | 52.3(15) | 1.7(6) | -6.4(9) | 2.2(5) |
| C9 | 58(2) | 46.3(14) | 57.3(16) | 4.7(8) | -8.1(10) | 7.6(7) |
| H9 | 70(20) | 46(3) | 67(15) | 6.6(19) | -22(9) | 6.0(16) |
| C10 | 55(2) | 52.2(12) | 56.9(18) | 2.6(9) | -4.6(10) | 4.9(7) |
| H10 | 100(20) | 63(10) | 77(13) | 8(5) | -30(8) | 9(4) |
| C11 | 50(2) | 52.2(12) | 51.9(17) | 2.7(8) | -7.4(9) | 5.0(7) |
| H11 | 90(20) | 59(9) | 83(16) | 0(4) | -40(9) | 2(4) |
| C12 | 46.3(18) | 41.6(9) | 46.7(14) | -0.0(6) | -6.3(9) | -1.0(5) |
| H2 | 28(14) | 32(9) | 39(8) | 6(4) | 11(4) | 0(3) |
| O3 | 80.7(19) | 32.0(12) | 64.8(15) | 1.0(9) | -25.5(10) | 2.8(7) |
| H3 | 70(30) | 37(10) | 62(13) | 4(6) | -18(9) | 4(4) |
| O4 | 55.0(16) | 36.7(12) | 46.5(12) | 0.5(9) | -9.1(8) | -2.6(7) |
| N3 | 47.4(16) | 30.9(8) | 39.4(13) | -1.6(6) | -7.7(8) | -1.0(5) |
| N4 | 49.3(16) | 41.0(9) | 42.4(12) | -1.0(6) | -2.6(7) | -1.3(5) |
| C22 | 64(2) | 37.4(12) | 52.8(17) | -1.7(8) | -1.4(11) | -1.7(6) |
| H22 | 120(30) | 38(3) | 95(19) | 1.6(16) | -46(12) | -4.1(15) |
| C13 | 49.5(18) | 35.9(9) | 41.5(13) | -0.2(6) | -8.3(9) | 1.5(5) |
| C14 | 59(2) | 35.9(9) | 54.1(16) | -0.1(6) | -9.3(10) | 1.1(5) |
| C15 | 70(2) | 48.4(14) | 60.5(17) | 6.4(8) | -13.2(11) | 5.8(7) |
| H15 | 120(30) | 48(3) | 100(20) | 9.3(17) | -53(13) | 3.1(15) |
| C16 | 65(2) | 53.3(12) | 58(2) | 4.1(8) | -7.7(11) | 4.0(7) |
| H16 | 120(30) | 61(9) | 89(18) | 16(4) | -49(10) | -1(4) |
| C17 | 59(2) | 53.4(12) | 50.9(17) | 4.4(8) | -11.3(10) | 4.3(7) |
| H17 | 170(30) | 61(10) | 140(20) | 1(5) | -107(14) | 1(4) |
| C18 | 47.2(18) | 41.0(9) | 41.8(14) | -1.1(6) | -6.0(8) | -1.4(5) |
| C19 | 47.8(17) | 30.9(8) | 40.3(13) | -1.7(6) | -4.2(8) | -1.0(5) |
| C20 | 50.4(18) | 33.9(10) | 42.0(13) | -0.7(6) | -5.4(8) | 0.2(5) |
| C21 | 63(2) | 33.8(10) | 52.0(18) | 0.0(6) | -2.2(11) | 0.0(5) |
| H21 | 120(20) | 39(9) | 80(15) | 8(4) | -40(9) | -3(4) |
| C23 | 64(2) | 35.3(9) | 51.9(18) | -2.0(6) | -1.4(11) | -3.1(5) |
| H23 | 130(30) | 42(8) | 95(18) | 0(4) | -49(11) | -10(4) |
| C24 | 49.2(18) | 35.2(9) | 42.1(13) | -3.2(6) | -4.1(8) | -3.6(5) |
| C25 | 63(3) | 57(2) | 52.5(19) | -6.5(12) | -8.5(11) | -9.4(10) |
| H25a | 77(10) | 60(3) | 65(11) | -2(2) | -6(4) | -10(2) |
| H25b | 77(11) | 68(9) | 55(4) | -9(4) | -8(2) | -5(2) |
| H25c | 65(4) | 69(11) | 60(10) | -8(2) | -7(2) | -14(4) |

**Supplemental Table ST3**: Bond Lengths in Å for **1+1b** heterodimer.

| **Atom** | **Atom** | **Length/Å** | |
| --- | --- | --- | --- |
| Cl1 | C1s | 1.752(4) |  |
| Cl2 | C1s | 1.746(5) |  |
| Cl3 | C1s | 1.729(4) |  |
| C1s | H1s | 1.09(4) |  |
| O1 | H1 | 1.0698(18) |  |
| O1 | C2 | 1.345(4) |  |
| O2 | C8 | 1.351(4) |  |
| O2 | H2 | 1.0698(18) |  |
| N1 | C1 | 1.331(4) |  |
| N1 | C7 | 1.330(4) |  |
| N2 | C6 | 1.341(4) |  |
| N2 | C12 | 1.337(4) |  |
| C1 | C2 | 1.427(5) |  |
| C1 | C6 | 1.430(5) |  |
| C2 | C3 | 1.362(5) |  |
| C3 | H3a | 1.02(4) |  |
| C3 | C4 | 1.425(5) |  |
| C4 | H4 | 1.09(4) |  |
| C4 | C5 | 1.370(5) |  |
| C5 | H5 | 1.12(4) |  |
| C5 | C6 | 1.427(5) |  |
| C7 | C8 | 1.425(5) |  |
| C7 | C12 | 1.430(5) |  |
| C8 | C9 | 1.359(5) |  |
| C9 | H9 | 1.09(3) |  |
| C9 | C10 | 1.425(5) |  |
| C10 | H10 | 1.22(4) |  |
| C10 | C11 | 1.366(5) |  |
| C11 | H11 | 1.12(4) |  |
| C11 | C12 | 1.428(5) |  |
| O3 | H3 | 1.0698(18) |  |
| O3 | C14 | 1.325(4) |  |
| O4 | C20 | 1.278(4) |  |
| N3 | C13 | 1.334(4) |  |
| N3 | C19 | 1.316(4) |  |
| N4 | C18 | 1.374(4) |  |
| N4 | C24 | 1.380(4) |  |
| N4 | C25 | 1.458(4) |  |
| C22 | H22 | 1.08(3) |  |
| C22 | C21 | 1.382(5) |  |
| C22 | C23 | 1.402(5) |  |
| C13 | C14 | 1.436(5) |  |
| C13 | C18 | 1.421(5) |  |
| C14 | C15 | 1.385(5) |  |
| C15 | H15 | 1.12(4) |  |
| C15 | C16 | 1.401(6) |  |
| C16 | H16 | 1.14(4) |  |
| C16 | C17 | 1.385(6) |  |
| C17 | H17 | 1.12(4) |  |
| C17 | C18 | 1.399(5) |  |
| C19 | C20 | 1.466(5) |  |
| C19 | C24 | 1.450(5) |  |
| C20 | C21 | 1.411(5) |  |
| C21 | H21 | 1.11(4) |  |
| C23 | H23 | 1.11(4) |  |
| C23 | C24 | 1.387(5) |  |
| C25 | H25a | 1.047(13) |  |
| C25 | H25b | 1.047(13) |  |
| C25 | H25c | 1.047(13) |  |

**Supplemental Table ST4**: Bond Angles in ^°^ for **1+1b** heterodimer.

| **Atom** | **Atom** | **Atom** | **Angle/^°^** | |
| --- | --- | --- | --- | --- |
| Cl2 | C1s | Cl1 | 110.5(3) |  |
| Cl3 | C1s | Cl1 | 110.4(2) |  |
| Cl3 | C1s | Cl2 | 111.4(3) |  |
| H1s | C1s | Cl1 | 106.9(19) |  |
| H1s | C1s | Cl2 | 110(2) |  |
| H1s | C1s | Cl3 | 108(2) |  |
| C2 | O1 | H1 | 121(2) |  |
| H2 | O2 | C8 | 117.0(19) |  |
| C7 | N1 | C1 | 117.9(3) |  |
| C12 | N2 | C6 | 118.3(3) |  |
| C2 | C1 | N1 | 118.3(3) |  |
| C6 | C1 | N1 | 121.3(3) |  |
| C6 | C1 | C2 | 120.4(3) |  |
| C1 | C2 | O1 | 119.1(3) |  |
| C3 | C2 | O1 | 121.5(3) |  |
| C3 | C2 | C1 | 119.4(3) |  |
| H3a | C3 | C2 | 118(2) |  |
| C4 | C3 | C2 | 120.4(4) |  |
| C4 | C3 | H3a | 121(2) |  |
| H4 | C4 | C3 | 118(2) |  |
| C5 | C4 | C3 | 121.8(4) |  |
| C5 | C4 | H4 | 120(2) |  |
| H5 | C5 | C4 | 123(2) |  |
| C6 | C5 | C4 | 119.4(4) |  |
| C6 | C5 | H5 | 118(2) |  |
| C1 | C6 | N2 | 120.5(3) |  |
| C5 | C6 | N2 | 120.9(3) |  |
| C5 | C6 | C1 | 118.6(3) |  |
| C8 | C7 | N1 | 118.1(3) |  |
| C12 | C7 | N1 | 121.5(3) |  |
| C12 | C7 | C8 | 120.3(3) |  |
| C7 | C8 | O2 | 119.2(3) |  |
| C9 | C8 | O2 | 121.7(3) |  |
| C9 | C8 | C7 | 119.2(3) |  |
| H9 | C9 | C8 | 116.9(18) |  |
| C10 | C9 | C8 | 120.7(4) |  |
| C10 | C9 | H9 | 122.4(18) |  |
| H10 | C10 | C9 | 119.8(17) |  |
| C11 | C10 | C9 | 122.0(4) |  |
| C11 | C10 | H10 | 118.2(18) |  |
| H11 | C11 | C10 | 123.0(19) |  |
| C12 | C11 | C10 | 118.8(4) |  |
| C12 | C11 | H11 | 117.8(19) |  |
| C7 | C12 | N2 | 120.4(3) |  |
| C11 | C12 | N2 | 120.6(3) |  |
| C11 | C12 | C7 | 119.0(3) |  |
| C14 | O3 | H3 | 115(2) |  |
| C19 | N3 | C13 | 119.3(3) |  |
| C24 | N4 | C18 | 121.6(3) |  |
| C25 | N4 | C18 | 118.7(3) |  |
| C25 | N4 | C24 | 119.7(3) |  |
| C21 | C22 | H22 | 119(2) |  |
| C23 | C22 | H22 | 117(2) |  |
| C23 | C22 | C21 | 124.1(4) |  |
| C14 | C13 | N3 | 116.8(3) |  |
| C18 | C13 | N3 | 123.0(3) |  |
| C18 | C13 | C14 | 120.3(3) |  |
| C13 | C14 | O3 | 120.2(3) |  |
| C15 | C14 | O3 | 120.9(3) |  |
| C15 | C14 | C13 | 118.9(3) |  |
| H15 | C15 | C14 | 119(2) |  |
| C16 | C15 | C14 | 119.6(4) |  |
| C16 | C15 | H15 | 122(2) |  |
| H16 | C16 | C15 | 117.2(19) |  |
| C17 | C16 | C15 | 122.7(4) |  |
| C17 | C16 | H16 | 120.0(19) |  |
| H17 | C17 | C16 | 123(2) |  |
| C18 | C17 | C16 | 119.0(4) |  |
| C18 | C17 | H17 | 118(2) |  |
| C13 | C18 | N4 | 117.1(3) |  |
| C17 | C18 | N4 | 123.4(3) |  |
| C17 | C18 | C13 | 119.5(3) |  |
| C20 | C19 | N3 | 116.7(3) |  |
| C24 | C19 | N3 | 122.4(3) |  |
| C24 | C19 | C20 | 120.8(3) |  |
| C19 | C20 | O4 | 120.2(3) |  |
| C21 | C20 | O4 | 123.0(3) |  |
| C21 | C20 | C19 | 116.8(3) |  |
| C20 | C21 | C22 | 120.2(4) |  |
| H21 | C21 | C22 | 120.6(18) |  |
| H21 | C21 | C20 | 119.2(18) |  |
| H23 | C23 | C22 | 118.4(19) |  |
| C24 | C23 | C22 | 118.9(4) |  |
| C24 | C23 | H23 | 122.7(19) |  |
| C19 | C24 | N4 | 116.6(3) |  |
| C23 | C24 | N4 | 124.2(3) |  |
| C23 | C24 | C19 | 119.1(3) |  |
| H25a | C25 | N4 | 109.5 |  |
| H25b | C25 | N4 | 109.5 |  |
| H25b | C25 | H25a | 109.5 |  |
| H25c | C25 | N4 | 109.5 |  |
| H25c | C25 | H25a | 109.5 |  |
| H25c | C25 | H25b | 109.5 |  |

**Supplemental Table ST5**: Torsion Angles in ^°^ for **1+1b** heterodimer.

| **Atom** | **Atom** | **Atom** | **Atom** | **Angle/^°^** |  |
| --- | --- | --- | --- | --- | --- |
| O1 | C2 | C1 | N1 | -1.5(4) | |
| O1 | C2 | C1 | C6 | -179.9(3) | |
| O1 | C2 | C3 | C4 | 179.8(4) | |
| O2 | C8 | C7 | N1 | -0.6(4) | |
| O2 | C8 | C7 | C12 | 178.5(3) | |
| O2 | C8 | C9 | C10 | 179.7(4) | |
| N1 | C1 | C2 | C3 | 177.9(4) | |
| N1 | C1 | C6 | N2 | 1.4(4) | |
| N1 | C1 | C6 | C5 | -178.3(4) | |
| N1 | C7 | C8 | C9 | 179.5(3) | |
| N1 | C7 | C12 | N2 | 2.7(4) | |
| N1 | C7 | C12 | C11 | -178.9(3) | |
| N2 | C6 | C1 | C2 | 179.7(3) | |
| N2 | C6 | C5 | C4 | -179.2(4) | |
| N2 | C12 | C7 | C8 | -176.4(3) | |
| N2 | C12 | C11 | C10 | 177.6(4) | |
| C1 | C2 | C3 | C4 | 0.4(4) | |
| C1 | C6 | C5 | C4 | 0.5(4) | |
| C2 | C3 | C4 | C5 | 0.1(5) | |
| C3 | C4 | C5 | C6 | -0.6(5) | |
| C7 | C8 | C9 | C10 | -0.5(4) | |
| C7 | C12 | C11 | C10 | -0.9(4) | |
| C8 | C9 | C10 | C11 | 1.7(5) | |
| C9 | C10 | C11 | C12 | -1.0(5) | |
| O3 | C14 | C13 | N3 | -0.3(4) | |
| O3 | C14 | C13 | C18 | -179.7(3) | |
| O3 | C14 | C15 | C16 | 179.1(4) | |
| O4 | C20 | C19 | N3 | -2.8(4) | |
| O4 | C20 | C19 | C24 | 176.8(3) | |
| O4 | C20 | C21 | C22 | -176.9(4) | |
| N3 | C13 | C14 | C15 | 179.2(3) | |
| N3 | C13 | C18 | N4 | 1.3(4) | |
| N3 | C13 | C18 | C17 | -178.2(4) | |
| N3 | C19 | C20 | C21 | 178.0(3) | |
| N3 | C19 | C24 | N4 | 0.8(4) | |
| N3 | C19 | C24 | C23 | -179.7(4) | |
| N4 | C18 | C13 | C14 | -179.4(3) | |
| N4 | C18 | C17 | C16 | 179.1(4) | |
| N4 | C24 | C19 | C20 | -178.8(3) | |
| N4 | C24 | C23 | C22 | -179.5(4) | |
| C22 | C21 | C20 | C19 | 2.3(4) | |
| C22 | C23 | C24 | C19 | 1.1(4) | |
| C13 | C14 | C15 | C16 | -0.5(4) | |
| C13 | C18 | C17 | C16 | -1.4(4) | |
| C14 | C15 | C16 | C17 | 0.1(5) | |
| C15 | C16 | C17 | C18 | 0.8(5) | |

**Supplemental Table ST6**: Hydrogen Fractional Atomic Coordinates (×10^4^) and Equivalent Isotropic Displacement Parameters (Å^2^×10^3^) for **1+1b** heterodimer. *U_eq_* is defined as 1/3 of the trace of the orthogonalised *U_ij_*.

| **Atom** | **x** | **y** | **z** | ***U_eq_*** |
| --- | --- | --- | --- | --- |
| H1s | 470(50) | 1040(30) | 2742(12) | 55(9) |
| H1 | 6190(50) | 3337(6) | 4449(11) | 38(10) |
| H3a | 6060(60) | -170(30) | 4398(13) | 82(13) |
| H4 | 4390(80) | -1500(40) | 3832(17) | 150(20) |
| H5 | 2700(60) | -400(40) | 3191(14) | 100(15) |
| H9 | 2260(50) | 7240(40) | 3185(11) | 63(11) |
| H10 | 560(50) | 6080(40) | 2515(13) | 80(12) |
| H11 | 590(60) | 3590(40) | 2538(13) | 80(12) |
| H2 | 4870(40) | 5610(30) | 4065(8) | 33(7) |
| H3 | 7390(50) | 1740(30) | 4889(10) | 58(13) |
| H22 | 7520(60) | 8760(40) | 5149(13) | 88(15) |
| H15 | 9980(60) | -30(40) | 5757(14) | 93(15) |
| H16 | 11830(60) | 1130(40) | 6349(14) | 93(14) |
| H17 | 11860(70) | 3630(40) | 6376(17) | 129(18) |
| H21 | 5820(60) | 7530(30) | 4566(13) | 81(13) |
| H23 | 9430(60) | 7790(30) | 5730(14) | 90(14) |
| H25a | 10419(8) | 6796(13) | 6180.1(13) | 68(5) |
| H25b | 10763(6) | 5386(7) | 6473(4) | 67(5) |
| H25c | 12197(18) | 5850(4) | 6092.6(15) | 65(5) |

**Supplemental Table ST7**: Hydrogen Bond information for **1+1b** heterodimer.

| **D** | **H** | **A** | **d(D-H)/Å** | **d(H-A)/Å** | **d(D-A)/Å** | **D-H-A/deg** |
| --- | --- | --- | --- | --- | --- | --- |
| O1 | H1 | O4 | 1.0698(18) | 1.688(4) | 2.756(3) | 177(3) |
| O2 | H2 | O4 | 1.0698(18) | 1.677(7) | 2.735(3) | 169(3) |
| O3 | H3 | O1 | 1.0698(18) | 1.662(12) | 2.706(3) | 164(3) |

**Supplemental Table ST8**: Fractional Atomic Coordinates (×10^4^) and Equivalent Isotropic Displacement Parameters (Å^2^×10^3^) for **1b** dimer. *U_eq_* is defined as 1/3 of the trace of the orthogonalised *U_ij_*.

| Atom | x | y | z | *U_eq_* |
| --- | --- | --- | --- | --- |
| O2B | 5715(10) | 3568(4) | 1333(5) | 81(3) |
| O1B | 2302(9) | 1370(5) | 1522(5) | 75(3) |
| O1 | 1972(9) | 3742(5) | 7361(4) | 81(2) |
| O2 | 5169(9) | 1459(5) | 7040(4) | 77(2) |
| O1W | 4095(10) | 2977(5) | 6189(5) | 92(3) |
| N1 | 3672(11) | 2507(5) | 8144(6) | 68(2) |
| N1B | 3967(11) | 2578(5) | 2351(5) | 64(2) |
| N2 | 3873(11) | 2287(5) | 10072(6) | 65(2) |
| N2B | 3983(11) | 2811(5) | 4261(6) | 68(2) |
| C12 | 4628(13) | 1786(7) | 9516(7) | 62(3) |
| C7 | 4508(13) | 1912(7) | 8544(7) | 64(3) |
| C7B | 4804(13) | 3186(7) | 2763(7) | 66(3) |
| C2B | 2283(13) | 1422(7) | 2437(7) | 64(3) |
| C1 | 2905(13) | 3015(7) | 8697(7) | 61(3) |
| C6B | 3183(14) | 2184(7) | 3863(8) | 64(3) |
| C8 | 5307(14) | 1337(7) | 7958(7) | 70(3) |
| C1B | 3175(13) | 2078(7) | 2877(7) | 61(3) |
| C2 | 1971(14) | 3664(7) | 8289(8) | 71(3) |
| C5B | 2322(13) | 1610(7) | 4361(7) | 72(3) |
| C12B | 4761(13) | 3313(7) | 3739(7) | 69(3) |
| C9B | 6539(14) | 4357(7) | 2634(7) | 80(3) |
| C9 | 6174(14) | 702(7) | 8331(7) | 75(3) |
| C11B | 5622(14) | 3981(7) | 4130(7) | 77(3) |
| C8B | 5686(13) | 3726(7) | 2225(7) | 65(3) |
| C6 | 2992(14) | 2905(7) | 9678(7) | 63(3) |
| C5 | 2114(14) | 3453(7) | 10210(7) | 74(3) |
| C11 | 5569(14) | 1124(7) | 9875(7) | 74(3) |
| C10 | 6312(14) | 604(7) | 9306(7) | 79(3) |
| C3 | 1147(14) | 4189(6) | 8824(7) | 76(3) |
| C10B | 6471(14) | 4486(7) | 3597(7) | 78(3) |
| C3B | 1461(14) | 894(7) | 2955(7) | 75(3) |
| C4 | 1253(14) | 4063(7) | 9798(7) | 81(3) |
| C4B | 1471(14) | 989(7) | 3914(7) | 74(3) |

**Supplemental Table ST9**: Anisotropic Displacement Parameters (×10^4^) for **1b** dimer. The anisotropic displacement factor exponent takes the form: *-2p^2^[h^2^a*^2^ × U_11_+ ... +2hka* × b* × U_12_]*

| Atom | *U_11_* | *U_22_* | *U_33_* | *U_23_* | *U_13_* | *U_12_* |
| --- | --- | --- | --- | --- | --- | --- |
| O2B | 109(7) | 84(6) | 51(2) | -15(4) | 19.9(17) | 0.4(15) |
| O1B | 103(6) | 71(5) | 52(3) | -9(3) | 20.4(16) | 1.9(14) |
| O1 | 107(7) | 82(5) | 57(3) | 2(4) | 20.0(16) | 8.2(14) |
| O2 | 93(7) | 84(6) | 55(2) | 4(3) | 18.4(16) | -0.7(14) |
| O1W | 128(7) | 99(6) | 50(5) | 12(6) | 23(5) | 0(5) |
| N1 | 89(5) | 66(4) | 50(3) | -5(2) | 16.2(15) | 1.9(13) |
| N1B | 80(5) | 67(4) | 47(3) | 6(2) | 14.8(15) | 4.1(13) |
| N2 | 90(5) | 58(4) | 50(3) | -7(2) | 17.7(15) | 1.6(13) |
| N2B | 85(5) | 69(4) | 51(3) | 7(2) | 14.9(15) | 5.7(13) |
| C12 | 82(5) | 55(4) | 49(2) | -12(2) | 15.9(14) | 0.6(12) |
| C7 | 81(6) | 62(4) | 50(2) | -11(2) | 16.1(14) | 1.5(12) |
| C7B | 84(6) | 68(4) | 48(3) | 4(2) | 13.3(15) | 4.5(13) |
| C2B | 76(6) | 65(4) | 52(3) | 10(2) | 17.6(15) | 3.6(14) |
| C1 | 76(5) | 57(4) | 50(2) | -16(2) | 16.6(14) | 1.8(13) |
| C6B | 77(6) | 67(4) | 50(3) | 11(2) | 16.8(14) | 4.9(12) |
| C8 | 87(6) | 69(4) | 54(2) | -7(2) | 16.9(16) | -2.6(13) |
| C1B | 72(5) | 61(4) | 50(3) | 14(2) | 16.2(14) | 5.6(13) |
| C2 | 90(6) | 67(4) | 56(3) | -5(2) | 18.5(15) | 6.4(14) |
| C5B | 87(6) | 73(4) | 58(3) | 6(2) | 20.6(18) | 8.1(15) |
| C12B | 88(6) | 70(4) | 48(3) | 4(2) | 13.7(15) | 4.2(13) |
| C9B | 103(7) | 80(5) | 57(3) | -9(3) | 15.6(19) | -0.1(17) |
| C9 | 95(7) | 72(5) | 60(3) | -3(3) | 14.8(19) | -1.2(16) |
| C11B | 99(6) | 76(4) | 55(3) | -3(2) | 12.4(18) | 1.6(16) |
| C8B | 81(6) | 67(4) | 50(3) | 7(2) | 13.7(16) | 5.3(14) |
| C6 | 84(5) | 55(4) | 50(2) | -12(2) | 17.2(14) | 1.6(13) |
| C5 | 101(6) | 67(5) | 55(3) | 0(2) | 20.6(18) | 0.3(15) |
| C11 | 102(6) | 67(4) | 56(3) | 2(2) | 15.8(18) | 3.5(15) |
| C10 | 104(7) | 74(5) | 60(3) | 5(3) | 15.3(19) | -1.2(16) |
| C3 | 96(6) | 72(5) | 61(3) | -2(3) | 20.3(18) | 4.0(16) |
| C10B | 102(7) | 76(5) | 57(3) | -3(3) | 14.6(19) | 1.1(16) |
| C3B | 93(7) | 76(5) | 59(3) | 1(3) | 22.3(19) | 6.3(16) |
| C4 | 110(7) | 74(5) | 61(3) | 7(3) | 21.5(18) | 4.2(16) |
| C4B | 90(7) | 76(5) | 59(3) | 4(3) | 21.8(19) | 6.4(16) |

**Supplemental Table ST10**: Bond Lengths in Å for **1b** dimer.

| Atom | Atom | Length/Å | |
| --- | --- | --- | --- |
| O2B | H2B | 1.28(13) |  |
| O2B | C8B | 1.330(11) |  |
| O1B | H1B | 1.13(12) |  |
| O1B | C2B | 1.342(11) |  |
| O1 | H1 | 0.9950 |  |
| O1 | C2 | 1.362(11) |  |
| O2 | H2 | 0.9950 |  |
| O2 | C8 | 1.355(11) |  |
| O1W | H1Wa | 0.995(2) |  |
| O1W | H1Wb | 0.9750(19) |  |
| N1 | C7 | 1.314(12) |  |
| N1 | C1 | 1.345(12) |  |
| N1B | C7B | 1.339(12) |  |
| N1B | C1B | 1.324(11) |  |
| N2 | C12 | 1.336(11) |  |
| N2 | C6 | 1.358(12) |  |
| N2B | C6B | 1.339(13) |  |
| N2B | C12B | 1.316(11) |  |
| C12 | C7 | 1.435(13) |  |
| C12 | C11 | 1.420(13) |  |
| C7 | C8 | 1.457(13) |  |
| C7B | C12B | 1.444(13) |  |
| C7B | C8B | 1.416(13) |  |
| C2B | C1B | 1.437(14) |  |
| C2B | C3B | 1.358(13) |  |
| C1 | C2 | 1.428(14) |  |
| C1 | C6 | 1.444(13) |  |
| C6B | C1B | 1.450(13) |  |
| C6B | C5B | 1.408(13) |  |
| C8 | C9 | 1.362(13) |  |
| C2 | C3 | 1.372(13) |  |
| C5B | H5B | 1.1030 |  |
| C5B | C4B | 1.382(13) |  |
| C12B | C11B | 1.408(14) |  |
| C9B | H9B | 1.1030 |  |
| C9B | C8B | 1.372(13) |  |
| C9B | C10B | 1.427(12) |  |
| C9 | H9 | 1.1030 |  |
| C9 | C10 | 1.432(13) |  |
| C11B | H11B | 1.1030 |  |
| C11B | C10B | 1.362(12) |  |
| C6 | C5 | 1.417(13) |  |
| C5 | H5 | 1.1030 |  |
| C5 | C4 | 1.351(13) |  |
| C11 | H11 | 1.1030 |  |
| C11 | C10 | 1.363(12) |  |
| C10 | H10 | 1.1030 |  |
| C3 | H3 | 1.1030 |  |
| C3 | C4 | 1.436(13) |  |
| C10B | H10B | 1.1030 |  |
| C3B | H3B | 1.1030 |  |
| C3B | C4B | 1.409(12) |  |
| C4 | H4 | 1.1030 |  |
| C4B | H4B | 1.1030 |  |

**Supplemental Table ST11**: Bond Angles in ^°^ for **1b** dimer.

| Atom | Atom | Atom | Angle/^°^ | |
| --- | --- | --- | --- | --- |
| C8B | O2B | H2B | 112(5) |  |
| C2B | O1B | H1B | 119(6) |  |
| C2 | O1 | H1 | 109.5 |  |
| C8 | O2 | H2 | 109.5 |  |
| H1Wb | O1W | H1Wa | 107.8(10) |  |
| C1 | N1 | C7 | 116.6(10) |  |
| C1B | N1B | C7B | 117.5(10) |  |
| C6 | N2 | C12 | 117.4(9) |  |
| C12B | N2B | C6B | 118.3(11) |  |
| C7 | C12 | N2 | 120.3(11) |  |
| C11 | C12 | N2 | 120.8(10) |  |
| C11 | C12 | C7 | 118.9(11) |  |
| C12 | C7 | N1 | 123.6(11) |  |
| C8 | C7 | N1 | 117.6(10) |  |
| C8 | C7 | C12 | 118.7(12) |  |
| C12B | C7B | N1B | 120.6(11) |  |
| C8B | C7B | N1B | 119.1(11) |  |
| C8B | C7B | C12B | 120.2(12) |  |
| C1B | C2B | O1B | 117.3(10) |  |
| C3B | C2B | O1B | 123.4(12) |  |
| C3B | C2B | C1B | 119.3(11) |  |
| C2 | C1 | N1 | 118.3(11) |  |
| C6 | C1 | N1 | 121.4(11) |  |
| C6 | C1 | C2 | 120.3(11) |  |
| C1B | C6B | N2B | 119.9(11) |  |
| C5B | C6B | N2B | 122.7(11) |  |
| C5B | C6B | C1B | 117.4(11) |  |
| C7 | C8 | O2 | 118.0(11) |  |
| C9 | C8 | O2 | 121.6(11) |  |
| C9 | C8 | C7 | 120.3(11) |  |
| C2B | C1B | N1B | 117.6(10) |  |
| C6B | C1B | N1B | 121.9(11) |  |
| C6B | C1B | C2B | 120.4(11) |  |
| C1 | C2 | O1 | 117.1(10) |  |
| C3 | C2 | O1 | 122.5(11) |  |
| C3 | C2 | C1 | 120.4(11) |  |
| H5B | C5B | C6B | 119.8(7) |  |
| C4B | C5B | C6B | 120.5(11) |  |
| C4B | C5B | H5B | 119.8(7) |  |
| C7B | C12B | N2B | 121.6(12) |  |
| C11B | C12B | N2B | 120.3(11) |  |
| C11B | C12B | C7B | 118.0(11) |  |
| C8B | C9B | H9B | 120.3(7) |  |
| C10B | C9B | H9B | 120.3(7) |  |
| C10B | C9B | C8B | 119.4(11) |  |
| H9 | C9 | C8 | 120.1(7) |  |
| C10 | C9 | C8 | 119.8(11) |  |
| C10 | C9 | H9 | 120.1(7) |  |
| H11B | C11B | C12B | 119.7(7) |  |
| C10B | C11B | C12B | 120.5(11) |  |
| C10B | C11B | H11B | 119.7(7) |  |
| C7B | C8B | O2B | 117.7(11) |  |
| C9B | C8B | O2B | 122.3(11) |  |
| C9B | C8B | C7B | 120.0(11) |  |
| C1 | C6 | N2 | 120.7(10) |  |
| C5 | C6 | N2 | 121.5(10) |  |
| C5 | C6 | C1 | 117.9(12) |  |
| H5 | C5 | C6 | 120.0(7) |  |
| C4 | C5 | C6 | 120.0(11) |  |
| C4 | C5 | H5 | 120.0(7) |  |
| H11 | C11 | C12 | 119.7(6) |  |
| C10 | C11 | C12 | 120.7(11) |  |
| C10 | C11 | H11 | 119.7(7) |  |
| C11 | C10 | C9 | 121.5(12) |  |
| H10 | C10 | C9 | 119.2(7) |  |
| H10 | C10 | C11 | 119.2(7) |  |
| H3 | C3 | C2 | 121.0(7) |  |
| C4 | C3 | C2 | 118.0(12) |  |
| C4 | C3 | H3 | 121.0(7) |  |
| C11B | C10B | C9B | 121.8(12) |  |
| H10B | C10B | C9B | 119.1(7) |  |
| H10B | C10B | C11B | 119.1(7) |  |
| H3B | C3B | C2B | 119.7(7) |  |
| C4B | C3B | C2B | 120.6(12) |  |
| C4B | C3B | H3B | 119.7(7) |  |
| C3 | C4 | C5 | 123.4(11) |  |
| H4 | C4 | C5 | 118.3(7) |  |
| H4 | C4 | C3 | 118.3(7) |  |
| C3B | C4B | C5B | 121.8(12) |  |
| H4B | C4B | C5B | 119.1(7) |  |
| H4B | C4B | C3B | 119.1(7) |  |

**Supplemental Table ST12**: Torsion Angles in ^°^ for **1b** dimer.

| Atom | Atom | Atom | Atom | Angle/^°^ |  |
| --- | --- | --- | --- | --- | --- |
| O2B | C8B | C7B | N1B | -4.0(11) | |
| O2B | C8B | C7B | C12B | 178.4(9) | |
| O2B | C8B | C9B | C10B | -179.2(11) | |
| O1B | C2B | C1B | N1B | -0.9(11) | |
| O1B | C2B | C1B | C6B | 178.7(9) | |
| O1B | C2B | C3B | C4B | -179.1(11) | |
| O1 | C2 | C1 | N1 | -2.4(11) | |
| O1 | C2 | C1 | C6 | 178.6(9) | |
| O1 | C2 | C3 | C4 | -178.7(11) | |
| O2 | C8 | C7 | N1 | 1.4(12) | |
| O2 | C8 | C7 | C12 | 179.3(9) | |
| O2 | C8 | C9 | C10 | 178.9(11) | |
| N1 | C7 | C12 | N2 | -0.3(13) | |
| N1 | C7 | C12 | C11 | -179.8(11) | |
| N1 | C7 | C8 | C9 | -179.2(11) | |
| N1 | C1 | C2 | C3 | 179.0(11) | |
| N1 | C1 | C6 | N2 | 0.8(12) | |
| N1 | C1 | C6 | C5 | -178.3(11) | |
| N1B | C7B | C12B | N2B | 4.1(12) | |
| N1B | C7B | C12B | C11B | -177.8(11) | |
| N1B | C7B | C8B | C9B | 179.4(11) | |
| N1B | C1B | C2B | C3B | 179.7(10) | |
| N1B | C1B | C6B | N2B | 1.4(12) | |
| N1B | C1B | C6B | C5B | -179.0(10) | |
| N2 | C12 | C7 | C8 | -178.1(10) | |
| N2 | C12 | C11 | C10 | 178.7(11) | |
| N2 | C6 | C1 | C2 | 179.8(10) | |
| N2 | C6 | C5 | C4 | 179.8(11) | |
| N2B | C6B | C1B | C2B | -178.1(10) | |
| N2B | C6B | C5B | C4B | 177.8(11) | |
| N2B | C12B | C7B | C8B | -178.4(11) | |
| N2B | C12B | C11B | C10B | 177.9(11) | |
| C12 | N2 | C6 | C1 | -0.9(11) | |
| C12 | N2 | C6 | C5 | 178.2(10) | |
| C12 | C7 | N1 | C1 | 0.1(12) | |
| C12 | C7 | C8 | C9 | -1.3(12) | |
| C12 | C11 | C10 | C9 | -0.1(13) | |
| C7 | N1 | C1 | C2 | -179.4(10) | |
| C7 | N1 | C1 | C6 | -0.4(12) | |
| C7 | C12 | N2 | C6 | 0.7(12) | |
| C7 | C12 | C11 | C10 | -1.8(12) | |
| C7 | C8 | C9 | C10 | -0.5(12) | |
| C7B | N1B | C1B | C2B | -179.7(10) | |
| C7B | N1B | C1B | C6B | 0.8(11) | |
| C7B | C12B | N2B | C6B | -1.8(13) | |
| C7B | C12B | C11B | C10B | -0.2(12) | |
| C7B | C8B | C9B | C10B | -2.8(12) | |
| C2B | C1B | C6B | C5B | 1.5(12) | |
| C2B | C3B | C4B | C5B | -0.7(13) | |
| C1 | N1 | C7 | C8 | 177.9(10) | |
| C1 | C2 | C3 | C4 | -0.1(14) | |
| C1 | C6 | C5 | C4 | -1.1(12) | |
| C6B | N2B | C12B | C11B | -179.8(11) | |
| C6B | C1B | C2B | C3B | -0.8(12) | |
| C6B | C5B | C4B | C3B | 1.5(13) | |
| C8 | C7 | C12 | C11 | 2.4(12) | |
| C8 | C9 | C10 | C11 | 1.2(13) | |
| C1B | N1B | C7B | C12B | -3.4(11) | |
| C1B | N1B | C7B | C8B | 179.0(10) | |
| C1B | C2B | C3B | C4B | 0.4(12) | |
| C1B | C6B | N2B | C12B | -0.8(12) | |
| C1B | C6B | C5B | C4B | -1.8(12) | |
| C2 | C1 | C6 | C5 | 0.7(13) | |
| C2 | C3 | C4 | C5 | -0.3(13) | |
| C5B | C6B | N2B | C12B | 179.6(11) | |
| C12B | C7B | C8B | C9B | 1.8(12) | |
| C12B | C11B | C10B | C9B | -0.8(14) | |
| C11B | C12B | C7B | C8B | -0.3(13) | |
| C11B | C10B | C9B | C8B | 2.3(13) | |
| C6 | N2 | C12 | C11 | -179.8(10) | |
| C6 | C1 | C2 | C3 | -0.0(12) | |
| C6 | C5 | C4 | C3 | 0.9(13) | |

**Supplemental Table ST13**: Hydrogen Fractional Atomic Coordinates (×10^4^) and Equivalent Isotropic Displacement Parameters (Å^2^×10^3^) for **1b** dimer. *U_eq_* is defined as 1/3 of the trace of the orthogonalised *U_ij_*.

| Atom | x | y | z | *U_eq_* |
| --- | --- | --- | --- | --- |
| H2B | 4690(150) | 3030(80) | 1070(80) | 121(4) |
| H1B | 3080(140) | 1800(80) | 1140(80) | 112(4) |
| H1 | 890(40) | 3890(90) | 7116(11) | 122(4) |
| H2 | 4330(120) | 1870(70) | 6895(9) | 115(4) |
| H1Wa | 3090(60) | 3160(70) | 6440(50) | 138(4) |
| H1Wb | 3920(100) | 2920(90) | 5527(12) | 138(4) |
| H5B | 2326(13) | 1657(7) | 5114(7) | 87(4) |
| H9B | 7271(14) | 4762(7) | 2222(7) | 96(4) |
| H9 | 6769(14) | 264(7) | 7885(7) | 90(4) |
| H11B | 5610(14) | 4095(7) | 4873(7) | 92(4) |
| H5 | 2133(14) | 3382(7) | 10961(7) | 88(3) |
| H11 | 5699(14) | 1031(7) | 10623(7) | 89(4) |
| H10 | 7037(14) | 95(7) | 9602(7) | 95(4) |
| H3 | 424(14) | 4694(6) | 8516(7) | 91(4) |
| H10B | 7122(14) | 5010(7) | 3917(7) | 93(4) |
| H3B | 779(14) | 386(7) | 2623(7) | 91(4) |
| H4 | 597(14) | 4486(7) | 10231(7) | 97(4) |
| H4B | 783(14) | 556(7) | 4317(7) | 89(4) |

**Supplemental Table ST14**: Hydrogen Bond information for **1b** dimer.

| D | H | A | d(D-H)/Å | d(H-A)/Å | d(D-A)/Å | D-H-A/deg |
| --- | --- | --- | --- | --- | --- | --- |
| O2B | H2B | N2^1^ | 1.28(13) | 1.98(13) | 3.136(11) | 147(8) |
| O1B | H1B | N2^1^ | 1.13(12) | 1.90(12) | 2.962(10) | 154(9) |
| O2 | H2 | O1W | 0.9950 | 2.07(8) | 2.879(11) | 137(10) |
| O1W | H1Wa | O1 | 0.995(2) | 1.93(4) | 2.836(10) | 150(7) |
| O1W | H1Wb | N2B | 0.9750(19) | 1.862(17) | 2.827(11) | 170(7) |
| C5B | H5B | O2B^2^ | 1.1030 | 2.331(12) | 3.266(12) | 141.3(4) |
| C5 | H5 | O2^2^ | 1.1030 | 2.369(12) | 3.219(12) | 132.6(3) |

**SUPPLEMENTAL FIGURES**

**Supplemental Figure SF1. Antimicrobial performance of Luedemann isolate 9176P toward streptomycin susceptible and streptomycin resistant *S. aureus*.** Antimicrobial activity of 9176P supernatant (test), the antibiotics albocycline, methicillin, vancomycin (positive controls) and streptomycin (negative control) as measured on agar plates preinoculated with streptomycin susceptible (**Panel A; left**) or resistant (**right**) *S. aureus* strains. MTT HepG2 cytotoxicity measures of pyocyanin A, pyocyanin and mitomycin C (125 µg/mL; positive control) (**Panel B**).

**A.**


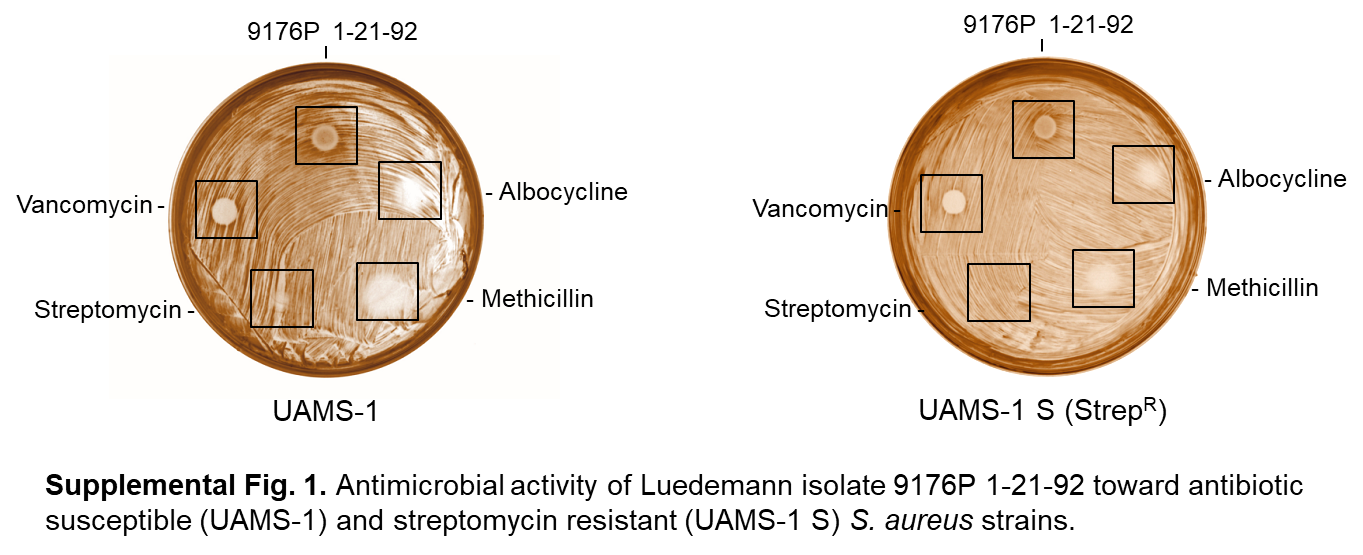


**B.**

**
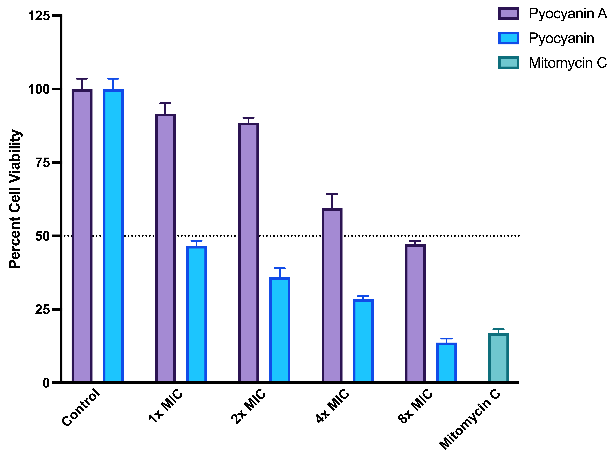
**

**Supplemental Figure SF2.** Small (**Panel A**) and large-scale (**Panel B**) fractionation schemes to produce compound **1**.


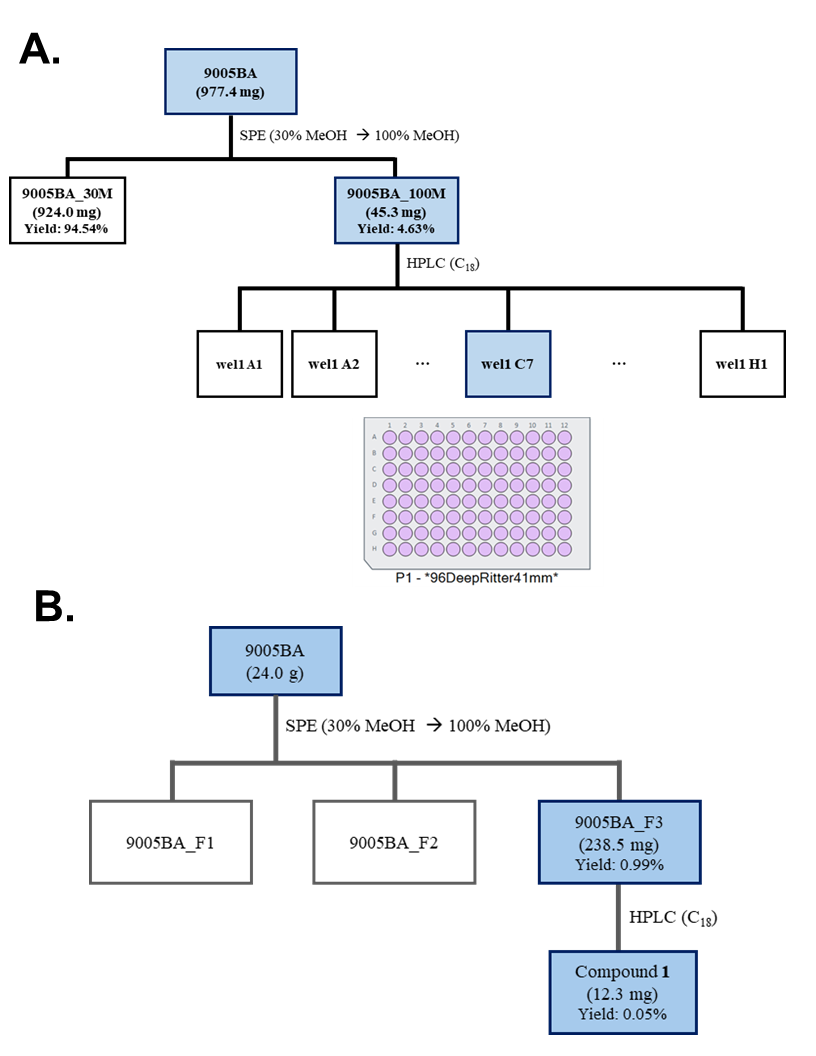


**Supplemental Figure SF3.** HPLC-DAD chromatogram. (A): Small-scale fractionation of 9005BA_30M into 96-well microplate, (B): Large-scale fractionation of 9005BA_F3 to produce compound **1** (peak in blue column).


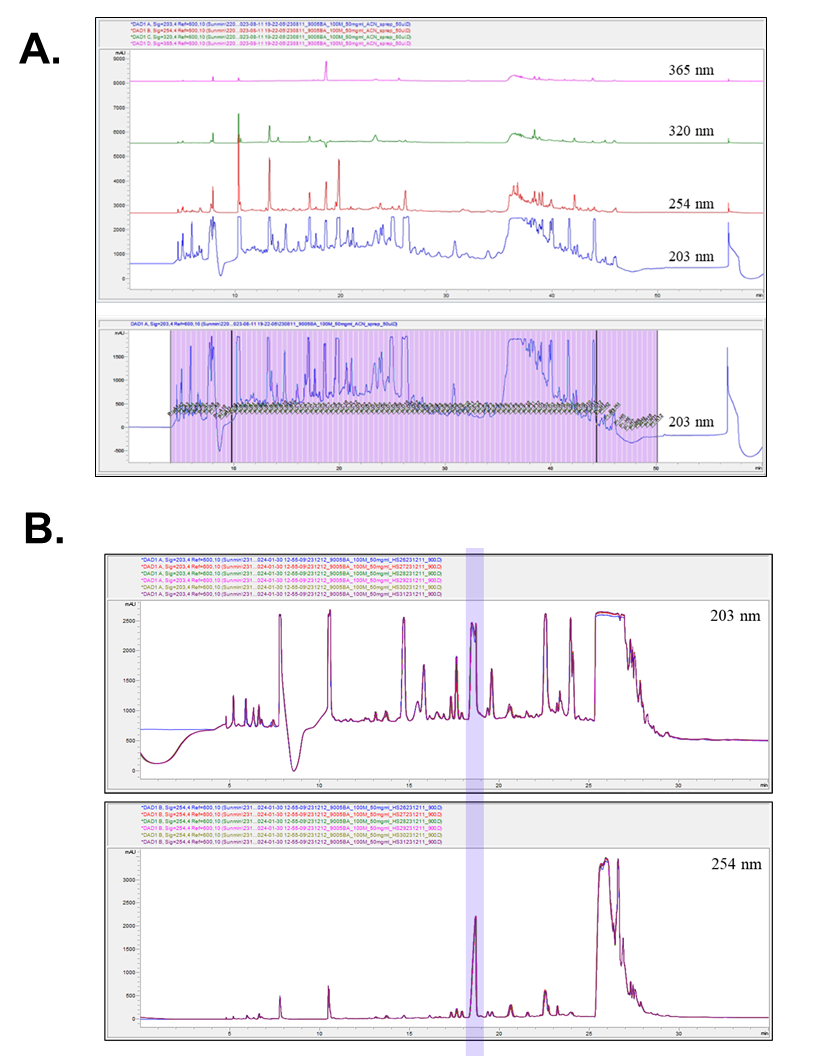


**Supplemental Figure SF4.** (A) HRESIMS data of compound **1**. (B) UV spectrum of compound **1**.

**A.**


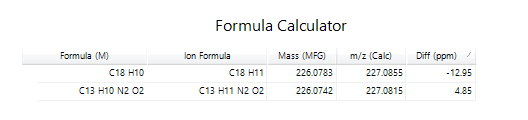


**B.**


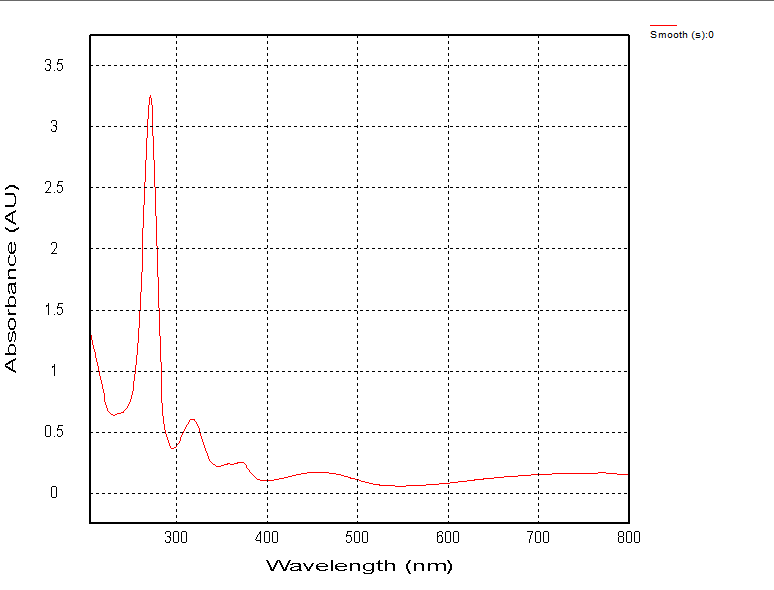


**Supplemental Figure SF5.** HRMS data by direct injection method at different pH conditions and color changes.

(A) HRMS data of compound **1** under low pH condition based on 10% formic acid in MeOH. (B) HRMS data of compound **1** under high pH condition based on 25% ammonia solution. (C) HRMS data of a sample dissolved in MeOH solution after re-drying the solution with high pH with 25% ammonia solution. (D) color changes at high concentration (0.2 mg/mL) at different pH condition.

**A.**


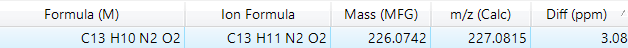


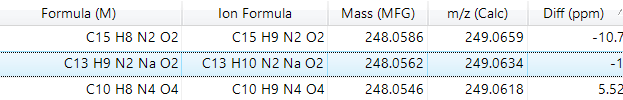


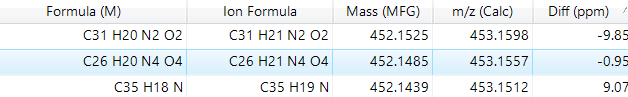

**B.**

**
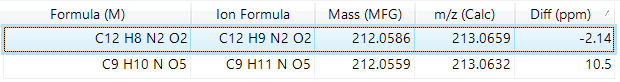
**

**
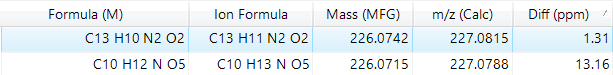
**

**C.**


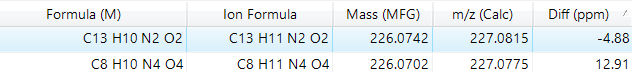

**D.**

**
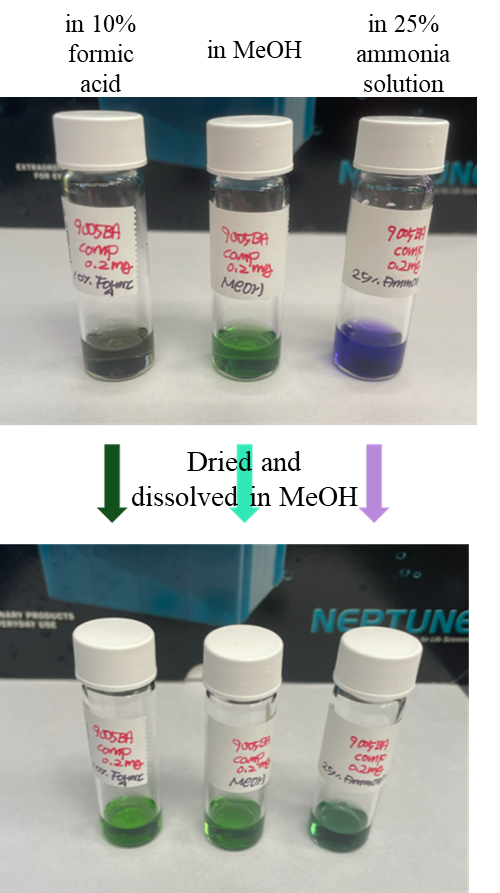
**

**Supplemental Figure SF6.** ^1^H NMR spectrum of compound **1** (800 MHz, in MeOD).


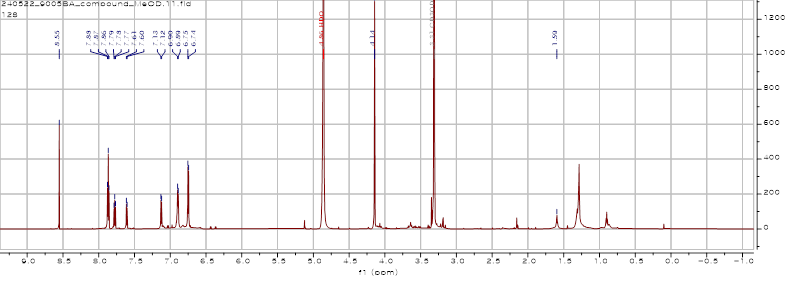


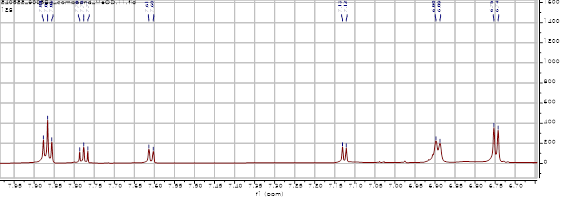


**Supplemental Figure SF7.** ^13^C NMR spectrum of compound **1** (200 MHz, in MeOD).


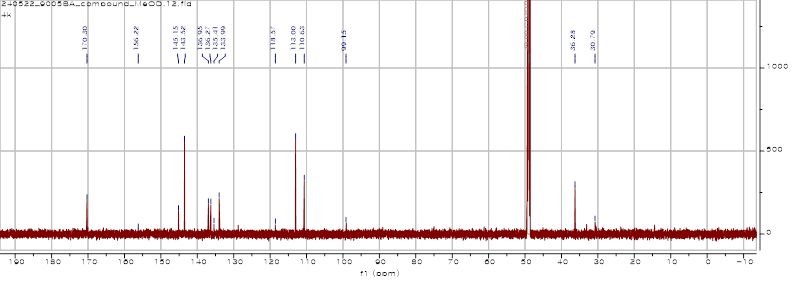


**Supplemental Figure SF8.** ^1^H–^1^H COSY spectrum of compound **1** (800 MHz, in MeOD).


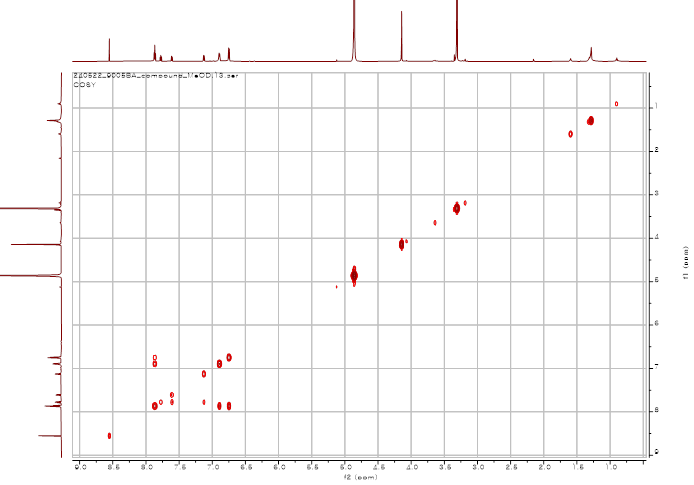


**Supplemental Figure SF9.** Multiplicity-edited HSQC spectrum of compound **1** (800 MHz, in MeOD).


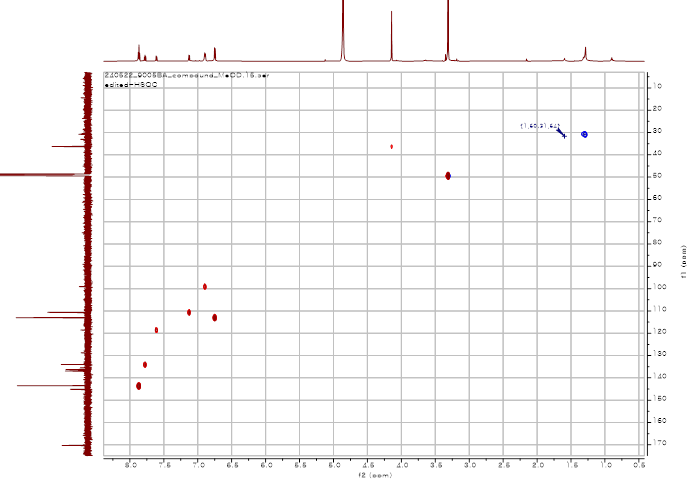


**Supplemental Figure SF10.** HMBC spectrum of compound **1** (800 MHz, in MeOD).


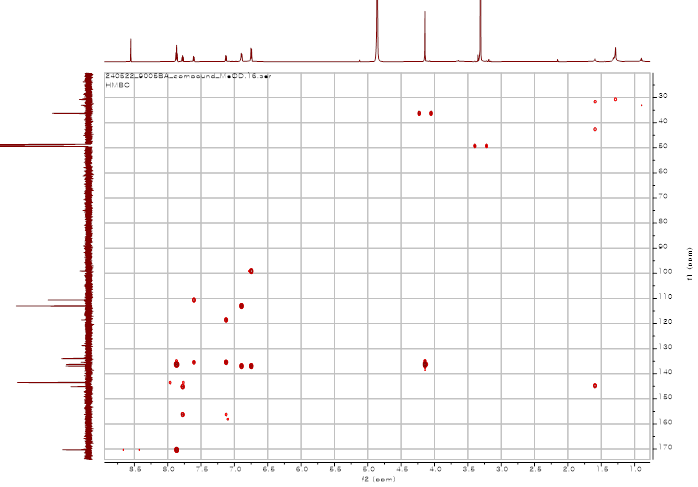


**Supplemental Figure SF11.** ^1^H NMR spectrum of compound **1** (800 MHz, in acetone-*d*_6_).


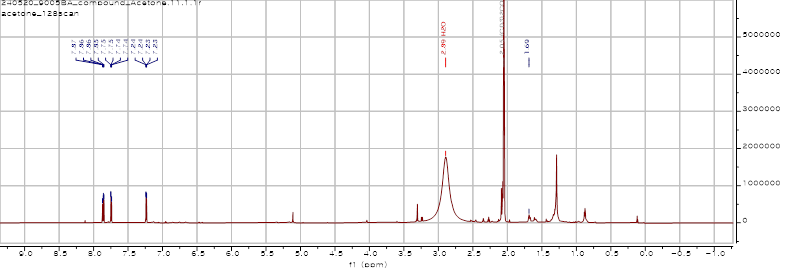


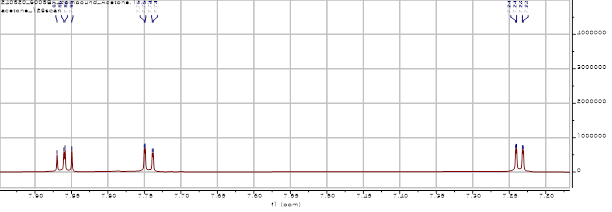


**Supplemental Figure S12.** ^13^C NMR spectrum of compound **1** (200 MHz, in acetone-*d*_6_).


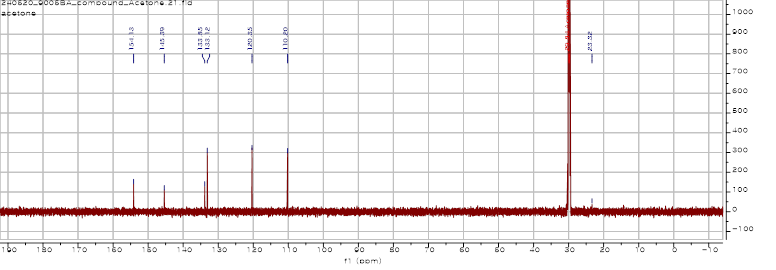


**Supplemental Figure SF13.** ^1^H–^1^H COSY spectrum of compound **1** (800 MHz, in acetone-*d*_6_).


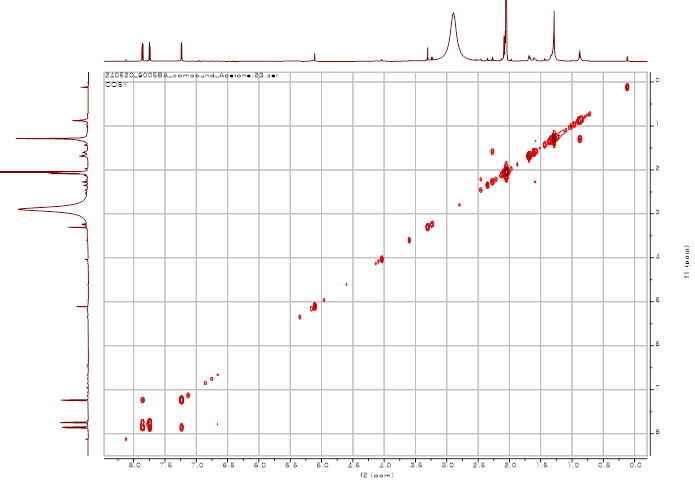


**Supplemental Figure SF14.** Multiplicity-edited HSQC spectrum of compound **1** (800 MHz, in acetone-*d*_6_).


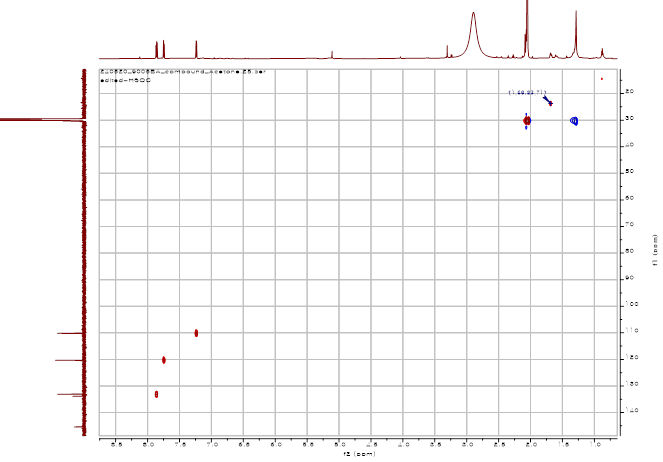


**Supplemental Figure SF15.** HMBC spectrum of compound **1** (800 MHz, in acetone-*d*_6_).


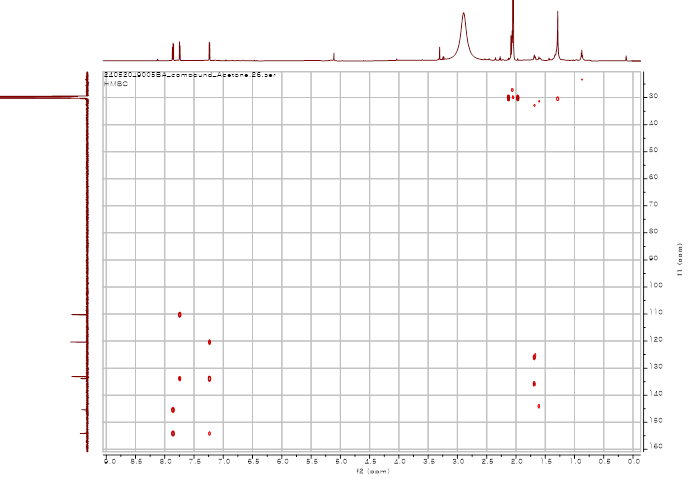


**Supplemental Figure SF16.** Crystal data and experimental details for **1** and **1b** heterodimer.

Crystal Data and Experimental


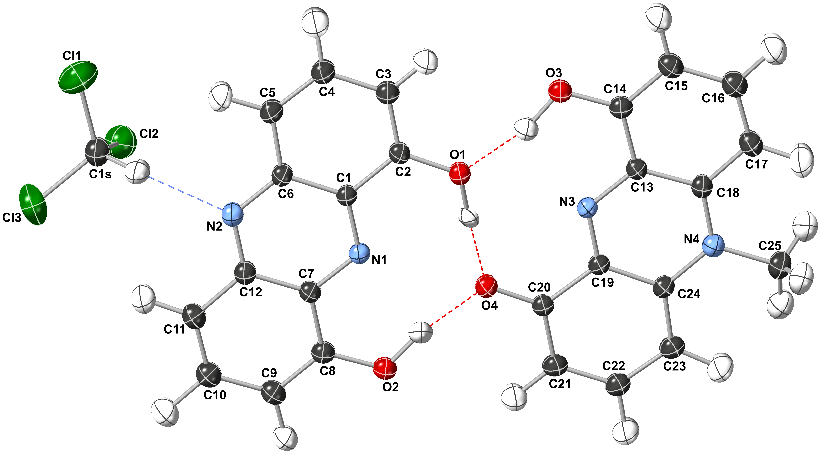


**Experimental.** Single very dark green needle-shaped crystals of **1** and **1b** heterodimer were recrystallized from chloroform by slow evaporation. A suitable crystal with dimensions 0.28 × 0.04 × 0.02 mm^3^ was selected and mounted on a loop with paratone on a XtaLAB AFC11 (RCD3) quarter-chi single diffractometer. The crystal was kept at a steady *T* = 173(1) K during data collection. The structure was solved with the ShelXT (Sheldrick, 2015) solution program using iterative methods and by using Olex2 1.5-alpha (Dolomanov et al., 2009) as the graphical interface. The model was refined with olex2.refine 1.5-alpha (Bourhis et al., 2015) using full matrix least squares minimisation on ***F*^2^**.

**Crystal Data.** C_26_H_19_Cl_3_N_4_O_4_, *M_r_* = 557.823, monoclinic, *P*2_1_/*n* (No. 14), a = 7.6650(8) Å, b = 10.2857(7) Å, c = 30.161(2) Å, *β* = 93.814(8)^°^, *α* = *γ* = 90^°^, *V* = 2372.6(4) Å^3^, *T* = 173.04(16) K, *Z* = 4, *Z'* = 1, *μ*(Cu K*_α_*) = 3.874, 43737 reflections measured, 4310 unique (R_int_ = 0.1089) which were used in all calculations. The final *wR_2_* was 0.1735 (all data) and *R_1_* was 0.0605 (I≥2 *σ*(I)).

| **Compound** | **1** and **1b** heterodimer. |
| --- | --- |
|  |  |
| Formula | C_26_H_19_Cl_3_N_4_O_4_ |
| *D_calc._*/ g cm^-3^ | 1.562 |
| *μ*/mm^-1^ | 3.874 |
| Formula Weight | 557.823 |
| Colour | dark green |
| Shape | needle-shaped |
| Size/mm^3^ | 0.28×0.04×0.02 |
| *T*/K | 173.04(16) |
| Crystal System | monoclinic |
| Space Group | *P*2_1_/*n* |
| *a*/Å | 7.6650(8) |
| *b*/Å | 10.2857(7) |
| *c*/Å | 30.161(2) |
| *α*/^°^ | 90 |
| *β*/^°^ | 93.814(8) |
| *γ*/^°^ | 90 |
| V/Å^3^ | 2372.6(4) |
| *Z* | 4 |
| *Z'* | 1 |
| Wavelength/Å | 1.54184 |
| Radiation type | Cu K*_α_* |
| *Θ_min_*/^°^ | 4.54 |
| *Θ_max_*/^°^ | 68.24 |
| Measured Refl's. | 43737 |
| Indep't Refl's | 4310 |
| Refl's I≥2 *σ*(I) | 2174 |
| *R*_int_ | 0.1089 |
| Parameters | 497 |
| Restraints | 718 |
| Largest Peak | 0.7023 |
| Deepest Hole | -0.5728 |
| GooF | 1.0206 |
| *wR_2_* (all data) | 0.1735 |
| *wR_2_* | 0.1374 |
| *R_1_* (all data) | 0.1307 |
| *R_1_* | 0.0605 |

**Supplemental Figure SF17.** Structural quality indicators for **1** and **1b** heterodimer.

**Structure Quality Indicators**

| **Reflections:** | 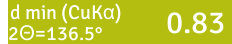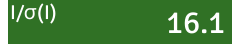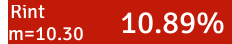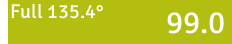 |
| --- | --- |
| **Refinement:** | 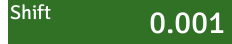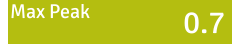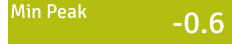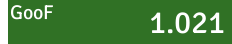 |

A dark green needle-shaped-shaped crystal with dimensions 0.28 × 0.04 × 0.02 mm^3^ was mounted on a loop with paratone. Data were collected using a XtaLAB AFC11 (RCD3): quarter-chi single diffractometer equipped with an Oxford Cryosystems low-temperature device operating at *T* = 173.04(16) K.

Data were measured using *ω* scans with Cu K*_α_* radiation. The diffraction pattern was indexed and the total number of runs and images was based on the strategy calculation from the program CrysAlisPro system (CCD 43.92a 64-bit (release 05-10-2023)). The maximum resolution that was achieved was *Θ* = 68.24^°^ (0.83 Å).

The unit cell was refined using CrysAlisPro 1.171.43.92a (Rigaku OD, 2023) on 4380 reflections, 10% of the observed reflections.

Data reduction, scaling and absorption corrections were performed using CrysAlisPro 1.171.43.92a (Rigaku OD, 2023). The final completeness is 98.98 % out to 68.24^°^ in *Θ*. A numerical absorption correction based on gaussian integration over a multifaceted crystal model was performed using CrysAlisPro 1.171.42.74a (Rigaku Oxford Diffraction, 2022). An empirical absorption correction using spherical harmonics, implemented in SCALE3 ABSPACK scaling algorithm was also applied. The absorption coefficient *μ* of this material is 3.874 mm^-1^ at this wavelength (*λ* = 1.54184Å) and the minimum and maximum transmissions are 0.396 and 1.000.

The structure was solved and the space group *P*2_1_/*n* (# 14) determined by the ShelXT (Sheldrick, 2015) structure solution program using iterative methods and refined by full matrix least squares minimisation on ***F*^2^** using version of olex2.refine 1.5-alpha (Bourhis et al., 2015). All atoms, even hydrogens were refined anisotropically. Hydrogen atom positions were calculated geometrically and refined using the riding model. Refinement was by using NoSpherA2, an implementation of non-spherical atom-form-factors (F. Kleemiss, H. Puschmann, O. Dolomanov, S.Grabowsky - https://doi.org/10.1039/D0SC05526C – 2020). NoSpherA2 implementation of HAR makes use of tailor-made aspherical atomic form factors calculated from a Hirshfeld-partitioned electron density (ED) not from spherical-atom form factors. The ED was calculated from a Gaussian basis set single determinant SCF wavefunction from DFT using selected functionals for a fragment of this crystal. This fragment was embedded in an electrostatic crystal field by employing cluster charges. The following options were used: SOFTWARE: ORCA PARTITIONING: NoSpherA2 INT ACCURACY: Normal METHOD: PBE BASIS SET: def2-SVP CHARGE: 0 MULTIPLICITY: 1 DATE: 2024-06-28.

There is a single formula unit in the asymmetric unit, which consists of a phenazine-1,9-diol (C_12_H_8_N_2_O_2_) molecule, a 9-hydroxy-5-methylphenazin-1(*5H*)-one molecule (C_13_H_10_N_2_O_2_) and a chloroform molecule. Z is 4 and Z' is 1.

**Supplemental Figure SF18.** Thermal ellipsoidal (30% probability, including-H atoms) representation of the asymmetric unit of the crystal structure for **1** and **1b** heterodimer. The formula unit comprises of three different entities: a phenazine-1,9-diol (C_12_H_8_N_2_O_2_) molecule, a 9-hydroxy-5-methylphenazin-1(*5H*)-one molecule (C_13_H_10_N_2_O_2_) and a chloroform molecule. The hydrogen atom positions were refined freely using Hirshfeld atoms, and although they are strongly localized the bonding appears to be delocalized over both the entities.


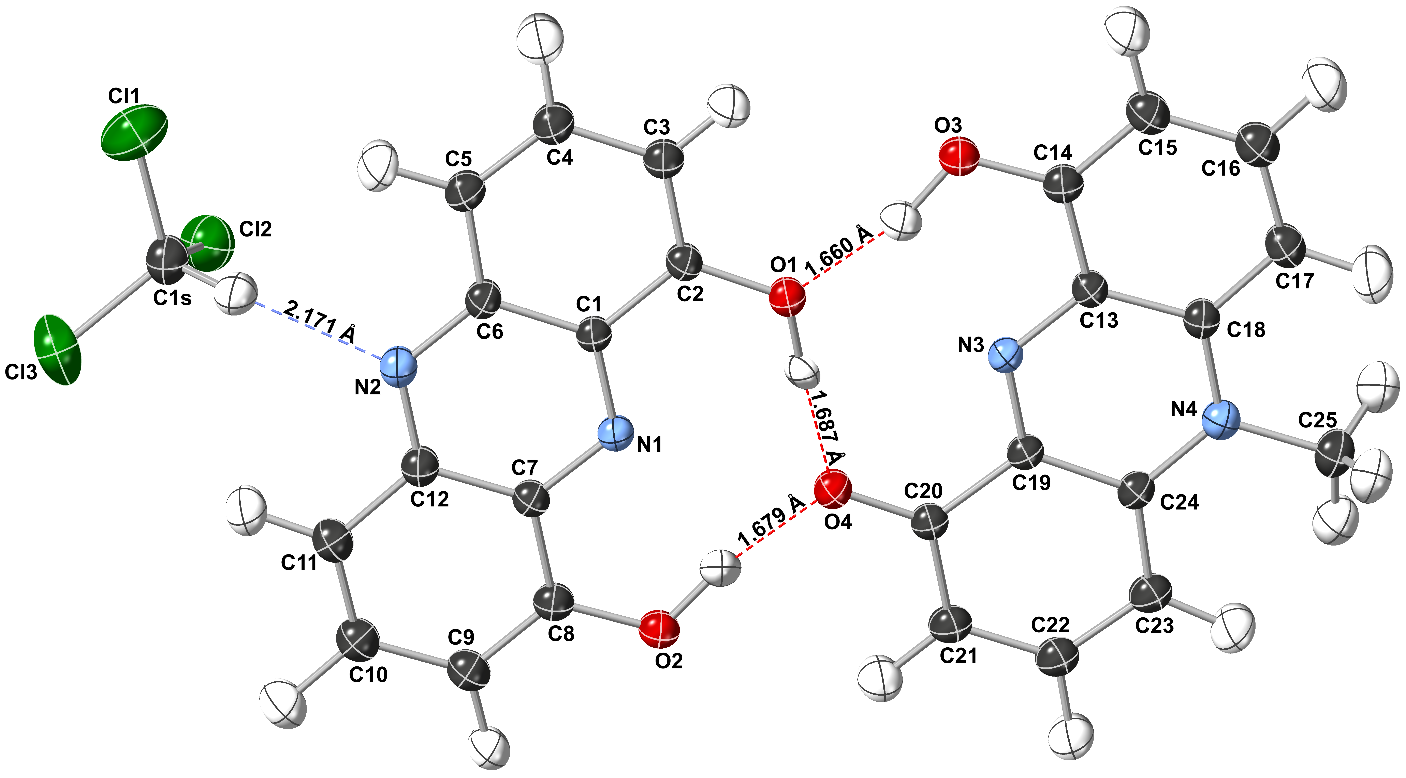


**Supplemental Figure SF19.** Thermal ellipsoidal (30% probability, including-H atoms) representation of the asymmetric unit of the crystal structure for **1** and **1b** heterodimer. The formula unit comprises of three different entities: a phenazine-1,9-diol (C_12_H_8_N_2_O_2_) molecule, a 9-hydroxy-5-methylphenazin-1(*5H*)-one molecule (C_13_H_10_N_2_O_2_) and a chloroform molecule.


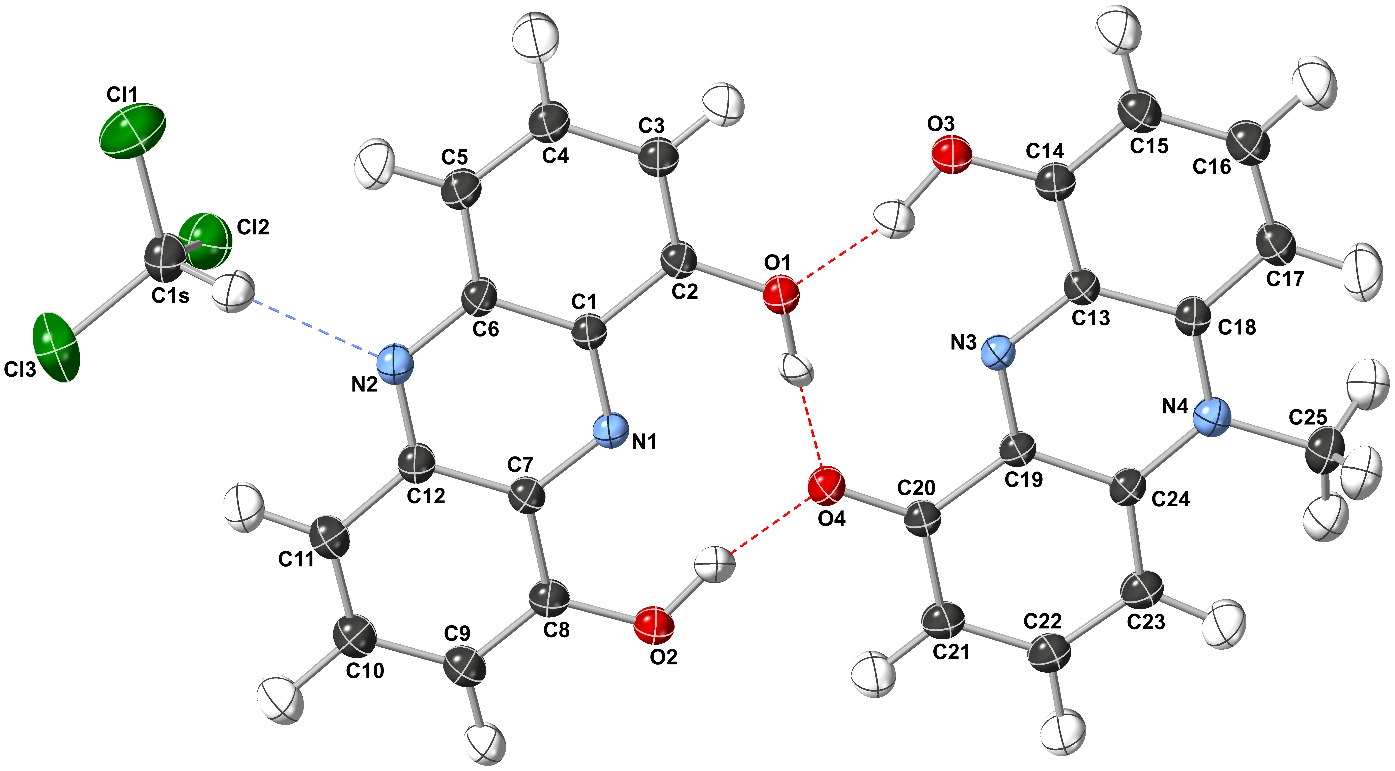


**Supplemental Figure SF20.** Molecular packing for **1** and **1b** heterodimer .


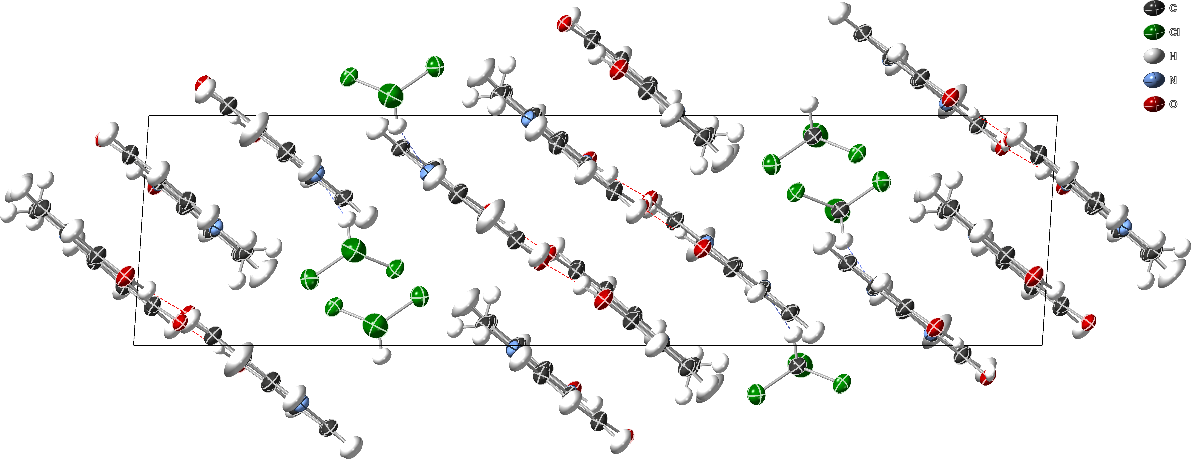


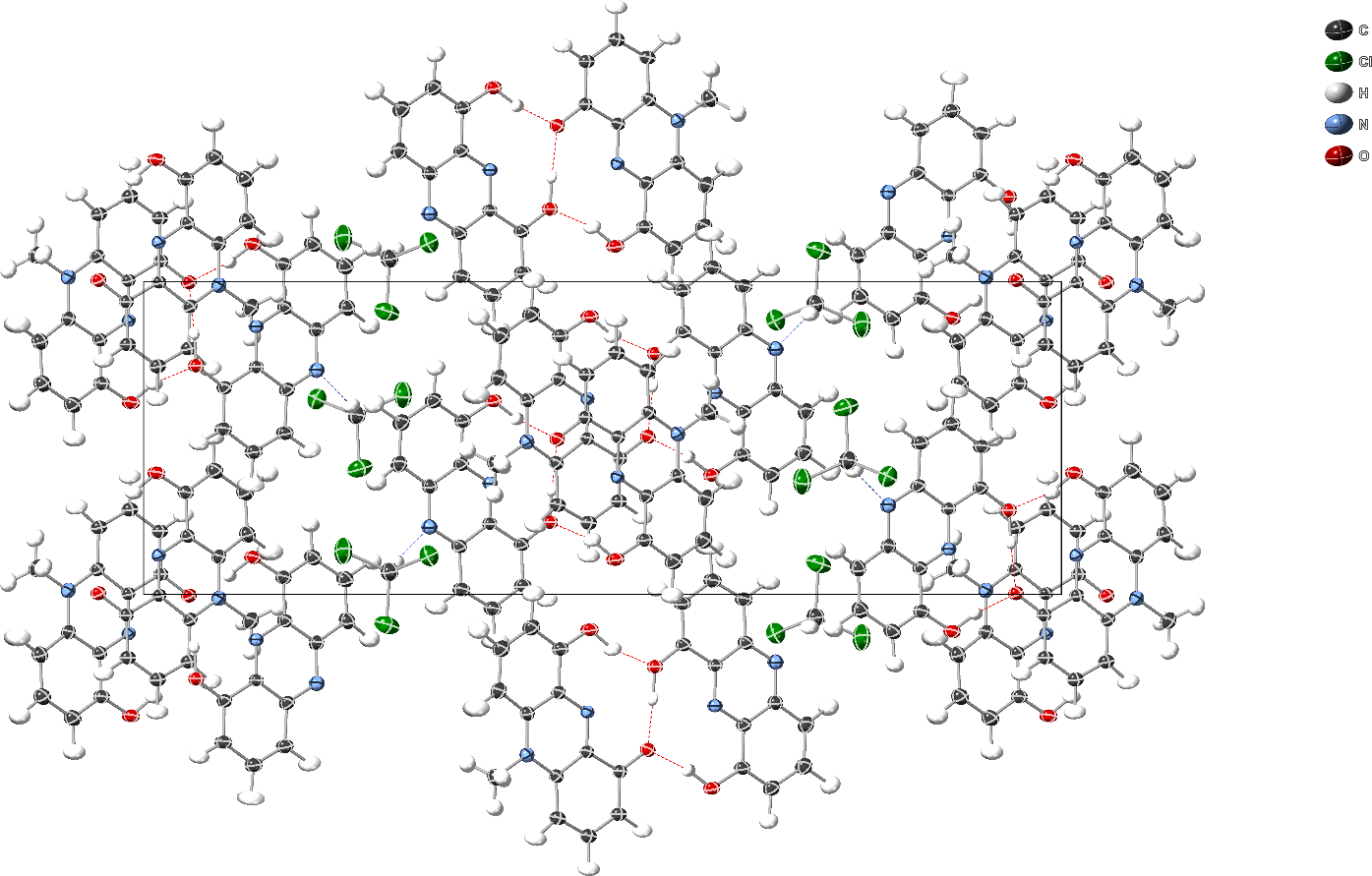


**Supplemental Figure SF21.** Data plots: diffraction data on **1** and **1b** heterodimer.

| 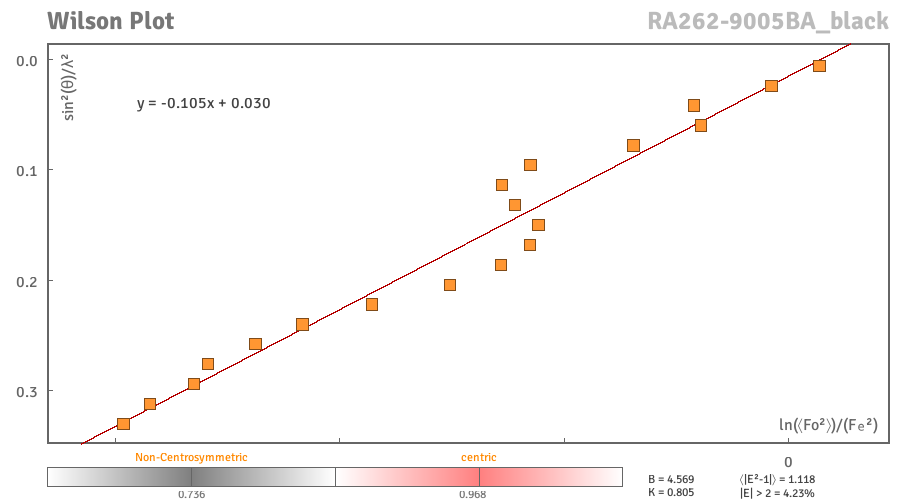 | 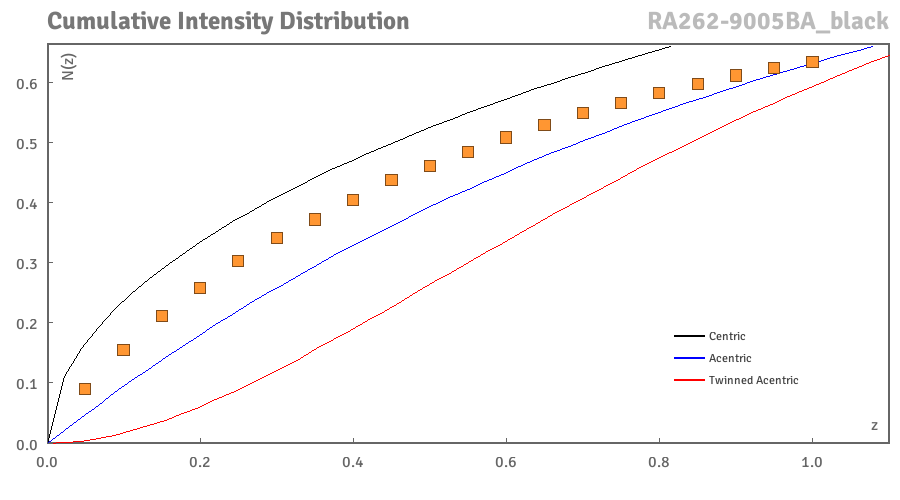 |
| --- | --- |
| 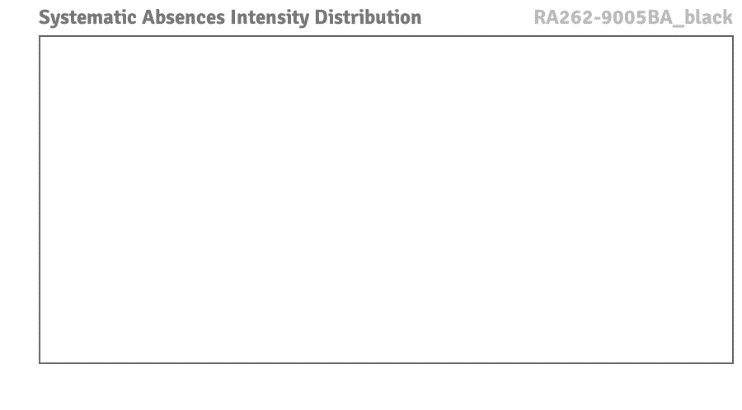 | 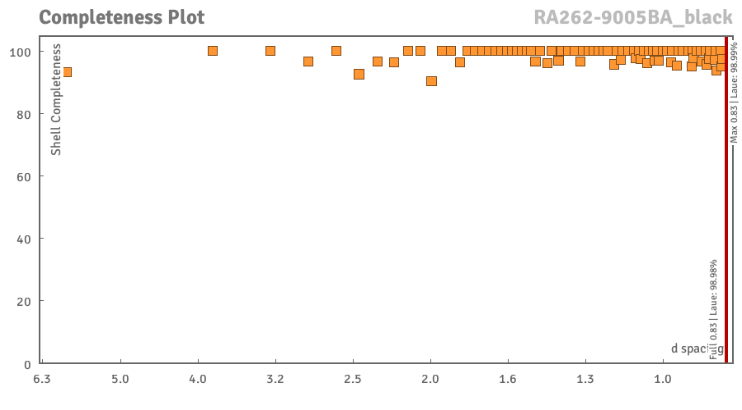 |
| 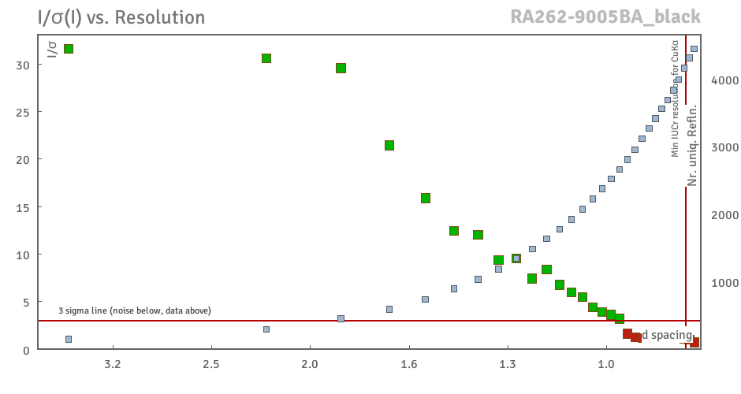 |  |

**Data Plots: Refinement and Data**

| 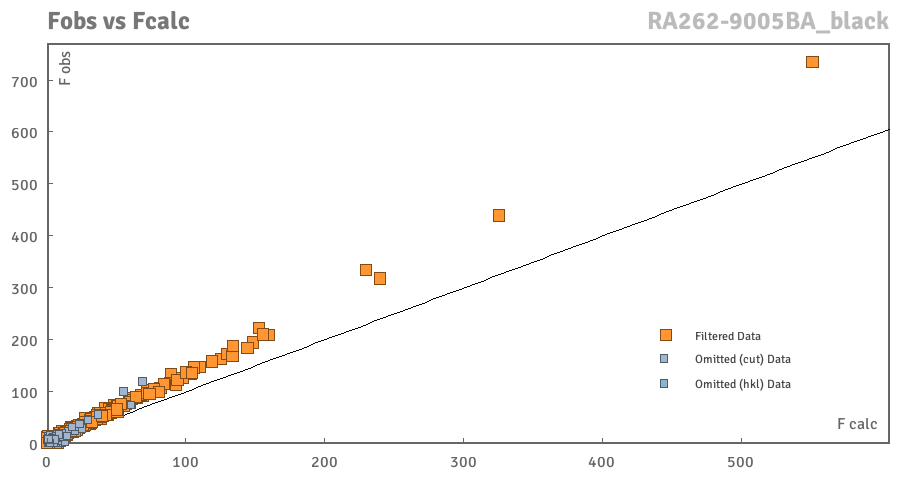 | 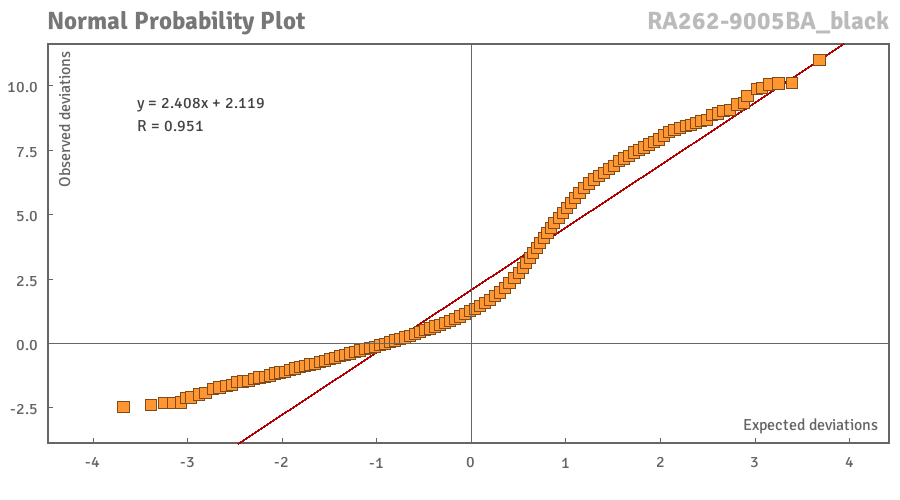 |
| --- | --- |

**Reflection Statistics**

| Total reflections (after filtering) | 42261 | Unique reflections | 4309 |
| --- | --- | --- | --- |
| Completeness | 0.99 | Mean I/*σ* | 8.36 |
| hkl_max_ collected | (8, 12, 35) | hkl_min_ collected | (-9, -11, -36) |
| hkl_max_ used | (9, 12, 36) | hkl_min_ used | (-9, 0, 0) |
| Lim d_max_ collected | 20.0 | Lim d_min_ collected | 0.83 |
| d_max_ used | 9.73 | d_min_ used | 0.83 |
| Friedel pairs | 5514 | Friedel pairs merged | 1 |
| Inconsistent equivalents | 2 | R_int_ | 0.109 |
| R_sigma_ | 0.0631 | Intensity transformed | 0 |
| Omitted reflections | 377 | Omitted by user (OMIT hkl) | 0 |
| Multiplicity | (3192, 2779, 2584, 1675, 1283, 830, 528, 288, 121, 75, 27, 12, 7, 5) | Maximum multiplicity | 35 |
| Removed systematic absences | 0 | Filtered off (Shel/OMIT) | 400 |

**Supplemental Figure SF22.** Crystal data and experimental details for **1b** dimer.


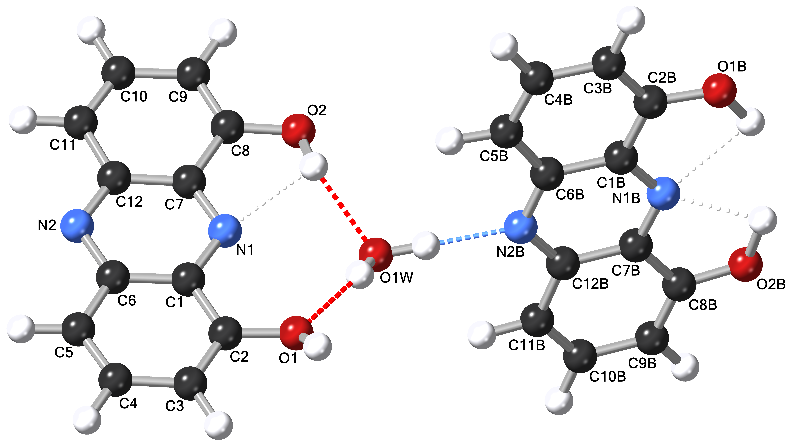


**Experimental.** Single red plate/needle-shaped crystals of 9005BA-red were chosen from the sample as supplied. A suitable crystal with dimensions 0.05 × 0.03 × 0.02 mm^3^ was selected and mounted on a loop with IVH oil on a XtaLAB AFC11 (RCD3): quarter-chi single diffractometer. The crystal was kept at a steady *T* = 173.01(11) K during data collection. The structure was solved with the ShelXT (Sheldrick, 2015) solution program and by using Olex2 1.5-alpha (Dolomanov et al., 2009). The model was refined with olex2.refine 1.5-alpha (Bourhis et al., 2015) using full matrix least squares minimisation on ***F*^2^**.

**Crystal Data.** C_24_H_18_N_4_O_5_, *M_r_* = 442.434, monoclinic, *P*2_1_/*n* (No. 14), a = 8.499(6) Å, b = 16.173(12) Å, c = 14.619(5) Å, *b* = 92.97(4)^°^, *a* = *g* = 90^°^, *V* = 2007(2) Å^3^, *T* = 173.01(11) K, *Z* = 4, *Z'* = 1, *m*(Cu K*_a_*) = 0.875, 15970 reflections measured, 2893 unique (R_int_ = 0.2790) which were used in all calculations. The final *wR_2_* was 0.3139 (all data) and *R_1_* was 0.1101 (I≥2 *s*(I)).

| **Compound** | 9005BA-red |
| --- | --- |
|  |  |
| Formula | C_24_H_18_N_4_O_5_ |
| *D_calc._*/ g cm^-3^ | 1.464 |
| *m*/mm^-1^ | 0.875 |
| Formula Weight | 442.434 |
| Color | red |
| Shape | needle-shaped |
| Size/mm^3^ | 0.05×0.03×0.02 |
| *T*/K | 173.01(11) |
| Crystal System | monoclinic |
| Space Group | *P*2_1_/*n* |
| *a*/Å | 8.499(6) |
| *b*/Å | 16.173(12) |
| *c*/Å | 14.619(5) |
| *a*/^°^ | 90 |
| *b*/^°^ | 92.97(4) |
| *g*/^°^ | 90 |
| V/Å^3^ | 2007(2) |
| *Z* | 4 |
| *Z'* | 1 |
| Wavelength/Å | 1.54184 |
| Radiation type | Cu K*_a_* |
| *Q_min_*/^°^ | 4.08 |
| *Q_max_*/^°^ | 61.29 |
| Measured Refl's. | 15970 |
| Indep't Refl's | 2893 |
| Refl's I≥2 *s*(I) | 661 |
| *R*_int_ | 0.2790 |
| Parameters | 312 |
| Restraints | 269 |
| Largest Peak | 1.1518 |
| Deepest Hole | -1.0361 |
| GooF | 0.9661 |
| *wR_2_* (all data) | 0.3139 |
| *wR_2_* | 0.1977 |
| *R_1_* (all data) | 0.3802 |
| *R_1_* | 0.1101 |

**Supplemental Figure SF23.** Structural quality indicators for **1b** dimer.

**Structure Quality Indicators**

| **Reflections:** | 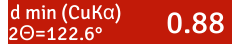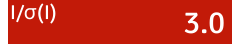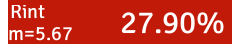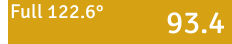 |
| --- | --- |
| **Refinement:** | 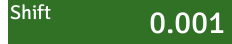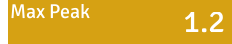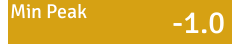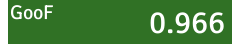 |

A red needle-shaped-shaped crystal with dimensions 0.05 × 0.03 × 0.02 mm^3^ was mounted on a loop with IVH oil. Data were collected using a XtaLAB AFC11 (RCD3): quarter-chi single diffractometer operating at *T* = 173.01(11) K.

Data were measured using *w* scans with Cu K*_a_* radiation. The diffraction pattern was indexed and the total number of runs and images was based on the strategy calculation from the program CrysAlisPro system (CCD 43.92a 64-bit (release 05-10-2023)). The maximum resolution that was achieved was *Q* = 61.29^°^ (0.88 Å).

The unit cell was refined using CrysAlisPro 1.171.43.121a (Rigaku OD, 2024) on 467 reflections, 3% of the observed reflections.

Data reduction, scaling and absorption corrections were performed using CrysAlisPro 1.171.43.121a (Rigaku OD, 2024). The final completeness is 93.41 % out to 61.29^°^ in *Q*. A multi-scan absorption correction using spherical harmonics was performed using CrysAlisPro 1.171.43.121a (Rigaku Oxford Diffraction, 2024). The absorption coefficient *m* of this material is 0.875 mm^-1^ at this wavelength (*l* = 1.54184Å) and the minimum and maximum transmissions are 0.666 and 1.000.

The structure was solved and the space group *P*2_1_/*n* (# 14) determined by the ShelXT (Sheldrick, 2015) structure solution program using dual methods and refined by full matrix least squares minimisation on ***F*^2^** using version of olex2.refine 1.5-alpha (Bourhis et al., 2015). All non-hydrogen atoms were refined anisotropically. Most hydrogen atom positions were calculated geometrically and refined using the riding model, but some hydrogen atoms were refined freely.

There is a single formula unit in the asymmetric unit, which is represented by the reported sum formula. In other words: Z is 4 and Z' is 1. The moiety formula is 2(C12 H8 N2 O2), H2 O.

Refinement using NoSpherA2, an implementation ofNOn-SPHERical Atom-form-factors in Olex2.Please cite:F. Kleemiss et al. Chem. Sci. DOI 10.1039/D0SC05526C - 2021NoSpherA2 implementation of HAR makes use oftailor-made aspherical atomic form factors calculatedon-the-fly from a Hirshfeld-partitioned electron density (ED) - not fromspherical-atom form factors.The ED is calculated from a gaussian basis set single determinant SCFwavefunction - either Hartree-Fock or DFT using selected funtionals- for a fragment of the crystal.This fragment can be embedded in an electrostatic crystal field by employing cluster chargesor modelled using implicit solvation models, depending on the software used.The following options were used: SOFTWARE: ORCA 5.0 PARTITIONING: NoSpherA2 INT ACCURACY: Normal METHOD: PBE BASIS SET: def2-SVP CHARGE: 0 MULTIPLICITY: 1 SOLVATION: Methanol DATE: 2025-04-11_15-22-37

**Supplemental Figure SF24.** The formula unit consists of two a phenazine-1,9-diol (C_12_H_8_N_2_O_2_) molecules **1b** dimer bridged by a water molecule. The atoms are depicted as spheres with arbitrary radii.


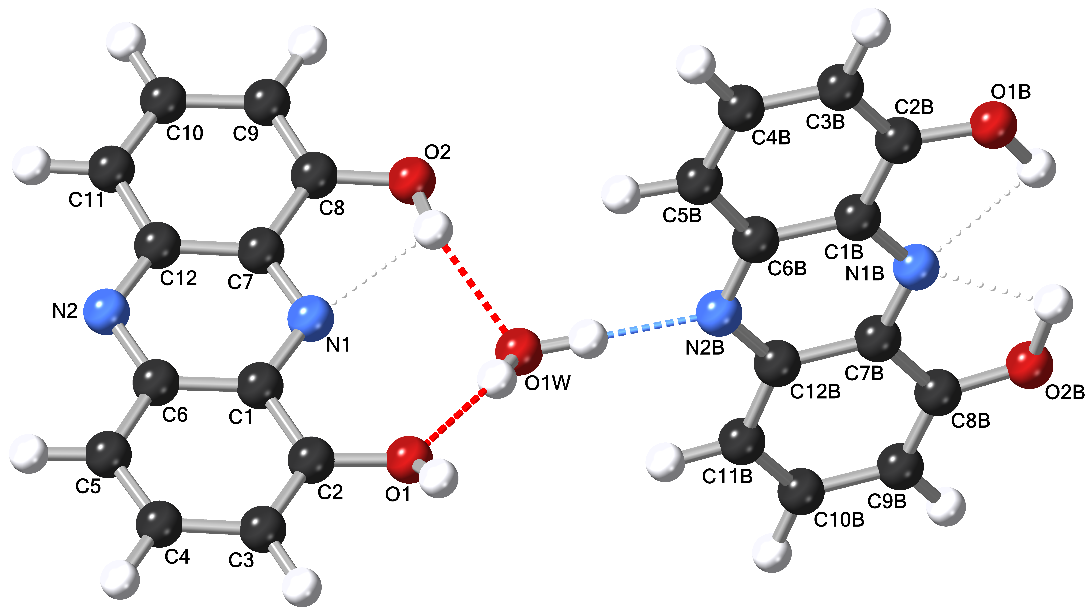


**Supplemental Figure SF25.** Molecular packing for **1b** dimer viewed along the a-axis showing two distinct chains and the extended nature of the hydrogen bonding**.**


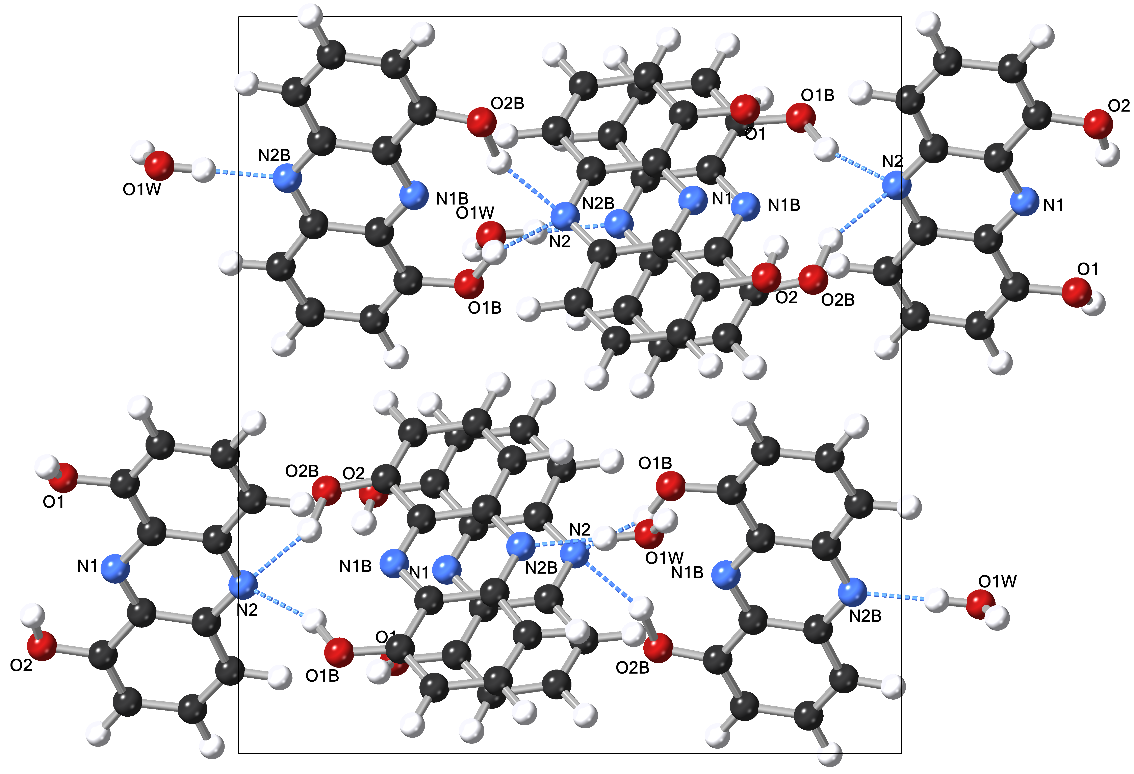


**Supplemental Figure SF26.** Molecular packing for **1b** dimer viewed along the 110-axis showing the mixture of pipi interactions and hydrogen bonding


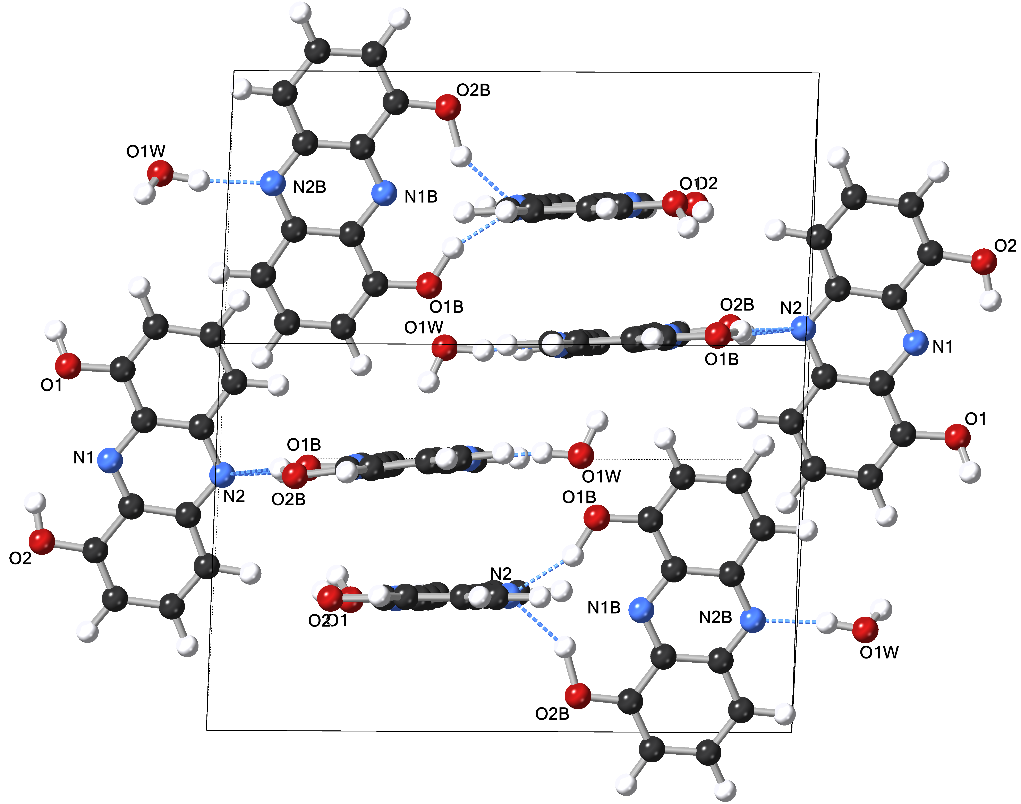


**Supplemental Figure SF27.** Molecular packing for **1b** dimer viewed along the 001-axis showing the interlocked, herringbone arrangement of infinite hydrogen bonded chains


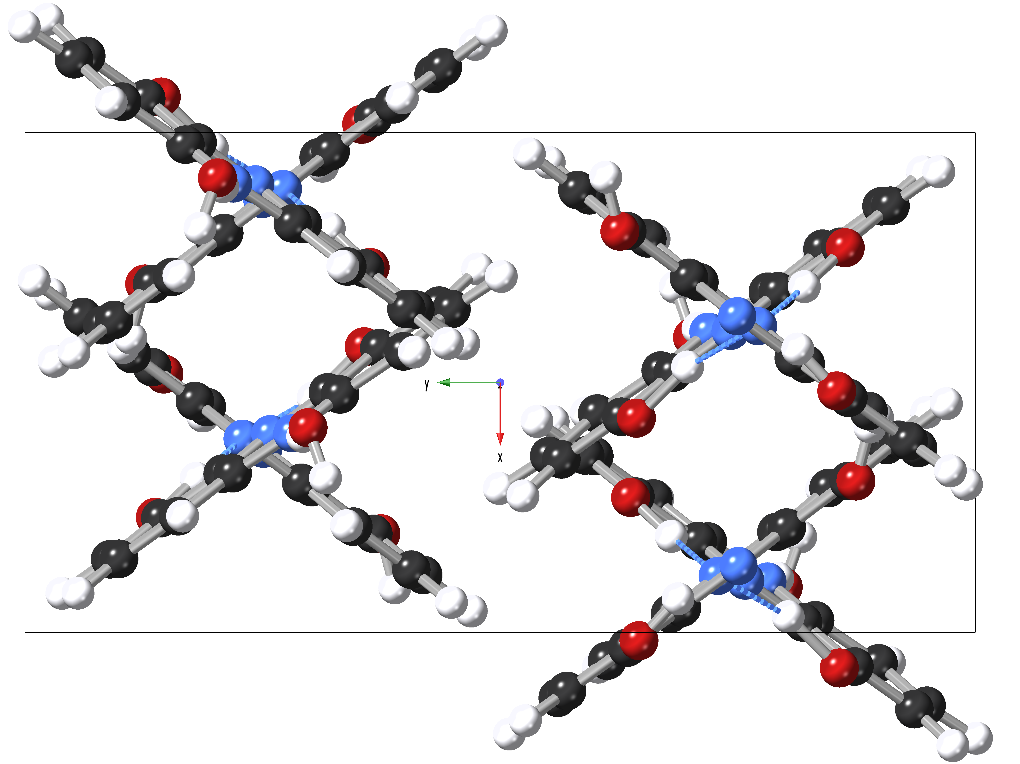


**Supplemental Figure SF28.** Data plots: diffraction data on **1b** dimer.

**Data Plots: Diffraction Data**

| 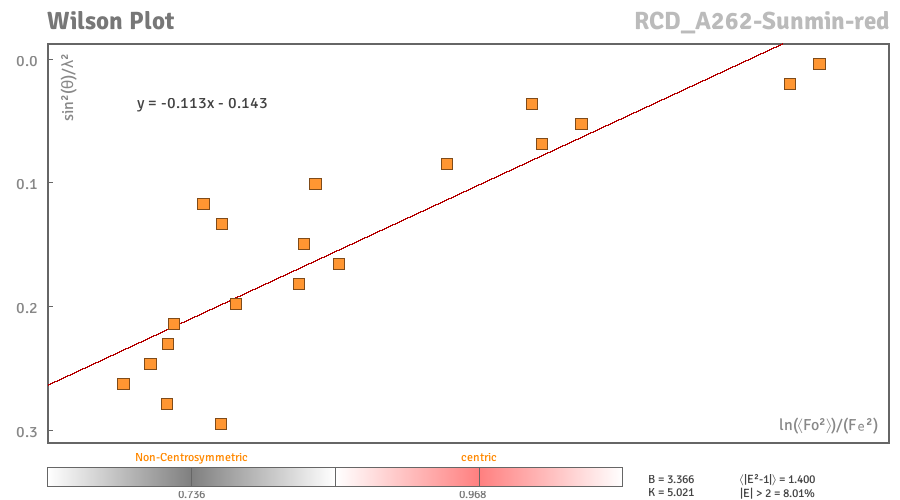 | 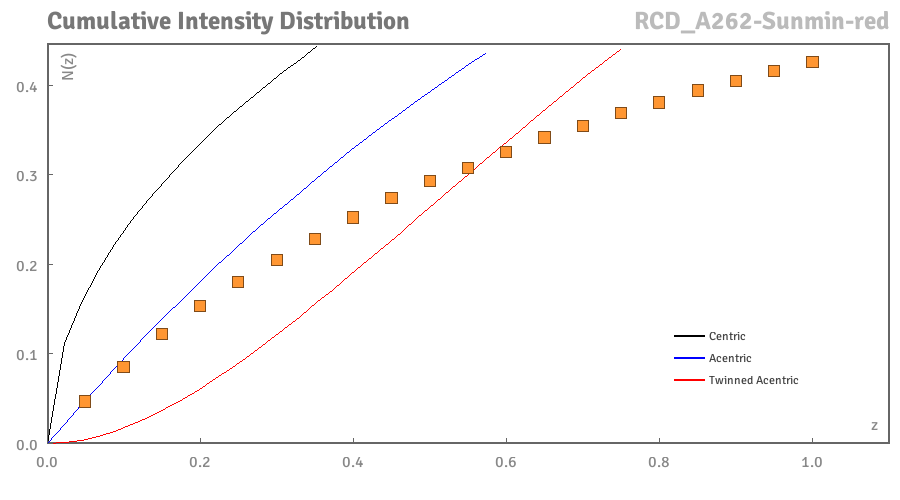 |
| --- | --- |
| 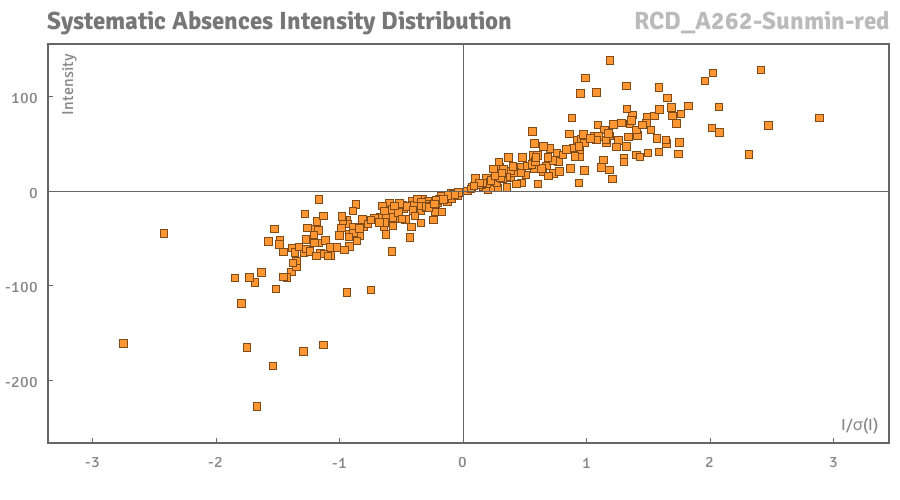 | 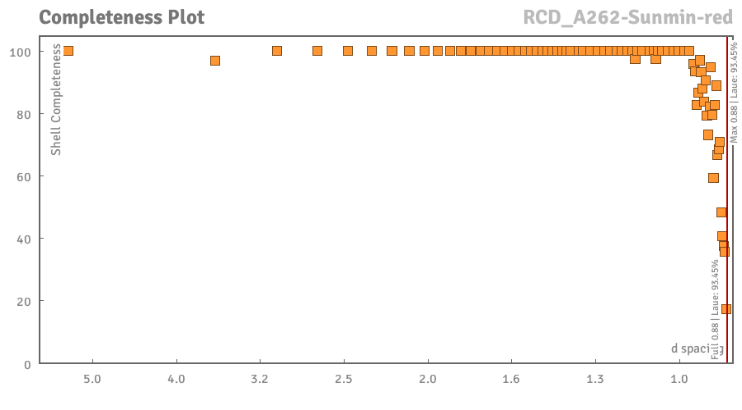 |
| 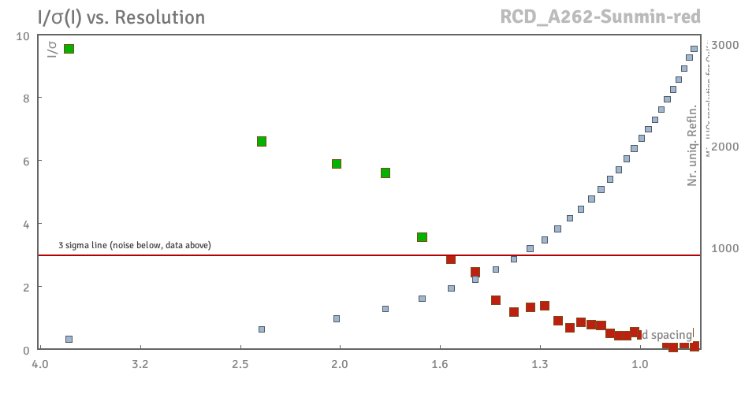 |  |

**Data Plots: Refinement and Data**

| 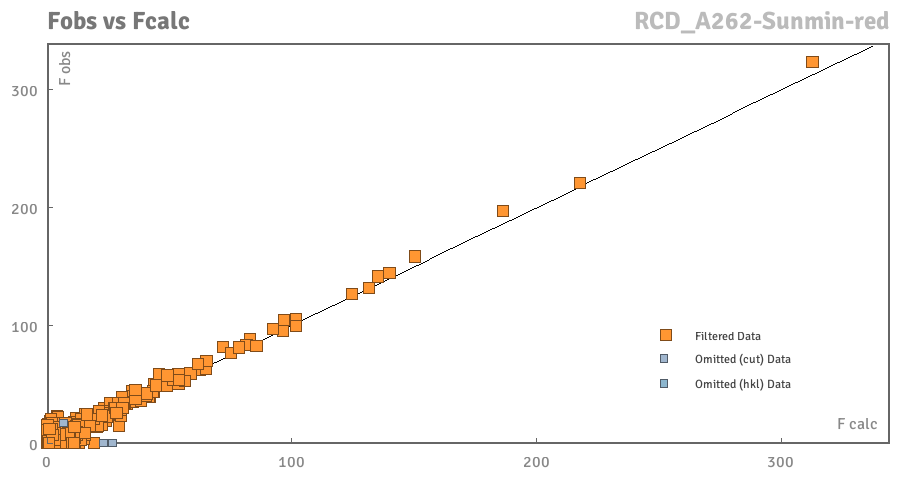 | 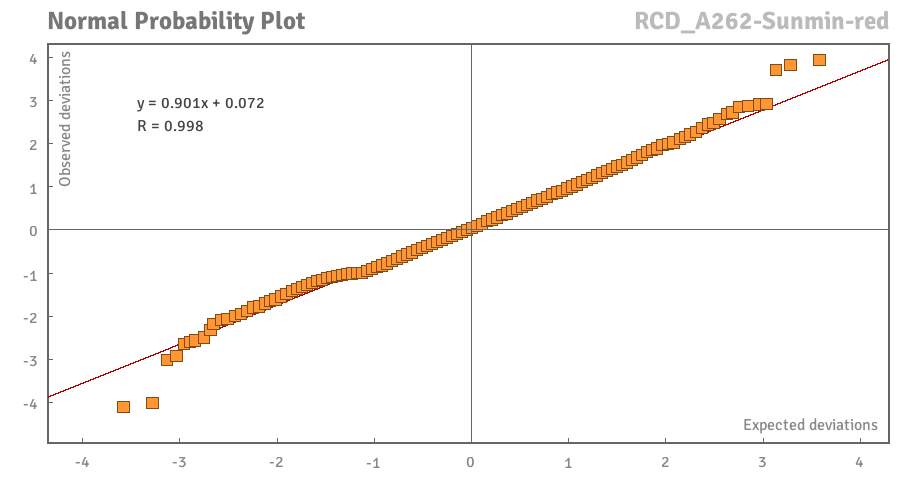 |
| --- | --- |

**Reflection Statistics**

| Total reflections (after filtering) | 16421 | Unique reflections | 2894 |
| --- | --- | --- | --- |
| Completeness | 0.934 | Mean I/*s* | 1.8 |
| hkl_max_ collected | (9, 16, 15) | hkl_min_ collected | (-9, -17, -16) |
| hkl_max_ used | (9, 17, 16) | hkl_min_ used | (-9, 0, 0) |
| Lim d_max_ collected | 100.0 | Lim d_min_ collected | 0.77 |
| d_max_ used | 16.17 | d_min_ used | 0.88 |
| Friedel pairs | 2921 | Friedel pairs merged | 1 |
| Inconsistent equivalents | 0 | R_int_ | 0.2788 |
| R_sigma_ | 0.3375 | Intensity transformed | 0 |
| Omitted reflections | 0 | Omitted by user (OMIT hkl) | 7 |
| Multiplicity | (3325, 2130, 1598, 575, 205, 81, 26, 7) | Maximum multiplicity | 18 |
| Removed systematic absences | 458 | Filtered off (Shel/OMIT) | 0 |

**Supplementary Figures and Table References**

CrysAlisPro (ROD), Rigaku Oxford Diffraction, Poland.

CrysAlisPro Software System, Rigaku Oxford Diffraction, (2023).

L.J. Bourhis and O.V. Dolomanov and R.J. Gildea and J.A.K. Howard and H. Puschmann, The Anatomy of a Comprehensive Constrained, Restrained, Refinement Program for the Modern Computing Environment - Olex2 Disected, *Acta Cryst. A*, (2015), **A71**, 59-71.

O.V. Dolomanov and L.J. Bourhis and R.J. Gildea and J.A.K. Howard and H. Puschmann, Olex2: A complete structure solution, refinement and analysis program, *J. Appl. Cryst.*, (2009), **42**, 339-341.

Sheldrick, G.M., ShelXT-Integrated space-group and crystal-structure determination, *Acta Cryst.*, (2015), **A71**, 3-8.
